# A UBE3A‐UTX Proteostasis‐Epigenetic Axis Governs Hypothalamic Energy Homeostasis

**DOI:** 10.1002/advs.78567

**Published:** 2026-10-29

**Authors:** Junekyoung Lee, Jaeyoung Yoo, Young A. Kim, Zhuodu Wei, Yunseo Lee, Woo‐Jeong Shon, Je Kyung Seong, Seunghee Lee

**Affiliations:** ^1^ College of Pharmacy Seoul National University Seoul Republic of Korea; ^2^ Department of Biopharmaceutical Sciences Cheongju University Cheongju Republic of Korea; ^3^ Korea Mouse Phenotyping Center Seoul National University Seoul Republic of Korea; ^4^ Research Institute of Pharmaceutical Sciences and Natural Products Research Institute Seoul National University Seoul Republic of Korea; ^5^ Seoul National University Energy Initiative Seoul National University Seoul Republic of Korea

**Keywords:** ER stress, hypothalamic neurons, obesity, UBE3A, UTX

## Abstract

Energy homeostasis requires integration of metabolic and stress signals within hypothalamic circuits, yet the role of neuronal proteostasis remains unclear. Ubiquitin protein ligase E3A (UBE3A) regulates the stability of lysine demethylase 6A (KDM6A), also known as ubiquitously transcribed tetratricopeptide repeat containing, X chromosome (UTX). Hypothalamic neuronal deletion of *Ube3a* results in hyperphagia, obesity, hyperleptinemia, and impaired glucose homeostasis under high‐fat diet conditions; selective deletion in agouti‐related peptide (AgRP) neurons is sufficient to recapitulate these phenotypes. UBE3A promotes UTX degradation, whereas UBE3A loss stabilizes UTX and decreases repressive histone H3 lysine 27 trimethylation (H3K27me3). Integrated transcriptomic and epigenomic analyses implicate UTX and activating transcription factor 4 (ATF4) in altered endoplasmic reticulum (ER) stress‐responsive transcriptional programs, although their causal contribution to the metabolic phenotype remains to be established. Treatment with GSK‐J4, an inhibitor of KDM6‐family demethylases, increases hypothalamic H3K27me3 levels and improves metabolic outcomes in *Ube3a*‐deficient mice, although the specific contribution of hypothalamic UTX inhibition to these effects remains to be established. These findings support a link between neuronal proteostasis, chromatin regulation, and systemic metabolic homeostasis and warrant further investigation of the relevance of UTX‐associated pathways to metabolic dysfunction in a subset of individuals with Angelman syndrome.

## Graphical abstract

Ubiquitin protein ligase E3A (UBE3A) regulates hypothalamic energy homeostasis and promotes degradation of the histone demethylase UTX (KDM6A). Neuronal UBE3A loss increases susceptibility to diet‐induced obesity and is accompanied by UTX accumulation, reduced histone H3 lysine 27 trimethylation, and altered endoplasmic reticulum stress‐responsive gene expression. These findings suggest a link between neuronal proteostasis, chromatin regulation, and systemic metabolism.

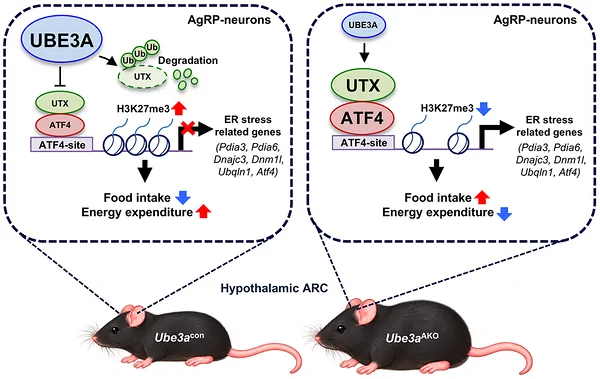

## 1 Introduction

Obesity results from chronic disruption of energy homeostasis and represents a major global health burden. The hypothalamus plays a central role in coordinating feeding behavior and systemic metabolism by integrating hormonal and nutrient‐derived signals [1, 2]. Among its neuronal populations, agouti‐related peptide (AgRP) neurons act as key orexigenic drivers that dynamically respond to metabolic cues to regulate appetite and glucose homeostasis [2, 3, 4]. Although extensive studies have defined the hormonal and neural circuits controlling energy balance, the molecular mechanisms by which hypothalamic neurons integrate stress and metabolic signals remain incompletely understood.

Ubiquitin protein ligase E3A (UBE3A), also known as E6‐associated protein (E6AP), contains a homologous to E6AP C‐terminus (HECT) domain and mediates the ubiquitin‐proteasome‐dependent degradation of specific substrate proteins [5, 6]. In the brain, the paternally inherited *Ube3a* allele is silenced by genomic imprinting, leaving expression primarily from the maternal allele in postmitotic neurons [7, 8]. Altered UBE3A expression or function is implicated in neurodevelopmental disorders, including Angelman syndrome (AS) and Dup15q syndrome [9, 10]. AS is characterized by intellectual disability, seizures, and speech impairment. Obesity has also been reported in a subset of individuals with AS, with its prevalence varying according to the underlying molecular subtype [11, 12]. Although the neurodevelopmental consequences of UBE3A loss have been extensively studied, how UBE3A dysfunction contributes to metabolic abnormalities remains incompletely understood.

Clinical studies have reported hyperphagia and early‐onset obesity in a subset of individuals with AS [13, 14]. Individuals with AS due to paternal uniparental disomy (pUPD) have been reported to have higher birth weight and greater birth length compared to other genetic subtypes [14]. Approximately one‐third of adolescents and adults with AS are overweight or obese, with a higher prevalence in females [15]. Consistent with human findings, *Ube3a* mutant mice display juvenile‐ and adult‐onset obesity [16, 17]. Although UBE3A has been implicated in metabolic regulation [18], the mechanisms by which it influences central control of energy balance remain incompletely understood. We hypothesized that UBE3A controls hypothalamic metabolic homeostasis through post‐translational regulation of factors that integrate stress and nutrient signals.

Endoplasmic reticulum (ER) stress in hypothalamic neurons has emerged as a pivotal mechanism linking overnutrition to leptin resistance and obesity [19, 20]. Nutrient overload or inflammation activates the unfolded protein response (UPR) to restore proteostasis [21]. When chronically engaged, UPR signaling perturbs leptin and insulin signaling pathways by inducing negative regulators such as suppressor of cytokine signaling 3 (SOCS3) and protein tyrosine phosphatase 1B (PTP1B) [22] and by activating inflammatory kinases, including inhibitor of nuclear factor‐κB kinase subunit β (IKKβ) and c‐Jun N‐terminal kinase (JNK) [21, 23, 24]. Persistent ER stress also interferes with neuropeptide processing, reducing α‐melanocyte‐stimulating hormone (α‐MSH) levels despite unchanged proopiomelanocortin (*Pomc)* mRNA [25]. Thus, regulation of ER stress‐responsive transcription in hypothalamic neurons is essential for maintaining leptin sensitivity and energy homeostasis. Given that UBE3A has been shown to interact with transcriptional coactivators and chromatin remodelers [18], we hypothesized that it could link ER proteostasis with epigenetic control of stress‐responsive gene expression in neurons.

Epigenetic mechanisms, including histone modifications, play central roles in transcriptional regulation, neuronal differentiation, and synaptic plasticity [26, 27, 28]. Dynamic changes in histone methylation and acetylation coordinate gene programs required for neuronal and metabolic adaptation [29, 30, 31, 32]. Beyond its canonical role in protein degradation, UBE3A interacts with chromatin‐remodeling complexes and modulates histone modification states [33, 34, 35, 36]. Dysregulated UBE3A expression may therefore reshape the neuronal epigenome and alter transcriptional networks that couple stress responses to energy balance.

Here, we identify lysine demethylase 6A (KDM6A), also known as ubiquitously transcribed tetratricopeptide repeat containing, X chromosome (UTX), as a previously unrecognized substrate of UBE3A in the hypothalamus. We show that hypothalamic UBE3A deficiency disrupts energy homeostasis and is accompanied by UTX accumulation, reduced histone H3 lysine 27 trimethylation (H3K27me3), and altered ER stress‐responsive gene expression. Integrated transcriptomic, epigenomic, and biochemical analyses further implicate UTX and activating transcription factor 4 (ATF4) in this transcriptional response, although their causal contribution to the metabolic phenotype remains to be established. These findings support a link between neuronal proteostasis, chromatin regulation, and energy homeostasis and warrant further investigation of UTX‐associated pathways in metabolic dysfunction in AS.

## 2 Results

### 2.1 UBE3A is Expressed in Metabolically Relevant Hypothalamic Neurons and Responds to Nutritional State

To define the cellular context in which UBE3A may regulate energy balance, we first examined its distribution across key neuronal populations in the arcuate nucleus (ARC) of the hypothalamus. In female mice, UBE3A was detected in the majority of agouti‐related peptide (AgRP), proopiomelanocortin (POMC), and growth hormone‐releasing hormone (GHRH) neurons, with a high proportion of AgRP neurons expressing UBE3A (Figure 1a–c). A similar distribution pattern was observed in male mice, with UBE3A broadly expressed in AgRP, POMC, and GHRH neurons (Figure S1). These findings establish that UBE3A is expressed in neuronal populations involved in the regulation of feeding behavior and systemic metabolism.

**FIGURE 1:**
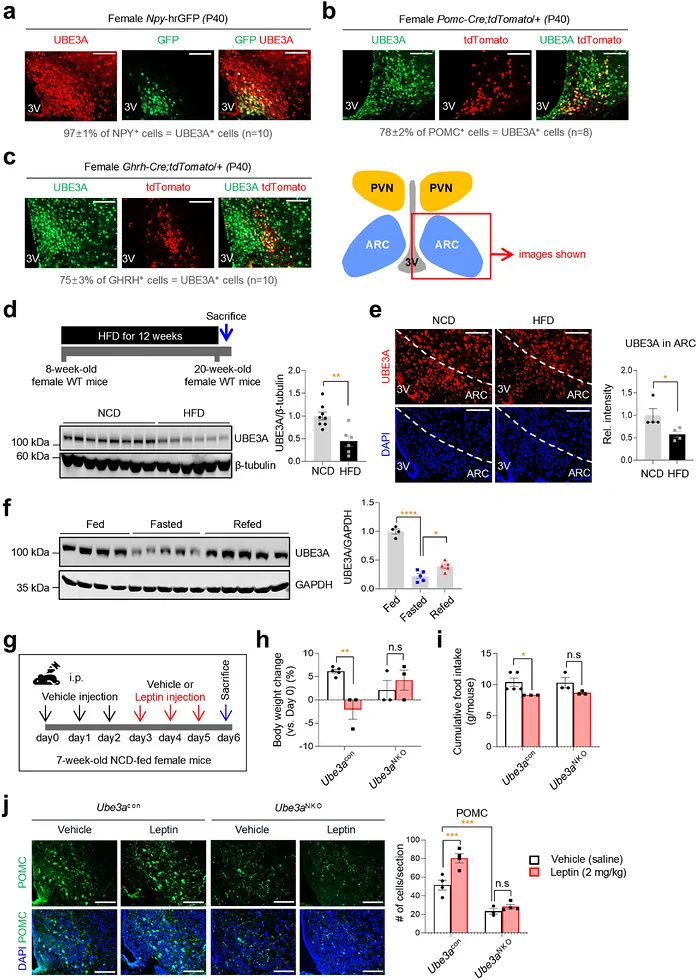
Hypothalamic UBE3A dynamically responds to nutritional state and regulates leptin‐responsive neuronal programs. (a–c) Colocalization of UBE3A immunoreactivity with GFP in *Npy‐*hrGFP neurons (a) and with tdTomato in *Pomc‐*Cre;*tdTomato*/+ neurons (b) and *Ghrh‐*Cre;*tdTomato*/+ neurons (c) in the ARC of female mice at postnatal day 40 (P40). Quantification shows the proportion of UBE3A‐positive cells within NPY, POMC, and GHRH neuronal populations. The number of analysed images is indicated in parentheses. (d) Immunoblot analysis and quantification of UBE3A protein levels in hypothalamic lysates from WT mice fed either NCD (*n* = 8 mice) or HFD (*n* = 6 mice) for 12 weeks. Band intensities were quantified using ImageJ and normalized to β‐tubulin. (e) Representative immunohistochemistry images and quantification of UBE3A fluorescence intensity in the ARC of WT mice fed NCD or HFD (*n* = 4 mice per group). Fluorescence intensity was normalized to the NCD group, which was set to 1. (f) Immunoblot analysis and quantification of hypothalamic UBE3A protein levels in WT mice under fed, 24‐h fasted, and 6‐h refed conditions (*n* = 4–5 mice per group). Band intensities were quantified using ImageJ and normalized to GAPDH. (g) Experimental scheme for leptin administration. Mice received vehicle injections for 3 consecutive days, followed by daily injections of vehicle or leptin (2 mg/kg, intraperitoneally) for an additional 3 consecutive days. (h, i) The percentage of changes in body weight relative to Day 0 (h) and cumulative food intake (g per mouse) (i) during the indicated treatment periods (*n* = 3–5 mice per group). (j) Representative immunohistochemistry images and quantification of POMC‐positive neurons in the ARC of vehicle‐ or leptin‐treated female *Ube3a* ^con^ and *Ube3a* ^NKO^ mice (*n* = 3–5 mice per group). All mice were female. 3 V, third ventricle; ARC, arcuate nucleus. Each dot represents one biologically independent mouse. Representative images are shown from biologically independent samples. Image quantification was performed using ImageJ. Scale bars, 100 µm. Data are presented as mean ± s.e.m. Statistical significance was determined using an unpaired two‐tailed Student's *t*‐test (d,e), one‐way analysis of variance (ANOVA) followed by Tukey's multiple‐comparisons test (f), or two‐way ANOVA followed by Tukey's multiple‐comparison test (h‐j). ^*^ *p* < 0.05, ^**^ *p* < 0.01, ^***^ *p *< 0.001, ^****^ *p *< 0.0001; n.s, not significant.

We next examined whether hypothalamic UBE3A expression responds to nutritional state. Hypothalamic UBE3A protein levels were markedly reduced in wild‐type (WT) mice fed a high‐fat diet (HFD), with a pronounced decrease in the ARC (Figure 1d,e). Acute changes in nutritional state also affected UBE3A abundance: fasting reduced hypothalamic UBE3A protein levels, whereas refeeding increased them relative to the fasted state, although they remained below those observed in the fed state (Figure 1f). Together, these findings identify UBE3A as a nutritionally responsive protein expressed in key hypothalamic neuronal populations and suggest a potential role in linking nutritional state to neuronal regulation of energy homeostasis.

### 2.2 Loss of Hypothalamic UBE3A Promotes Hyperphagia and Increased Adiposity in Female Mice Under Normal Chow Diet Conditions

To investigate the role of UBE3A in energy balance, we generated mice carrying an Nkx2.1‐Cre‐mediated deletion of the maternal floxed *Ube3a* allele (*Ube3a* ^m(f)/p(+)^; *Nkx2.1‐*Cre, hereafter referred to as *Ube3a* ^NKO^). Because the paternal *Ube3a* allele is predominantly silenced in neurons, deletion of the maternal floxed allele results in UBE3A loss in targeted neurons [7, 37].

Under normal chow diet (NCD) conditions, female *Ube3a* ^NKO^ mice exhibited progressive body weight gain accompanied by increased food intake (Figure S2a,b). To characterize the basis of the increased body weight, we assessed tissue weights and body composition. Liver and brown adipose tissue (BAT) weights did not differ significantly between genotypes (Figure S2c,d). In contrast, *Ube3a* ^NKO^ mice showed significantly increased white adipose tissue (WAT) mass (Figure S2e) and an elevated fat mass‐to‐lean mass ratio (Figure S2f), whereas absolute lean mass was unchanged (Figure S2g). These changes were accompanied by increased circulating leptin levels (Figure S2h), indicating that the increased body weight primarily reflected increased adiposity. Indirect calorimetry showed reduced oxygen consumption (VO_2_) and carbon dioxide production (VCO_2_) in *Ube3a* ^NKO^ mice, whereas the respiratory exchange ratio (RER) was unchanged (Figure S2i–k). Body‐weight‐normalized energy expenditure was significantly lower in *Ube3a* ^NKO^ mice than in controls over the 48‐h recording period (Figure S2l). Analysis of covariance (ANCOVA) using a common‐slope model also showed significantly lower mean absolute energy expenditure in *Ube3a* ^NKO^ mice after adjustment for lean mass (Figure S2m). In addition, *Ube3a* ^NKO^ mice exhibited lower core body temperature, whereas locomotor activity did not differ significantly between genotypes (Figure S2n,o). Together with increased food intake, these findings indicate disrupted energy homeostasis in female *Ube3a* ^NKO^ mice. Insulin tolerance tests (ITT) indicated impaired insulin sensitivity in *Ube3a* ^NKO^ mice, whereas glucose tolerance was comparable between genotypes (Figure S2p,q).

To determine whether the metabolic phenotype of *Ube3a* ^NKO^ mice was associated with impaired leptin responsiveness, we assessed the response to leptin in female mice. Mice received vehicle for 3 consecutive days, followed by vehicle or leptin for an additional 3 consecutive days (Figure 1g). In control mice, leptin administration reduced body weight and cumulative food intake, whereas these responses were markedly blunted in *Ube3a* ^NKO^ mice (Figure 1h,i). Consistent with this phenotype, *Ube3a* ^NKO^ mice exhibited reduced basal POMC expression in the ARC and an attenuated increase following leptin administration compared with controls (Figure 1j). These findings suggest that hypothalamic UBE3A loss is associated with impaired leptin responsiveness and an attenuated POMC expression response to leptin.

### 2.3 Nutrient Excess Reveals a Role for UBE3A in Metabolic Adaptation

We next examined whether nutritional stress reveals a role for UBE3A in the maintenance of energy homeostasis. Under HFD conditions, female *Ube3a* ^NKO^ mice developed more pronounced obesity than control mice, characterized by increased body weight, hyperphagia, and increased liver and adipose tissue weights (Figure 2a–e). Magnetic resonance imaging ‐based body composition analysis revealed increases in both fat mass and lean mass in *Ube3a* ^NKO^ mice, with a significantly higher fat mass‐to‐lean mass ratio (Figure 2f,g). These changes were accompanied by markedly elevated circulating leptin levels (Figure 2h). Histological analysis also revealed pronounced hepatic steatosis and adipocyte hypertrophy in both WAT and BAT (Figure 2i), indicating substantial tissue remodeling associated with HFD‐induced obesity.

**FIGURE 2:**
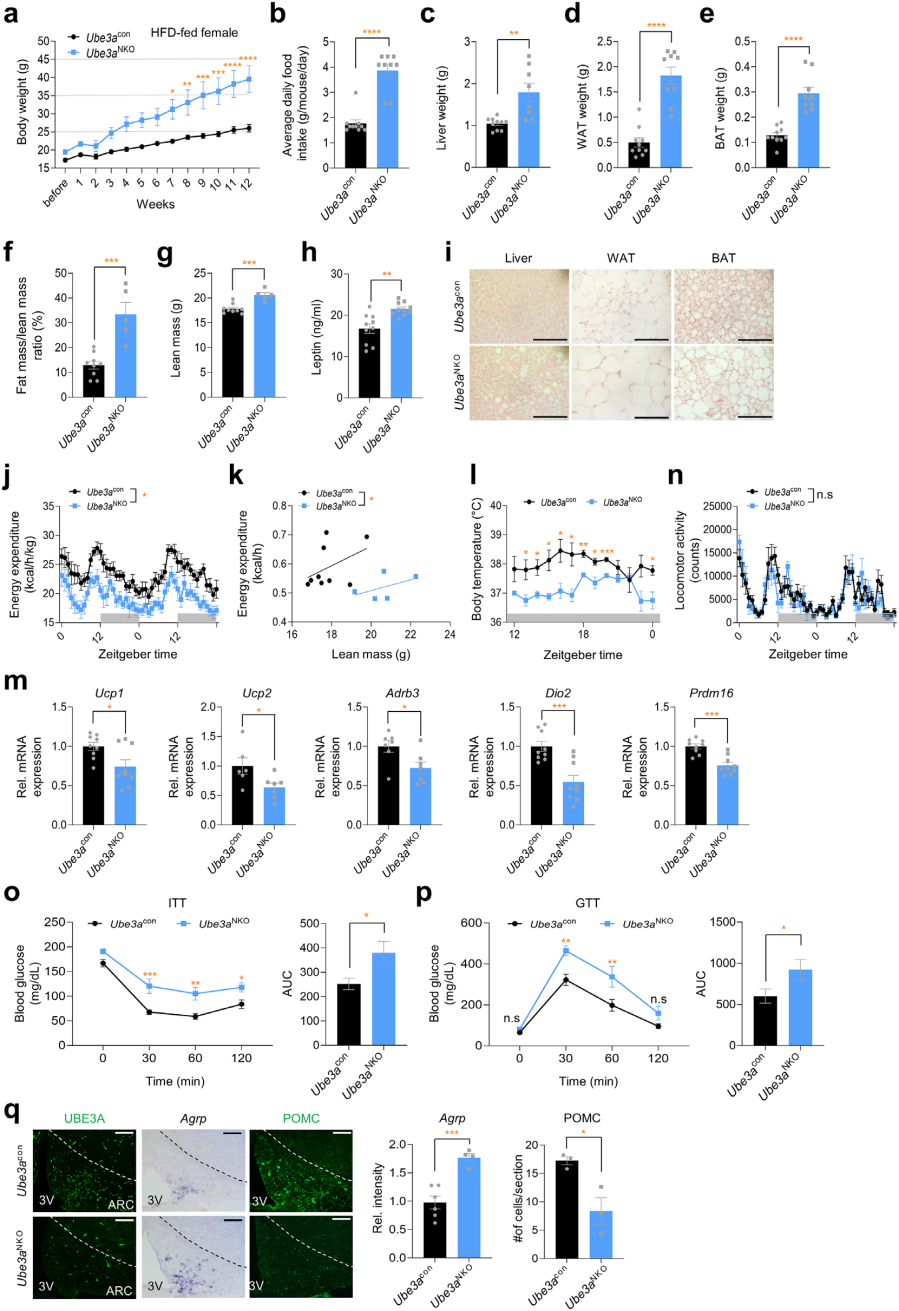
Hypothalamic *Ube3a* deficiency impairs metabolic adaptation to nutrient excess. (a) Body weight gain in female *Ube3a* ^con^ and *Ube3a* ^NKO^ mice fed an HFD for12 weeks starting at 8 weeks of age. (b) Average daily food intake per mouse during HFD feeding. (c‐e) Tissue weights of the liver (c), white adipose tissue (WAT) (d), and brown adipose tissue (BAT) (e) in HFD‐fed female *Ube3a* ^con^ and *Ube3a* ^NKO^ mice. For (a‐e), *Ube3a* ^con^ (*n* = 10 mice) and *Ube3a* ^NKO^ (*n* = 9 mice). (f,g) Fat mass‐to‐lean mass ratio (%) (f) and lean mass (g) in HFD‐fed mice. For (f,g), *Ube3a* ^con^ (*n* = 9 mice) and *Ube3a* ^NKO^ (*n* = 5 mice). (h) Serum leptin levels in HFD‐fed female *Ube3a* ^con^ and *Ube3a* ^NKO^ mice (*n* = 10 mice per group). (i) Representative hematoxylin and eosin (H&E)‐stained sections of liver, WAT and BAT from HFD‐fed mice. Scale bars, 100 µm. (j) Time course of body‐weight‐normalized energy expenditure (kcal/h/kg) over 48 h, measured by indirect calorimetry in HFD‐fed female mice. Gray bars indicate the dark phase. (k) Relationship between lean mass and mean absolute energy expenditure over the same 48‐h recording period. Absolute energy expenditure is expressed as kcal/h, without normalization to body weight or lean mass. Each point represents an individual mouse. ANCOVA was performed with mean absolute energy expenditure as the dependent variable, lean mass as a continuous covariate, and genotype as a categorical factor. The genotype × lean mass interaction was not statistically significant *(p* = 0.7010). In a common‐slope ANCOVA model excluding the interaction term, the genotype effect was significant after adjustment for lean mass (*p* = 0.0363). Lines represent separate linear regression fits for each genotype. For (j,k), *Ube3a* ^con^ (*n* = 9 mice) and *Ube3a* ^NKO^ (*n* = 5 mice). (l) Core body temperature monitored for 12 h under room‐temperature conditions (*n* = 4 mice per group). (m) Relative mRNA expression of thermogenic markers in BAT from HFD‐fed mice (*n* = 6–9 mice per group). Gene‐expression levels were normalized to *Ppia* (*Cyclophilin A)* and are presented relative to the control group, which was set to 1. (n) Locomotor activity measured over 48 h by indirect calorimetry in metabolic cages (*n* = 5–9 mice per group). (o,p) Insulin tolerance test (ITT) (o) and glucose tolerance test (GTT) (p) in HFD‐fed female *Ube3a* ^con^ and *Ube3a* ^NKO^ mice (*n* = 8–10 mice per group). Blood glucose levels were measured at the indicated time points, and the area under the curve (AUC) was quantified. (q) Representative images and quantification of expression in the ARC, *Agrp* expression assessed by in situ hybridization (ISH), and POMC‐positive neurons assessed by immunohistochemistry (IHC) in HFD‐fed female *Ube3a* ^con^ and *Ube3a* ^NKO^ mice (*n* = 3–6 mice per group). Bright‐field ISH signal intensity was normalized to the control group, which was set to 1. Scale bars, 50 µm. All mice were female. 3 V, third ventricle; ARC, arcuate nucleus. Each dot represents one biologically independent mouse. Representative images are shown from biologically independent samples. Image quantification was performed using ImageJ. Data are presented as mean ± s.e.m. Statistical significance was determined using two‐way ANOVA followed by Tukey's multiple‐comparisons test for (a,l), unpaired two‐tailed Student's *t*‐test (b‐h,m, and q), and two‐way repeated‐measures ANOVA with genotype and time as factors, including their interaction, with Geisser–Greenhouse correction (j,n). For (o,p), blood glucose time‐course data were analyzed by two‐way ANOVA followed by Tukey's multiple‐comparisons test, and AUC values were analyzed using unpaired two‐tailed Student's *t*‐tests. ^*^ *p* < 0.05, ^**^ *p* < 0.01, ^***^ *p *< 0.001, ^****^ *p *< 0.0001; n.s, not significant.

Metabolic phenotyping further revealed impaired adaptation to nutrient excess in *Ube3a* ^NKO^ mice. Body‐weight‐normalized energy expenditure was significantly lower in HFD‐fed female *Ube3a* ^NKO^ mice than in HFD‐fed controls over the 48‐h recording period (Figure 2j). ANCOVA using a common‐slope model also showed significantly lower mean absolute energy expenditure in *Ube3a* ^NKO^ mice after adjustment for lean mass (Figure 2k). These changes were accompanied by lower core body temperature (Figure 2l) and reduced expression of genes associated with thermogenesis in BAT, including *Ucp1*, *Ucp2*, *Adrb3*, *Dio2*, and *Prdm16* (Figure 2m). Locomotor activity did not differ significantly between genotypes (Figure 2n). Together with hyperphagia, these findings indicate disrupted energy homeostasis in HFD‐fed female *Ube3a* ^NKO^ mice. Consistent with these abnormalities, *Ube3a* ^NKO^ mice exhibited impaired insulin sensitivity and glucose tolerance under HFD conditions (Figure 2o,p). At the neuronal level, HFD‐fed *Ube3a* ^NKO^ mice exhibited increased *Agrp* expression and reduced POMC levels (Figure 2q), consistent with a shift toward orexigenic signaling. Comparable metabolic phenotypes were not detected in male *Ube3a* ^NKO^ mice under the same experimental conditions (Figures S3 and S4), suggesting that the metabolic effects of UBE3A loss may differ between sexes. Collectively, these findings show that hypothalamic UBE3A loss increases susceptibility to HFD‐induced obesity in female mice, accompanied by hyperphagia and reduced energy expenditure.

### 2.4 Loss of UBE3A in AgRP Neurons is Sufficient to Increase Susceptibility to HFD‐Induced Metabolic Dysfunction

Because hypothalamic *Ube3a* deletion produced obesity accompanied by increased orexigenic neuropeptide expression (Figure 2), we next examined whether UBE3A in AgRP neurons contributes to the regulation of energy balance. To address this question, we generated mice lacking maternal *Ube3a* selectively in AgRP neurons (*Ube3a* ^m(f)/p(+)^; *Agrp‐IRES‐*Cre, hereafter referred to as *Ube3a* ^AKO^). Female *Ube3a* ^AKO^ mice and littermate controls were fed an HFD for 8 weeks to determine whether AgRP neuron‐specific *Ube3a* loss was sufficient to reproduce key metabolic phenotypes observed in *Ube3a* ^NKO^ mice.

Under HFD conditions, female *Ube3a* ^AKO^ mice developed increased body weight and hyperphagia relative to littermate controls (Figure 3a,b). Liver, WAT, and BAT weights were also increased (Figure 3c–e). Body composition analysis revealed an increased fat mass‐to‐lean mass ratio, whereas absolute lean mass was not significantly altered (Figure 3f,g). Histological analysis further revealed hepatic steatosis and adipocyte hypertrophy in both WAT and BAT (Figure 3h), indicating substantial tissue remodeling following *Ube3a* loss in AgRP neurons. Indirect calorimetry showed reduced VO_2_ and VCO_2_ in *Ube3a* ^AKO^ mice, whereas the RER was unchanged (Figure 3i–k). Body‐weight‐normalized energy expenditure was significantly lower in HFD‐fed female *Ube3a* ^AKO^ mice than in HFD‐fed controls over the 48‐h recording period (Figure 3l). ANCOVA using a common‐slope model also showed significantly lower mean absolute energy expenditure in *Ube3a* ^AKO^ mice after adjustment for lean mass (Figure 3m). These changes were accompanied by lower core body temperature (Figure 3n) and reduced expression of thermogenesis‐related genes in BAT (Figure 3o), whereas locomotor activity did not differ significantly between genotypes (Figure 3p). The lower core body temperature and reduced expression of thermogenesis‐related genes are consistent with altered thermogenic regulation in *Ube3a* ^AKO^ mice. HFD‐fed *Ube3a* ^AKO^ mice also exhibited impaired insulin sensitivity and glucose tolerance (Figure 3q,r).

**FIGURE 3:**
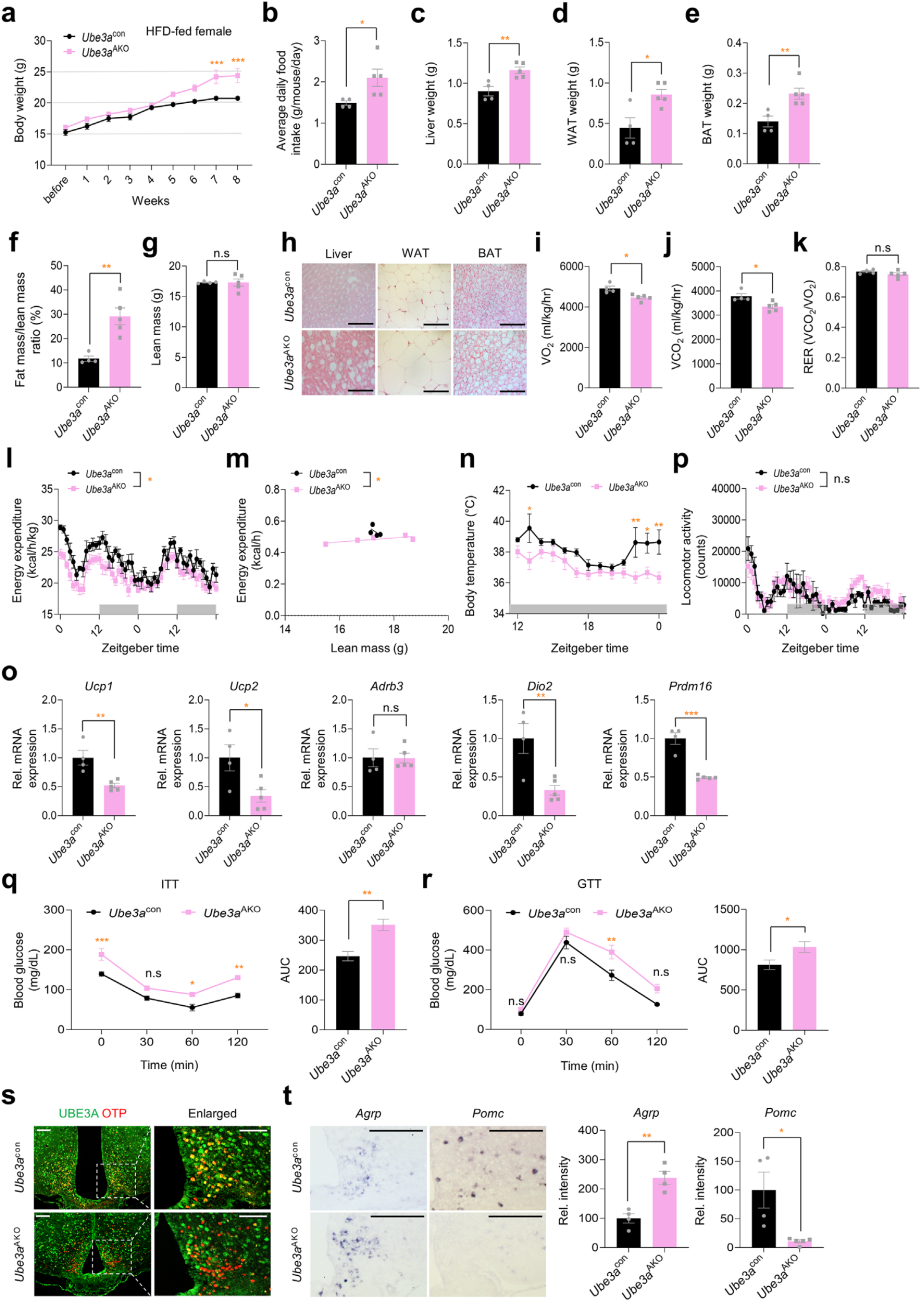
UBE3A function in AgRP neurons is sufficient to drive obesity and metabolic dysfunction during high‐fat diet feeding. (a) Body weight gain in female *Ube3a* ^con^ and *Ube3a* ^AKO^ mice fed an HFD for 8 weeks starting at 6 weeks of age. (b) Average daily food intake per mouse during HFD feeding. (c‐e) Tissue weights of the liver (c), WAT (d), and BAT (e) in HFD‐fed female *Ube3a* ^con^ and *Ube3a* ^AKO^ mice. (f,g) Fat mass‐to‐lean mass ratio (%) (f) and lean mass (g) in HFD‐fed mice. (h) Representative hematoxylin and eosin (H&E)‐stained sections of liver, WAT, and BAT from HFD‐fed mice. Scale bars, 100 µm. (i‐k) Oxygen consumption (VO_2_) (i), carbon dioxide production (VCO_2_) (j), and respiratory exchange ratio (RER) (k) in HFD‐fed female *Ube3a* ^con^ and *Ube3a* ^AKO^ mice. (l) Time course of body‐weight‐normalized energy expenditure (kcal/h/kg) over 48 h, measured by indirect calorimetry in HFD‐fed female mice. Gray bars indicate the dark phase. (m) Relationship between lean mass and mean absolute energy expenditure over the same 48‐h recording period. Absolute energy expenditure is expressed as kcal/h, without normalization to body weight or lean mass. Each point represents an individual mouse. ANCOVA was performed with mean absolute energy expenditure as the dependent variable, lean mass as a continuous covariate, and genotype as a categorical factor. The genotype × lean mass interaction was not significant *(p* = 0.2098). In a common‐slope ANCOVA model excluding the interaction term, the genotype effect was significant after adjustment for lean mass (*p* = 0.0292). Lines represent separate linear regression fits for each genotype. (n) Core body temperature monitored for 12 h under room‐temperature conditions. (o) Relative mRNA expression of thermogenic markers in BAT from HFD‐fed mice. Gene‐expression levels were normalized to *Ppia* and are presented relative to the control group, which was set to 1. (p) Locomotor activity measured over 48 h by indirect calorimetry in metabolic cages. (q,r) ITT (q) and GTT (r) in HFD‐fed female *Ube3a* ^con^ and *Ube3a* ^AKO^ mice. Blood glucose levels were measured at the indicated time points, and the AUC was quantified. For (a‐r), *Ube3a* ^con^ (*n* = 4 mice) and *Ube3a* ^AKO^ (*n* = 5 mice). (s) Representative IHC images showing reduced UBE3A expression in OTP‐positive AgRP neurons of HFD‐fed *Ube3a* ^AKO^ mice. Scale bars, 100 µm (t) Representative ISH images and quantification of *Agrp* and *Pomc* expression in the ARC of HFD‐fed *Ube3a* ^con^ and *Ube3a* ^AKO^ mice (*n* = 4–5 mice per group). Bright‐field ISH signal intensity was normalized to the control group, which was set to 100. Scale bars, 100 µm. All mice were female. Each dot represents one biologically independent mouse. Representative images are shown from biologically independent samples. Image quantification was performed using ImageJ. Data are presented as mean ± s.e.m. Statistical significance was determined using two‐way ANOVA followed by Tukey's multiple‐comparisons test (a,n), unpaired two‐tailed Student's *t*‐test (b‐g,i‐k,o, and t), and two‐way repeated‐measures ANOVA with genotype and time as factors, including their interaction, with Geisser–Greenhouse correction (l,p). For (q,r), blood glucose time‐course data were analyzed by two‐way ANOVA followed by Tukey's multiple‐comparisons test, and AUC values were analyzed using unpaired two‐tailed Student's *t*‐tests. ^*^ *p* < 0.05, ^**^ *p* < 0.01, ^***^ *p *< 0.001, ^****^ *p *< 0.0001; n.s, not significant.

In contrast to the phenotype observed following broader hypothalamic deletion, AgRP neuron‐specific *Ube3a* loss did not significantly affect body weight or metabolic parameters examined under NCD conditions (Figure S5). Male *Ube3a* ^AKO^ mice also showed no detectable metabolic abnormalities under either NCD (Figure S6) or HFD (Figure S7) conditions. These findings suggest that UBE3A function in AgRP neurons is particularly important in female mice during chronic nutrient excess. At the neuronal level, UBE3A immunoreactivity was almost completely absent from orthopedia homeobox (OTP)‐positive AgRP neurons in *Ube3a* ^AKO^ mice (Figure 3s). Consistent with the findings in *Ube3a* ^NKO^ mice, HFD‐fed *Ube3a* ^AKO^ mice exhibited increased hypothalamic *Agrp* expression and reduced *Pomc* expression (Figure 3t), suggesting a shift toward orexigenic signaling.

To test whether inhibition of AgRP neurons could restore POMC expression in *Ube3a* ^AKO^ mice, we used the inhibitory hM4Di designer receptors exclusively activated by designer drugs (DREADDs) (Figure S8a). All groups received clozapine‐*N*‐oxide (CNO): *Ube3a* ^con^ mice expressing mCherry, *Ube3a* ^AKO^ mice expressing mCherry, and *Ube3a* ^AKO^ mice expressing hM4Di‐mCherry. In this independent experiment, POMC expression was markedly reduced in CNO‐treated *Ube3a* ^AKO^ mice expressing mCherry compared with CNO‐treated *Ube3a* ^con^ mice expressing mCherry, recapitulating the reduction in POMC expression observed in the original *Ube3a* ^AKO^ cohort. In contrast, hM4Di‐mediated inhibition of AgRP neurons increased *Pomc* mRNA and POMC protein expression in *Ube3a* ^AKO^ mice to levels comparable to those of control mice (Figure S8b,c). In the paraventricular nucleus (PVN), AgRP immunoreactivity was reduced, whereas α‐MSH immunoreactivity was increased following hM4Di‐mediated inhibition of AgRP neurons (Figure S8d,e). These findings show that chemogenetic inhibition of AgRP neurons increases POMC expression and PVN α‐MSH immunoreactivity in *Ube3a* ^AKO^ mice, consistent with an improvement in the POMC‐melanocortin pathway. Collectively, these results show that loss of UBE3A in AgRP neurons is sufficient to increase susceptibility to HFD‐induced metabolic dysfunction in female mice. They further support AgRP neurons as an important cellular locus for UBE3A‐dependent regulation of energy homeostasis.

### 2.5 UBE3A Selectively Regulates the Hypothalamic H3K27 Methylation Through UTX Destabilization

Loss of *Ube3a* in hypothalamic cells caused metabolic dysfunction accompanied by increased *Agrp* expression and reduced *Pomc* expression (Figures 1, 2, 3), suggesting altered transcriptional regulation within arcuate neurons. Because UBE3A is a ubiquitin ligase with nuclear and chromatin‐associated functions, we hypothesized that UBE3A regulates metabolic homeostasis through epigenetic control of neuronal gene expression. Consistent with this possibility, HFD feeding has been reported to remodel hypothalamic histone methylation [38], implicating chromatin regulation in metabolic adaptation.

To determine whether UBE3A loss alters chromatin states, we profiled histone modifications in the hypothalamus of female *Ube3a* ^NKO^ mice. Immunoblot analysis revealed reduced histone H3 lysine 27 trimethylation (H3K27me3), whereas histone H3 lysine 4 mono‐, di‐, and trimethylation (H3K4me1, H3K4me2, and H3K4me3, respectively) were not significantly altered (Figure 4a). Immunofluorescence analysis further confirmed reduced H3K27me3 immunoreactivity in the ARC under both NCD and HFD conditions (Figure 4b). These findings suggest that UBE3A loss preferentially affects hypothalamic H3K27me3 among the histone modifications examined.

**FIGURE 4:**
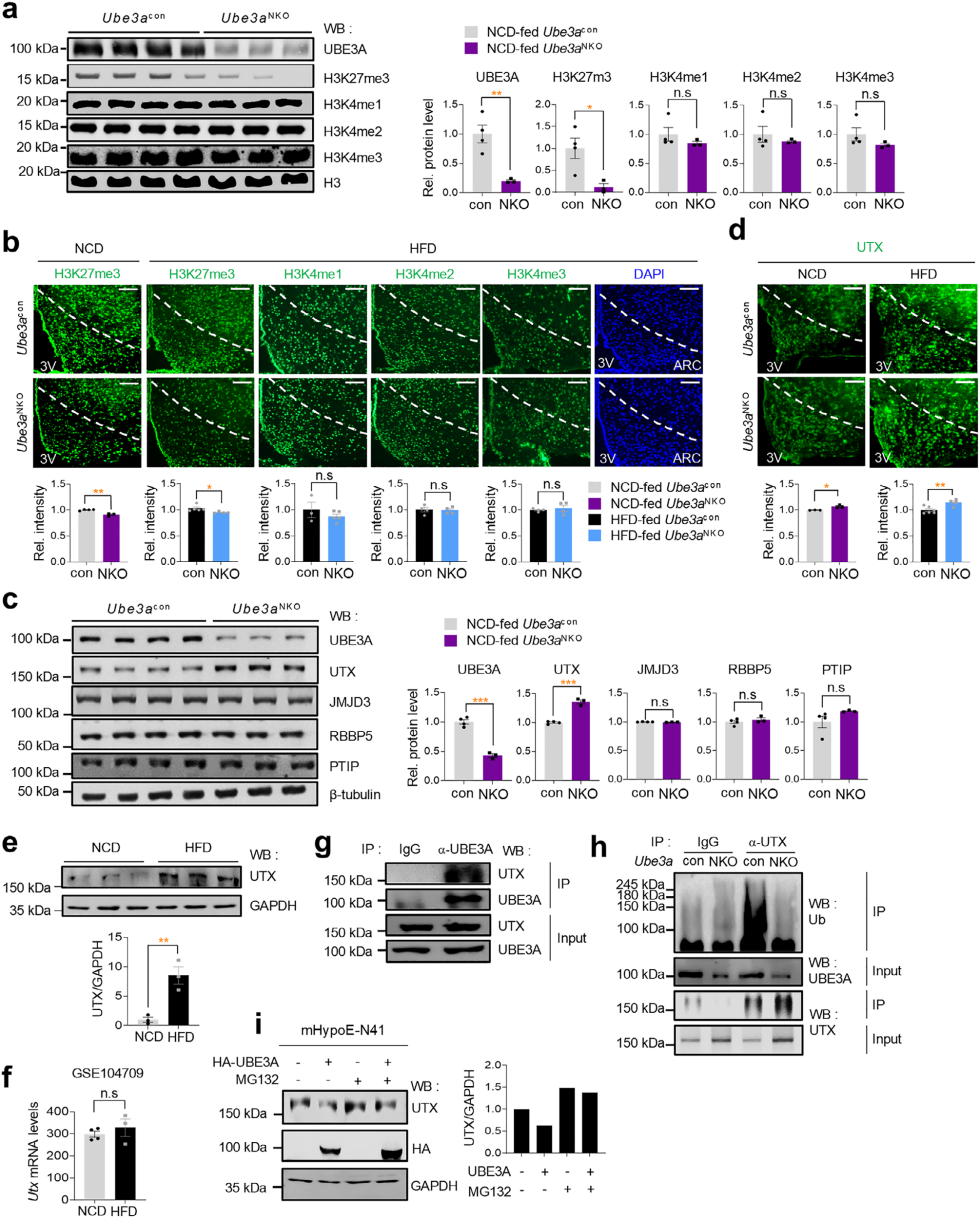
UBE3A regulates UTX protein stability and hypothalamic H3K27me3 levels. (a) Immunoblot analysis of hypothalamic lysates from 2‐month‐old female *Ube3a* ^con^ and *Ube3a* ^NKO^ mice maintained on an NCD, using antibodies against UBE3A, H3K27me3, H3K4me1, H3K4me2, H3K4me3, and total histone H3 (*n* = 4 and 3 mice per group, respectively). Band intensities were quantified using ImageJ and normalized to total histone H3. (b) Representative IHC images and quantification of H3K27me3 expression in the ARC of NCD‐fed female *Ube3a* ^con^ and *Ube3a* ^NKO^ mice (*n* = 4 and 3 mice per group, respectively), and of H3K27me3, H3K4me1, H3K4me2, and H3K4me3 expression in HFD‐fed female *Ube3a* ^con^ and *Ube3a* ^NKO^ mice (*n* = 3–5 mice per group). (c) Immunoblot analysis of hypothalamic lysates from NCD‐fed female *Ube3a* ^con^ and *Ube3a* ^NKO^ mice using antibodies against UBE3A, UTX, JMJD3, RBBP5, PTIP, and β‐tubulin (*n* = 4 and 3 mice per group, respectively). Band intensities were quantified using ImageJ and normalized to β‐tubulin. (d) Representative IHC images and quantification of UTX expression in the ARC of NCD‐ or HFD‐fed female *Ube3a* ^con^ and *Ube3a* ^NKO^ mice (*n* = 3–6 mice per group). (e) Immunoblot analysis and quantification of UTX protein levels in hypothalamic lysates from WT mice fed NCD or HFD for 12 weeks (*n* = 3 mice per group). Band intensities were quantified using ImageJ and normalized to GAPDH. (f) *Utx* mRNA levels in the hypothalamus of mice maintained on NCD or HFD, analyzed using a previously published RNA‐sequencing dataset (GSE104709; *n* = 4 and 3 biologically independent samples, respectively). (g) Co‐immunoprecipitation (CoIP) analysis of hypothalamic lysates from female WT mice showing an interaction between endogenous UBE3A and UTX. (h) Endogenous UTX ubiquitination in hypothalamic lysates from female *Ube3a* ^con^ and *Ube3a* ^NKO^ mice. Lysates were subjected to immunoprecipitation with an anti‐UTX antibody, followed by immunoblotting with an anti‐ubiquitin, anti‐UBE3A, and anti‐UTX antibodies. (i) mHypoE‐N41 hypothalamic neuronal cells overexpressing HA‐UBE3A were treated with the proteasome inhibitor MG132 (10 µM) for 8 h and immunoblotted using an anti‐UTX, anti‐HA, and anti‐GAPDH antibodies. Band intensities were quantified using ImageJ and normalized to GAPDH. Each dot represents one biologically independnet mouse. Representative IHC images are shown from biologically independent samples. Mean fluorescence intensity in the ARC was quantified using ImageJ and is expressed relative to the respective control group, which was set to 1. CoIP, endogenous ubiquitination, and MG132‐treatment experiments were independently repeated twice with similar results. Scale bars, 50 µm. Data are presented as mean ± s.e.m. Statistical significance was determined using unpaired two‐tailed Student's *t*‐test. ^*^ *p* < 0.05, ^**^ *p* < 0.01, ^***^ *p *< 0.001; n.s, not significant.

We next examined whether reduced H3K27me3 was associated with altered abundance of relevant histone methyltransferases or demethylases. In the liver, UBE3A has been shown to ubiquitinate the H3K4 methyltransferase mixed‐lineage leukemia 4 (MLL4; also known as KMT2D), providing precedent for UBE3A‐mediated regulation of chromatin modifiers [18]. However, proteomic analysis of brains of AS model mice revealed no significant changes in MLL4 or the H3K27 methyltransferase enhancer of zeste homolog 2 (EZH2) (Figure S9a) [39]. Although these data do not exclude changes in methyltransferase activity or hypothalamus‐specific regulation, they prompted us to examine alternative mechanisms. The H3K27 demethylase UTX (KDM6A), a component of the MLL4 complex that functionally opposes EZH2‐mediated H3K27 methylation, emerged as a candidate mediator.

Notably, UTX is encoded on the X chromosome and exhibits female‐biased expression, a feature that may be relevant to the metabolic phenotype observed in female *Ube3a* mutant mice. Immunoblot analysis revealed increased protein abundance of UTX in the hypothalamus of female *Ube3a* ^NKO^ mice, whereas the abundances of jumonji domain‐containing protein 3 (JMJD3; also known as KDM6B) and the MLL4‐complex components PAX transactivation domain‐interacting protein (PTIP) and retinoblastoma‐binding protein 5 (RBBP5) were not significantly altered (Figure 4c). Increased UTX expression was further observed in the ARC under both NCD and HFD conditions (Figure 4d). In contrast, male *Ube3a* ^NKO^ mice showed no significant changes in hypothalamic UTX protein abundance or H3K27me3 levels (Figure S9b). Because UBE3A was broadly expressed in AgRP, POMC, and GHRH neurons in both sexes (Figures 1a–c and S1a–c), the observed phenotypic differences are unlikely to be explained solely by differences in the neuronal distribution of UBE3A. Rather, these findings suggest that molecular responses to *Ube3a* loss may differ between sexes, potentially involving the UTX‐H3K27me3 axis.

To determine whether nutrient excess is associated with changes in this pathway, we analyzed WT mice after 12 weeks of HFD feeding. HFD increased hypothalamic UTX protein abundance while reducing UBE3A expression (Figures 1d and 4e). Analysis of published RNA‐sequencing (RNA‐seq) datasets showed minimal changes in *Utx* transcript abundance under HFD conditions (Figure 4f) [40], raising the possibility that increased UTX protein abundance involves post‐translational regulation. We therefore examined whether UBE3A regulates UTX stability through the ubiquitin‐proteasome system. Co‐immunoprecipitation (CoIP) from female hypothalamic tissue demonstrated an interaction between UBE3A and UTX (Figure 4g). In addition, endogenous UTX ubiquitination was reduced in hypothalamic tissue from *Ube3a* ^NKO^ mice relative to control mice (Figure 4h), supporting a role for UBE3A in endogenous UTX ubiquitination in the hypothalamus. To determine whether UBE3A‐mediated UTX turnover is proteasome‐dependent, mHypoE‐N41 hypothalamic neuronal cells were treated with the proteasome inhibitor MG132. MG132 treatment increased UTX protein levels and prevented the reduction in UTX levels induced by UBE3A overexpression (Figure 4i), indicating that UBE3A promotes proteasome‐dependent degradation of UTX in hypothalamic neuronal cells.

Moreover, substitution of a putative ubiquitination site in a PEST‐rich region of UTX (K867R) rendered UTX resistant to UBE3A‐mediated degradation, whereas the abundance of its Y‐linked homolog, UTY, was unaffected by UBE3A expression in mHypoE‐N41 cells (Figure S10a). We also performed a cycloheximide (CHX) chase assay in WT and *Ube3a*‐knockout (KO) mHypoE‐N41 cells expressing empty vector, HA‐UBE3A WT, or the catalytically inactive HA‐UBE3A C820A mutant. Notably, UTX protein levels were markedly higher in *Ube3a*‐KO cells expressing empty vector than in WT cells, consistent with increased UTX stability in the absence of UBE3A. Re‐expression of HA‐UBE3A WT in *Ube3a*‐KO cells reduced UTX protein levels and accelerated UTX turnover following CHX treatment, whereas the catalytically inactive HA‐UBE3A C820A mutant neither reduced UTX abundance nor accelerated its turnover (Figure S10b). Collectively, these findings indicate that UBE3A promotes UTX ubiquitination and proteasome‐dependent turnover in a catalytic activity‐dependent manner. Together, these findings establish UTX as a physiological substrate of UBE3A in hypothalamic neurons and link UBE3A‐dependent regulation of UTX stability to hypothalamic H3K27me3 levels.

### 2.6 Integrated Analyses Implicate UTX and ATF4 in ER Stress‐Responsive Transcription in the *Ube3a*‐Deficient Hypothalamus

To characterize transcriptional changes associated with hypothalamic UBE3A deficiency, we performed RNA sequencing (RNA‐seq) of ARC tissue from female *Ube3a* ^con^ and *Ube3a* ^NKO^ mice following 12 weeks of HFD feeding (GEO: GSE343626; Figure 5a). Differential expression analysis identified 290 differentially expressed genes, of which 270 were significantly upregulated in *Ube3a* ^NKO^ mice (Figure 5b). To identify upregulated genes associated with UTX occupancy, we integrated these RNA‐seq data with UTX CUT&Tag data generated from mouse hypothalamic tissue (GEO: GSE343655). Of the 270 upregulated genes, 26 were associated with UTX CUT&Tag peaks in annotated regulatory regions (Figure 5c). Functional enrichment analysis of these 26 genes revealed significant enrichment of ER stress‐related biological processes and the protein processing in the ER pathway (Figure 5d). Several ER stress‐associated genes, including *Pdia3*, *Pdia6*, *Dnajc3*, *Dnm1l*, and *Ubqln1*, were upregulated and associated with UTX CUT&Tag peaks (Figure 5e). Increased expression of these genes was independently validated in hypothalamic tissue from *Ube3a* ^NKO^ mice (Figure S11a). In addition, proteomic analysis of brains from AS model mice showed increased abundance of PDIA3, PDIA6, DNAJC3, DNM1L, and UBQLN1 (Figure S11b), consistent with increased abundance of the corresponding proteins in this model.

**FIGURE 5:**
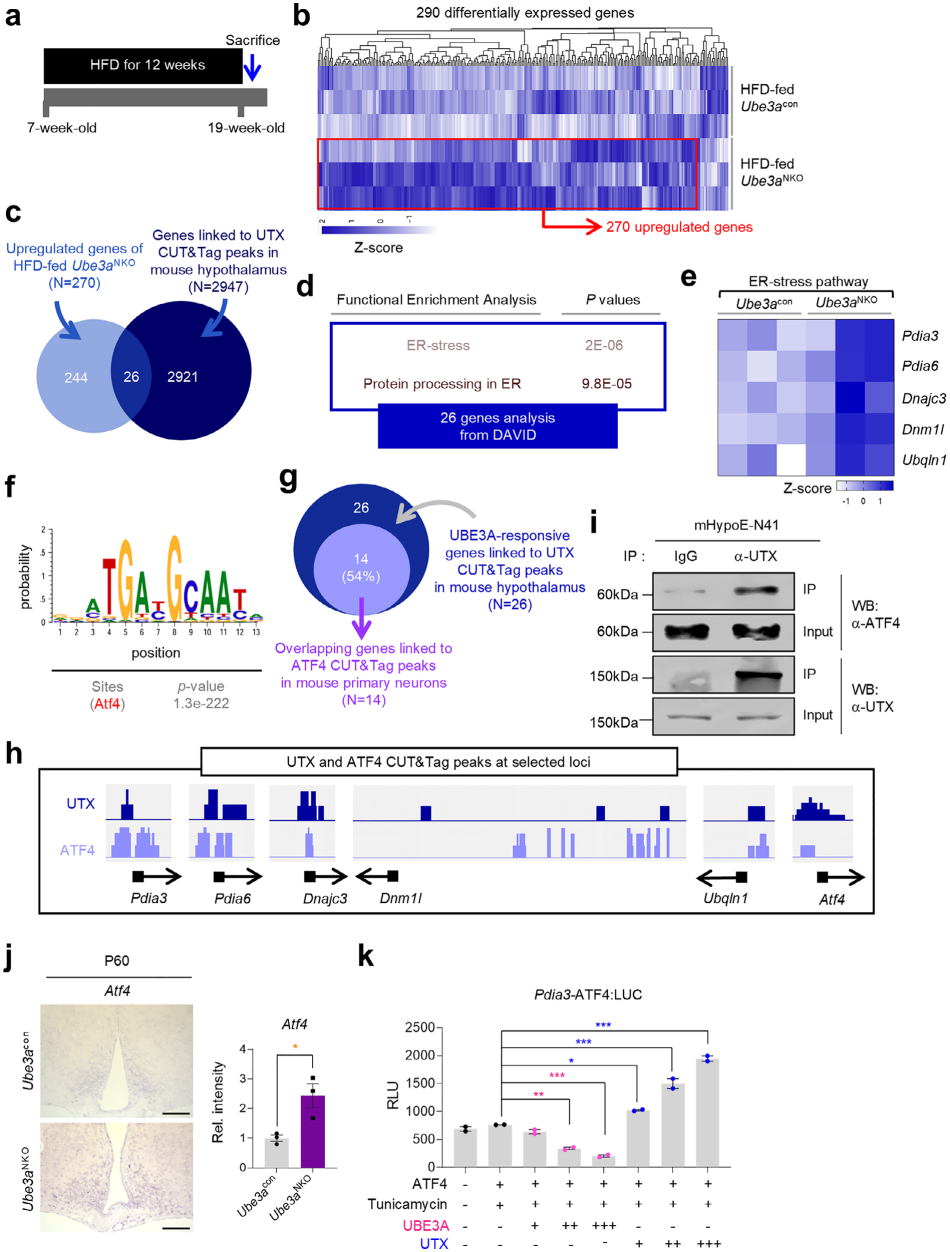
Integrated genomic and biochemical analyses implicate UTX and ATF4 in ER stress‐responsive transcription in the *Ube3a*‐deficient hypothalamus. (a) Schematic of the RNA‐sequencing (RNA‐seq) experimental design using ARC tissue from female *Ube3a* ^con^ and *Ube3a* ^NKO^ mice after 12 weeks of HFD feeding (*n* = 3 mice per group; GEO accession no. GSE343626). (b) Heatmap showing 270 significantly upregulated genes among the 290 differentially expressed genes identified in ARC tissue from HFD‐fed *Ube3a* ^NKO^ mice relative to HFD‐fed *Ube3a* ^con^ mice. (c) Venn diagram showing the overlap between the 270 upregulated genes identified by RNA‐seq and 2,947 genes linked to UTX CUT&Tag peaks in mouse hypothalamic tissue (GEO accession no. GSE343655), yielding 26 shared genes. (d) Gene Ontology (GO) analysis of biological processes enriched among the 26 shared genes identified in (c). (e) Heatmap showing the expression of selected ER stress‐associated genes upregulated in ARC tissue from HFD‐fed *Ube3a* ^NKO^ mice. (f) Motif enrichment analysis of hypothalamic UTX CUT&Tag peaks using Pscan‐ChIP, revealing significant enrichment of an ATF4 binding motif. (g) Gene‐level comparison of the 26 upregulated genes linked to hypothalamic UTX CUT&Tag peaks with genes linked to ATF4 CUT&Tag peaks in mouse primary neurons (GSE263464). Of these 26 genes, 14 (54%) were also associated with ATF4 peaks. (h) Representative UTX and ATF4 CUT&Tag tracks at selected ER stress‐associated loci and the *Atf4* locus. Arrows indicate the direction of transcription. The comparisons in (g) and (h) reflect associations across datasets generated from distinct neural preparations and do not establish UTX–ATF4 co‐occupancy in the same cells. (i) CoIP analysis showing an association between UTX and ATF4 in mHypoE‐N41 hypothalamic neuronal cells. (j) Representative ISH images and quantification of *Atf4* expression in the ARC of NCD‐fed female *Ube3a* ^con^ and *Ube3a* ^NKO^ mice at postnatal day 60 (P60; *n* = 3 mice per group). Bright‐field ISH signal intensity was quantified using ImageJ and is expressed relative to the control group, which was set to 1. Scale bars, 100 µm. Each dot represents one biologically independent mouse. (k) Luciferase reporter assay using a *Pdia3* regulatory region associated with UTX and ATF4 peaks in the respective CUT&Tag datasets (*Pdia3*‐ATF4:LUC). Reporter activity was measured under conditions of ATF4 expression and tunicamycin treatment, with increasing amounts of UBE3A or UTX expression plasmids, as indicated. Statistical comparisons were made against the ATF4‐expressing, tunicamycin‐treated condition without exogenous UBE3A or UTX. Two independent experiments were performed, with duplicate technical measurements per experiment. Technical replicates were averaged before statistical analysis, and each dot represents the mean from one independent experiment. Data are presented as mean ± s.e.m. Statistical significance was determined using an unpaired two‐tailed Student's *t*‐test (j) and one‐way ANOVA followed by Tukey's multiple‐comparisons test (k). ^*^ *p* < 0.05, ^**^ *p* < 0.01, ^***^ *p* < 0.001.

We next investigated transcription factors potentially associated with UTX‐bound regions. Motif enrichment analysis of hypothalamic UTX CUT&Tag peaks using Pscan‐ChIP [41] identified significant enrichment of an ATF4 binding motif near peak summits (Figure 5f). Furthermore, comparison with a publicly available ATF4 CUT&Tag dataset generated from mouse primary neurons (GSE263464) showed that 14 of the 26 upregulated genes associated with UTX peaks (54%) were also associated with ATF4 peaks (Figure 5g). Inspection of selected ER stress‐associated loci identified UTX peaks in hypothalamic tissue and ATF4 peaks in primary neurons within annotated promoter, enhancer, or 5′ untranslated regions of *Pdia3*, *Pdia6*, *Dnajc3*, *Dnm1l*, and *Ubqln1*, as well as at the *Atf4* locus (Figures 5h and S12a). Because the UTX and ATF4 datasets were generated from distinct neural preparations, these comparisons do not establish simultaneous occupancy of the same regulatory regions in the same cells. Rather, they identify shared gene associations across datasets that are consistent with a potential role for UTX and ATF4 in ER stress‐responsive transcription.

CoIP experiments in mHypoE‐N41 hypothalamic neuronal cells provided additional evidence of an association between UTX and ATF4 (Figure 5i), although this assay does not establish direct binding or their joint occupancy at specific genomic loci. ISH in the ARC and IHC analysis in OTP‐positive AgRP neurons further showed increased ATF4 expression in *Ube3a* ^NKO^ mice (Figures 5j and S12b). To assess the activity of a *Pdia3* regulatory region associated with UTX and ATF4 peaks in the respective CUT&Tag datasets, we generated a luciferase reporter containing this region (*Pdia3*‐ATF4:LUC). Under conditions of ATF4 expression and tunicamycin treatment, increasing UBE3A expression progressively reduced reporter activity, whereas increasing UTX expression enhanced reporter activity (Figure 5k). These findings show opposing effects of UBE3A and UTX on reporter activity under the tested conditions but do not establish ATF4 dependence or demonstrate that UBE3A suppresses reporter activity through UTX degradation.

### 2.7 UBE3A Deficiency is Associated With Increased UTX Enrichment and Reduced H3K27me3 at Selected ER Stress‐Responsive Loci

To investigate the association of UTX and ATF4 with ER stress‐responsive genes, we examined their respective CUT&Tag peaks in regulatory regions near the transcription start sites of *Pdia3, Pdia6, Dnajc3, Dnm1l*, and *Ubqln1* (Figures 5h and S12a). Separate chromatin immunoprecipitation (ChIP) assays in mHypoE‐N41 hypothalamic neuronal cells showed that tunicamycin‐induced ER stress increased the enrichment of both ATF4 and UTX at these regulatory regions (Figure 6a,b). These findings support stress‐dependent association of each factor with the selected ER stress‐responsive loci. Because UTX promotes histone H3 lysine 27 demethylation, we next examined whether UBE3A affects the chromatin state at these regions. *Ube3a* knockdown reduced H3K27me3 enrichment at the ATF4‐associated regions of *Dnajc3, Dnm1l*, and *Ubqln1* under both basal and tunicamycin‐treated conditions (Figure 6c). These results indicate that UBE3A contributes to the maintenance of repressive H3K27me3 at selected ER stress‐responsive loci in hypothalamic neuronal cells.

**FIGURE 6:**
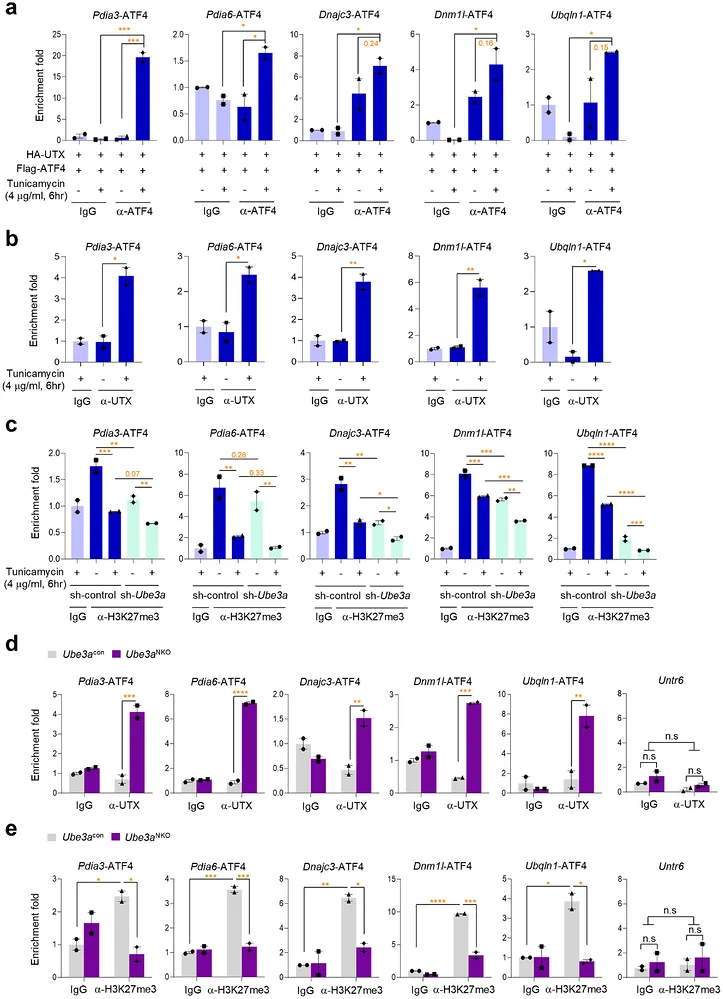
UBE3A loss alters UTX enrichment and H3K27me3 levels at selected ER stress‐responsive loci. (a) Chromatin immunoprecipitation followed by quantitative PCR (ChIP‐qPCR) analysis of ATF4 enrichment at ATF4‐binding regions associated with *Pdia3*, *Pdia6*, *Dnajc3*, *Dnm1l*, and *Ubqln1* in mHypoE‐N41 hypothalamic neuronal cells coexpressing HA‐UTX and Flag‐ATF4. (b) ChIP‐qPCR analysis of UTX enrichment at the same ATF4‐associated regions in mHypoE‐N41 cells. (c) ChIP‐qPCR analysis of H3K27me3 enrichment at the ATF4‐associated regions of *Pdia3*, *Pdia6*, *Dnajc3*, *Dnm1l*, and *Ubqln1* in sh‐control and sh‐*Ube3a* mHypoE‐N41 cells under basal and tunicamycin‐treated conditions. (d,e) ChIP‐qPCR analysis of UTX (d) and H3K27me3 (e) enrichment at ATF4‐associated regions of *Pdia3*, *Pdia6*, *Dnajc3*, *Dnm1l*, and *Ubqln1* in hypothalamic tissue from NCD‐fed female *Ube3a* ^con^ and *Ube3a* ^NKO^ mice. The *Untr6* genomic region was used as a negative control in ChIP assays. ChIP enrichment values are expressed relative to the corresponding reference condition, which was set to 1. ChIP‐qPCR experiments were independently repeated twice, with duplicate technical measurements per experiment. Technical replicates were averaged before statistical analysis, and each dot represents the mean value from one biologically independent experiment. Data are presented as mean ± s.e.m. Statistical significance was determined using one‐way ANOVA followed by Tukey's multiple‐comparisons test (a,b) or two‐way ANOVA followed by Tukey's multiple‐comparisons test (c‐e). ^*^ *p* < 0.05, ^**^ *p* < 0.01, ^***^ *p *< 0.001, ^****^ *p *< 0.0001; n.s, not significant.

We next examined whether similar chromatin changes occurred in vivo using hypothalamic tissue from female *Ube3a* ^con^ and *Ube3a* ^NKO^ mice. ChIP analysis revealed increased UTX enrichment at regulatory regions associated with *Pdia3, Pdia6, Dnajc3, Dnm1l*, and *Ubqln1* in *Ube3a* ^NKO^ mice relative to controls (Figure 6d). H3K27me3 enrichment at these regions was also markedly reduced in *Ube3a* ^NKO^ mice (Figure 6e). In contrast, neither UTX nor H3K27me3 enrichment differed significantly between genotypes at the negative control *Untr6* region (Figure 6d,e), consistent with region‐specific rather than uniform changes across all loci examined. Together, these findings show that UBE3A deficiency is associated with increased UTX enrichment and reduced H3K27me3 at selected ER stress‐responsive loci in the hypothalamus. These changes are consistent with a chromatin state permissive for transcription and support a potential role for UBE3A‐dependent regulation of UTX in ER stress‐responsive gene expression.

### 2.8 Systemic GSK‐J4 Treatment Improves Metabolic Outcomes and Increases Hypothalamic H3K27me3 Levels in *Ube3a*‐Deficient Mice

Given that UBE3A loss is associated with UTX accumulation and reduced H3K27me3, we next examined whether treatment with a KDM6‐family demethylase inhibitor could ameliorate metabolic abnormalities in *Ube3a* ^NKO^ mice. Female *Ube3a* ^NKO^ mice and littermate controls were fed an HFD and treated daily with GSK‐J4 for 4 weeks (Figure 7a). GSK‐J4 treatment attenuated body‐weight gain and reduced food intake in HFD‐fed *Ube3a* ^NKO^ mice (Figure 7b–d). GSK‐J4 did not significantly alter liver weight but reduced WAT and BAT weights (Figure 7e). Histological analyses also showed improvements in hepatic steatosis and adipocyte hypertrophy following GSK‐J4 treatment (Figure 7f).

**FIGURE 7:**
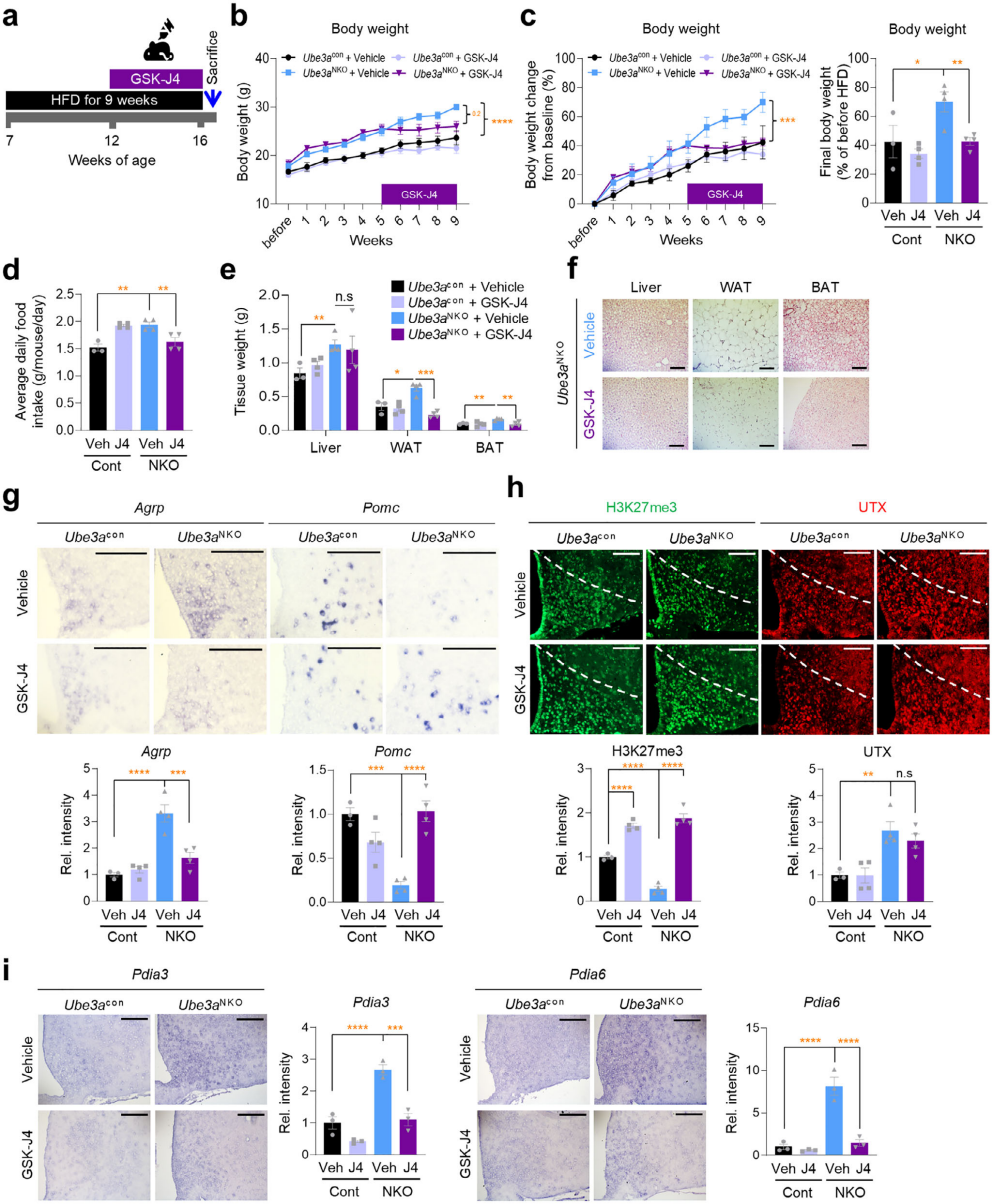
Systemic GSK‐J4 treatment attenuates metabolic abnormalities in *Ube3a*‐deficient mice. (a) Experimental scheme for GSK‐J4 treatment. Female *Ube3a* ^con^ and *Ube3a* ^NKO^ mice were started on an HFD at 7 weeks of age and maintained on the diet throughout the experiment. Beginning 5 weeks after HFD initiation, mice received daily vehicle or GSK‐J4 treatment for 4 weeks, followed by tissue collection at 16 weeks of age (*n* = 3–4 mice per group). (b,c) Body weight (b) and percentage change in body weight relative to the start of HFD feeding (c) in vehicle‐ or GSK‐J4‐treated female *Ube3a* ^con^ and *Ube3a* ^NKO^ mice. (d) Average daily food intake (g per mouse per day) in vehicle‐ or GSK‐J4‐treated HFD‐fed mice. (e) Weights of the liver, WAT, and BAT. (f‐i) Representative H&E‐stained sections of liver, WAT and BAT(f) ISH images and quantification of *Agrp* and *Pomc* expression (g) IHC images and quantification of H3K27me3 and UTX expression (h) ISH images and quantification of *Pdia3* and *Pdia6* expression (i) in vehicle‐ or GSK‐J4‐treated HFD‐fed *Ube3a* ^con^ and *Ube3a* ^NKO^ mice. Representative images are shown from biologically independent samples. For image‐based quantification in (g–i), multiple images were acquired from each mouse, and the average value was calculated for each mouse. Image quantification was performed using ImageJ. Each dot represents one biologically independent mouse. Scale bars, 100 µm. Data are presented as mean ± s.e.m. Statistical significance was determined using two‐way ANOVA followed by Tukey's multiple‐comparisons test. ^*^ *p* < 0.05, ^**^ *p* < 0.01, ^***^ *p *< 0.001, ^****^ *p *< 0.0001; n.s, not significant.

To determine whether these effects were accompanied by changes in hypothalamic neuropeptide expression, we examined the ARC. Vehicle‐treated *Ube3a* ^NKO^ mice exhibited increased *Agrp* expression and reduced *Pomc* expression, both of which shifted toward control levels following GSK‐J4 treatment (Figure 7g). We next assessed hypothalamic H3K27me3 levels and ER stress‐associated gene expression. Immunofluorescence analysis showed that GSK‐J4 treatment increased H3K27me3 levels in *Ube3a* ^NKO^ mice without significantly altering UTX protein abundance (Figure 7h). This pattern is consistent with inhibition of demethylase activity rather than a reduction in UTX abundance. In addition, the elevated expression of the ER stress‐associated genes *Pdia3* and *Pdia6* in HFD‐fed *Ube3a* ^NKO^ mice was reduced following GSK‐J4 treatment (Figure 7i). Collectively, these findings show that systemic GSK‐J4 treatment improves metabolic and molecular abnormalities in *Ube3a* ^NKO^ mice, accompanied by increased hypothalamic H3K27me3 levels. However, because GSK‐J4 was administered systemically and inhibits multiple KDM6‐family demethylases, these results do not establish that the observed effects are mediated specifically by inhibition of hypothalamic UTX/KDM6A. In addition, the treatment‐associated reduction in food intake may contribute to the improvements in body weight and other metabolic outcomes.

## 3 Discussion

This study identifies a proteostasis‐associated epigenetic process that may contribute to hypothalamic metabolic adaptation. Hypothalamic *Ube3a* deficiency increased susceptibility to obesity, particularly in female mice, and was associated with hyperphagia, impaired leptin responsiveness, reduced energy expenditure, lower core body temperature, and impaired glucose metabolism. Our data support a model in which UBE3A limits UTX abundance through ubiquitin‐dependent proteasomal turnover, potentially contributing to the maintenance of H3K27me3 at selected ER stress‐responsive loci. Loss of UBE3A is associated with UTX accumulation, reduced H3K27me3, and increased expression of ER stress‐associated genes. Together, these observations suggest a link between protein homeostasis, chromatin regulation, and systemic energy balance (Figure 8).

**FIGURE 8:**
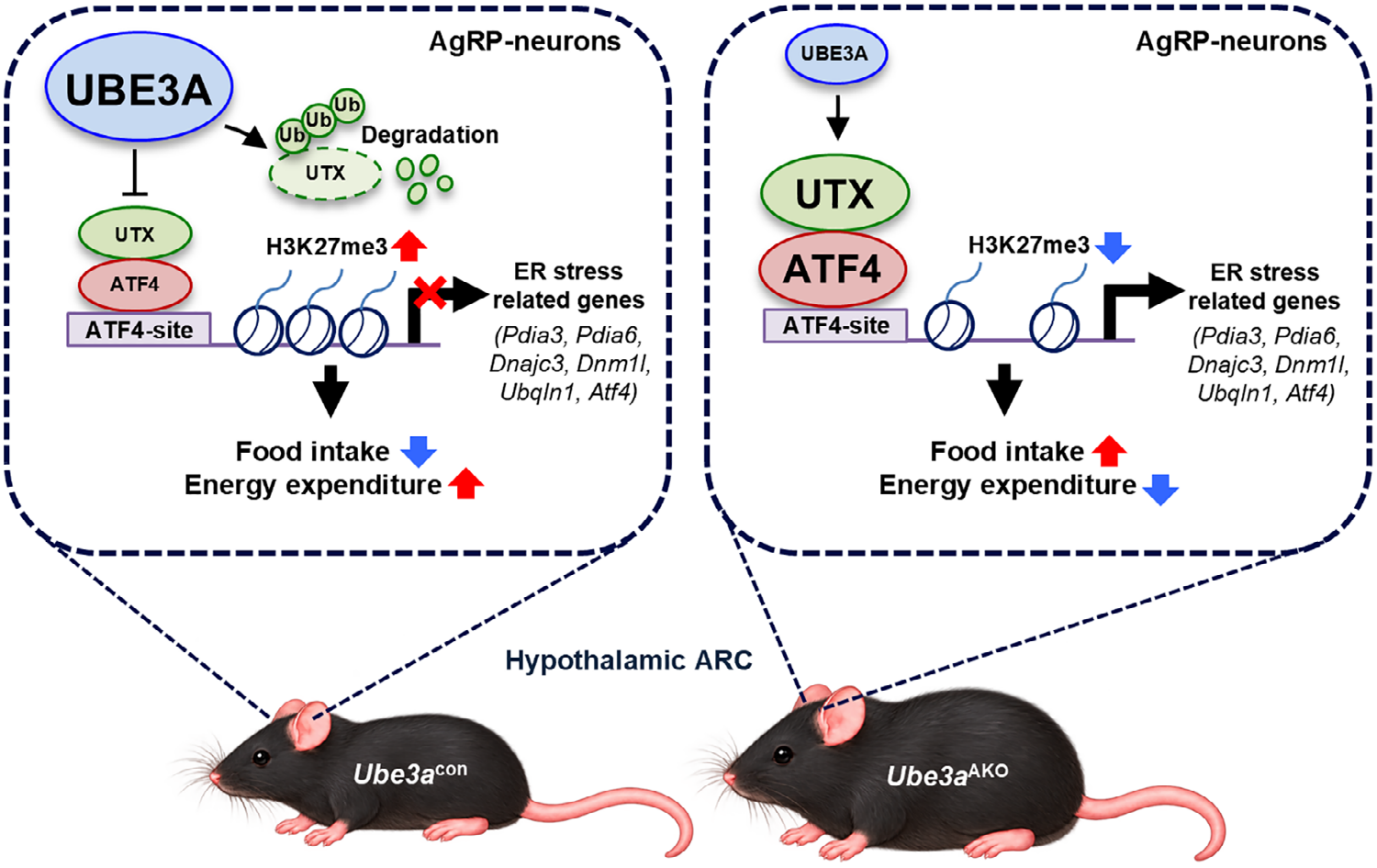
Proposed model of UBE3A‐dependent regulation of UTX stability and ER stress‐responsive transcription in the hypothalamus.

Proposed model illustrating that hypothalamic UBE3A promotes ubiquitination and proteasome‐dependent turnover of UTX, thereby contributing to the maintenance of H3K27me3 at selected ER stress‐responsive loci. Loss of UBE3A in hypothalamic neurons leads to UTX accumulation and reduced H3K27me3 and is associated with increased expression of ER stress‐responsive genes, potentially involving ATF4‐linked transcriptional regulation. These molecular changes accompany hyperphagia, reduced energy expenditure, and increased susceptibility to HFD‐induced obesity.

UBE3A has previously been implicated in the regulation of chromatin‐associated proteins, including the H3K4 methyltransferase MLL4/KMT2D [18]. Our findings extend this role by supporting UTX/KDM6A, an H3K27 demethylase, as a physiological substrate of UBE3A in hypothalamic neurons. The reduction in H3K27me3, without significant changes in the H3K4 methylation marks examined, together with increased UTX protein abundance in *Ube3a*‐deficient hypothalamus, suggests preferential alteration of H3K27me3 among the histone modifications assessed. In contrast, JMJD3/KDM6B abundance and the abundance of the MLL4‐complex components examined were not significantly altered. Consistent with these findings, prior proteomic analyses of AS model brains showed no detectable changes in MLL4 or EZH2 abundance [39], although these observations do not exclude changes in methyltransferase activity or hypothalamus‐specific regulation. The reciprocal changes in UBE3A and UTX protein abundance following HFD feeding, despite minimal changes in *Utx* transcript levels [40], are consistent with post‐translational regulation of UTX abundance during nutrient excess. Together with the ubiquitination, proteasome‐inhibition, and protein‐turnover experiments, these findings support a role for UBE3A in controlling UTX stability.

ER stress in hypothalamic neurons is closely linked to obesity and leptin resistance. Chronic overnutrition activates the unfolded protein response and can impair leptin and insulin signaling through induction of negative regulators and inflammatory pathways [19, 20, 21, 22, 23, 24]. ER stress can also disrupt neuropeptide processing and melanocortin signaling [25]. In this context, the enrichment of ER stress‐related genes among the transcriptional changes associated with *Ube3a* loss suggests that UBE3A‐dependent regulation of UTX may influence how hypothalamic neurons respond to nutritional stress. Consistent with previous evidence that HFD can alter hypothalamic histone methylation [38] and that tunicamycin‐induced ER stress reduces hypothalamic H3K27me3 [42], our findings suggest that reduced H3K27me3 may provide a chromatin environment permissive for increased expression of ER stress‐responsive genes. Whether this transcriptional response is adaptive or contributes to metabolic dysfunction remains to be determined.

Our data further suggest a potential functional link between UTX and ATF4 in the regulation of ER stress‐responsive genes. ATF4 acts as a central regulator of the integrated stress response and has been implicated in metabolic stress signaling, including the regulation of *Pdia3* transcription [43]. ATF4 is also an important regulator of energy homeostasis in the ARC, where its activity in POMC and AgRP neurons modulates fat mass, at least in part, by regulating energy expenditure [44, 45]. The association between UTX and ATF4 detected by CoIP, enrichment of ATF4 motifs within UTX‐bound regions, and stress‐associated enrichment of each factor at selected loci are consistent with potential cooperation in ER stress‐responsive transcription. However, the comparison of genome‐wide UTX and ATF4 CUT&Tag datasets identifies shared peak‐associated genes, rather than demonstrating overlapping peaks at identical genomic coordinates. These datasets are also derived from distinct neural preparations, precluding conclusions about co‐occupancy in the same cells. Although separate ChIP assays in mHypoE‐N41 cells show enrichment of UTX and ATF4 at selected regulatory regions, they do not establish simultaneous occupancy of the same DNA molecules. Thus, functional cooperation between UTX and ATF4 at endogenous loci remains to be established. Genetic epistasis experiments, including cell type‐specific manipulation of *Utx/Kdm6a* or *Atf4* in AgRP neurons, are needed to determine whether either factor is required for the metabolic phenotype associated with *Ube3a* deficiency and to clarify their functional relationship within the proposed pathway.

The phenotypic differences between *Ube3a* ^NKO^ and *Ube3a* ^AKO^ mice highlight the importance of cell type and nutritional context. Whereas broader hypothalamic *Ube3a* deletion caused metabolic abnormalities under NCD conditions, AgRP neuron‐specific deletion primarily increased vulnerability to HFD‐induced obesity, identifying AgRP neurons as a key cellular locus for UBE3A‐dependent metabolic regulation. This difference may reflect the broader recombination pattern of *Nkx2.1*‐Cre, which affects multiple hypothalamic and forebrain cell types and can also target tanycytes [46]. The increase in POMC expression following chemogenetic inhibition of AgRP neurons further supports a role for AgRP neuronal regulation in the POMC‐related changes observed in *Ube3a* ^AKO^ mice, although it does not establish increased AgRP neuronal activity following *Ube3a* loss. Moreover, AgRP neurons can regulate feeding through both melanocortin‐dependent and ‐independent pathways [47, 48]. POMC‐pathway changes may therefore contribute to the metabolic phenotype, but their causal and relative contributions remain to be determined.

An additional finding is the difference in metabolic responses between female and male mice. Metabolic abnormalities were observed predominantly in females, despite broadly similar UBE3A distribution across the examined hypothalamic neuronal populations in both sexes. Moreover, male *Ube3a* ^NKO^ mice showed no significant changes in UTX abundance or H3K27me3 levels, suggesting that the observed phenotypic differences are unlikely to be explained solely by neuronal UBE3A distribution. The X‐linked nature of UTX, the limited demethylase activity of its Y‐linked homolog UTY, and estrogenic modulation of leptin sensitivity and ER stress responses may contribute to these differences [49, 50, 51]. These observations suggest that molecular responses downstream of *Ube3a* loss may differ between sexes, although the underlying mechanisms remain to be established.

Analysis of covariance (ANCOVA) using a common‐slope model with lean mass as a covariate showed significantly lower mean absolute energy expenditure over the 48‐h recording period in HFD‐fed female *Ube3a* ^NKO^ and *Ube3a* ^AKO^ mice than in their respective controls. These findings were accompanied by reduced oxygen consumption, lower core body temperature, and reduced expression of thermogenesis‐related genes in brown adipose tissue, without a significant difference in locomotor activity. Nevertheless, pair‐feeding or food‐intake‐matched studies will be needed to help distinguish the contributions of increased food intake from those of altered energy expenditure to the obesity phenotype.

Systemic GSK‐J4 treatment increased hypothalamic H3K27me3 levels and improved molecular and metabolic abnormalities in *Ube3a*‐deficient mice. These findings are consistent with a potential role for KDM6‐associated chromatin regulation but do not establish that increased hypothalamic H3K27me3 mediates the metabolic improvements. They should not be interpreted as evidence for a UTX‐specific or hypothalamus‐specific rescue mechanism. GSK‐J4 inhibits multiple KDM6‐family demethylases, including JMJD3/KDM6B, and was administered systemically; thus, effects mediated by other KDM6‐family members, peripheral tissues, or additional targets cannot be excluded. In addition, the treatment‐associated reduction in food intake may have contributed to the improved metabolic outcomes. Although systemic GSK‐J4 has been reported to increase hypothalamic H3K27me3 [42], future studies combining intracerebroventricular or ARC‐targeted delivery, food‐intake‐matched controls, and UTX‐selective genetic approaches will be needed to clarify the anatomical site and molecular targets responsible for the observed effects and to define the contribution of UTX to energy homeostasis.

In conclusion, our findings support a model in which UBE3A‐dependent proteostatic control of UTX contributes to H3K27me3 maintenance and ER stress‐responsive transcriptional regulation in hypothalamic neurons. This model extends the framework for metabolic regulation beyond hormonal and circuit‐level control by linking ubiquitin‐dependent protein turnover to chromatin regulation. Further genetic and cell type‐specific studies are required to establish whether UTX–ATF4‐associated transcriptional changes causally contribute to the metabolic phenotype and whether selective modulation of this pathway can improve metabolic homeostasis under chronic nutrient excess.

## 4 Experimental Section

### 4.1 Mouse Models and Ethics

*Ube3a* ^m(f)/p(+)^ were generated at the University of North Carolina Animal Models Core facility by using the *Ube3a*‐knockout first targeting construct (CSD46841; clone PG00126_Z_3_B08A1), which was engineered by the trans‐NIH Knockout Mouse Project (KOMP; www.komp.org) [52]. *Nkx2.1‐*Cre (The Jackson Laboratory, strain #008661) [53] and *AgRP‐IRES‐*Cre (The Jackson Laboratory, strain #012899) [54] mouse models were applied in this study. Female *Ube3a* ^m(f)/p(+)^ mice were crossed with male *Nkx2.1‐*Cre or *AgRP‐IRES‐*Cre mice to generate *Ube3a* ^m(f)/p(+)^; *Nkx2.1‐*Cre mice (*Ube3a* ^NKO^ mice) and *Ube3a* ^m(f)/p(+)^; *AgRP‐IRES‐*Cre mice (*Ube3a* ^AKO^ mice). All mice were bred and maintained under specific pathogen‐free conditions on a normal 12‐h dark and 12‐h light cycle with ad libitum access to food and water and controlled temperature and humidity. Animal studies were authorized by Seoul National University's Institutional Animal Care and Use Committee (IACUC) and carried out in accordance with established guidelines (IACUC permit number: SNU‐231016‐2‐3). Detailed information for mouse genotyping primers is provided in Table S1.

To obtain diet‐induced obesity, 2‐month‐old mice were fed HFD (60% kcal fat, D12492, Research Diets Inc.) for 8 or 12 weeks. Age‐matched control mice were maintained on a normal chow diet (NCD; standard rodent pellet diet) and served as lean controls. Body weight was measured weekly throughout the experimental period. For 24‐h fasting experiments, food was withdrawn at the onset of the light cycle (10:00 AM), while water was provided ad libitum, to ensure circadian consistency.

### 4.2 Glucose and Insulin Tolerance Tests (GTT and ITT)

For glucose tolerance tests (GTTs), mice were fasted for 16 h and then intraperitoneally injected with D‐glucose (2.5 g/kg body weight; dissolved in distilled water). For insulin tolerance tests (ITTs), mice were fasted for 4 h and subsequently intraperitoneally injected with recombinant human insulin (0.75 U/kg body weight; diluted in saline). Blood glucose levels were measured from tail blood using a glucometer (Accu‐Chek, Roche Applied Science) at baseline and at 30, 60, and 120 min after injection.

### 4.3 Indirect Calorimetry and Body Composition Measurements

Energy expenditure and respiratory exchange ratio (RER) were assessed using an indirect calorimetry system (TSE LabMaster system, Germany) at the Korea Metabolic Phenotyping Center (KMPC). Mice were individually housed in metabolic chambers and acclimated for 48 h before data collection. Metabolic parameters were recorded at 1‐h intervals over a 48‐h recording period. Energy expenditure and RER were calculated from oxygen consumption (VO_2_) and carbon dioxide production (VCO_2_). Body‐weight‐normalized energy expenditure and locomotor activity were analyzed over time using two‐way repeated‐measures ANOVA, with genotype as the between‐subject factor and time as the within‐subject factor, including the genotype × time interaction. Geisser–Greenhouse correction was applied to account for potential violations of sphericity. For analysis of covariance (ANCOVA), mean absolute energy expenditure (kcal/h) over the 48‐h recording period was calculated for each mouse without normalization to body weight or lean mass. ANCOVA was performed using GraphPad Prism 11, with mean absolute energy expenditure as the dependent variable, lean mass as a continuous covariate, and experimental group as a categorical factor. The genotype × lean mass interaction was tested to assess whether regression slopes differed between groups. When the interaction was not statistically significant, the interaction term was omitted, and lean mass‐adjusted group differences were assessed using a common‐slope ANCOVA model by comparing regression elevations, as previously described [55]. Body composition was measured using a Minispec LF50 body composition analyzer (Bruker, Germany) at the KMPC.

### 4.4 Body Temperature Measurement

For body temperature monitoring, an IPTT‐300 transponder was subcutaneously implanted into the dorsal region of each mouse. Mice were allowed to recover for at least 1 week before temperature measurements. Body temperature was subsequently recorded at 1‐h intervals over a 12‐h period using a wireless XPT/IPTT reader (DAS‐8027‐IUS).

### 4.5 Leptin Responsiveness Assay

Seven‐week‐old female mice were intraperitoneally injected with either 0.9% saline or leptin (2 mg/kg body weight, R&D Systems, 498‐OB‐05 M) once daily for 3 consecutive days immediately before the onset of the dark cycle. Body weight and food intake were monitored daily throughout the treatment period.

### 4.6 RNA Isolation and Quantitative RT‐PCR (qRT‐PCR)

Total RNA was extracted from brown adipose tissue (BAT) using TRIzol reagent (Invitrogen), followed by reverse transcription into cDNA using an RT‐PCR kit (210212, Qiagen). Quantitative RT‐PCR was carried out with SYBR Green qPCR Premix (RT500U, Enzynomics) using a Bio‐Rad real‐time PCR detection system. Relative transcript abundance was calculated using the comparative Ct method, with *Ppia* (*Cyclophilin A*) as the reference gene. Each analysis was performed using biologically independent samples, and data are presented as mean ± s.e.m. Primer sequences are provided in Table S1.

### 4.7 GSK‐J4 Administration Experiment

Pharmacological inhibition of KDM6‐family demethylase activity was achieved by daily intraperitoneal administration of GSK‐J4 at 30 mg/kg body weight once daily at 11:00 a.m. GSK‐J4 was prepared at 3 mg/mL in H_2_O containing 2% (v/v) DMSO. Female mice were started on an HFD at 7 weeks of age and maintained on the HFD throughout the experiment. Beginning at 12 weeks of age, mice received daily intraperitoneal injections of GSK‐J4 or vehicle for 4 consecutive weeks while remaining on the HFD.

### 4.8 Immunohistochemistry (IHC)

Brains containing the hypothalamus were prefixed in paraformaldehyde for 24 h and sequentially incubated in 5% sucrose for 1 h, 10% sucrose for 1 h, 15% sucrose for 4 h, and 30% sucrose for 24 h at 4°C. Tissues were then embedded in optimal cutting temperature (OCT) compound, frozen, and stored at −80°C. Coronal brain sections (12 µm) were prepared using a cryostat (Leica, France). Immunohistochemistry was performed as previously described [56]. Briefly, sections were incubated overnight at 4°C with primary antibodies against UBE3A, POMC, OTP, H3K27me3, H3K4me1, H3K4me2, H3K4me3, UTX, and ATF4 diluted in blocking buffer. The following day, sections were washed with phosphate‐buffered saline containing Tween 20 (PBST), incubated with secondary antibodies conjugated with fluorescence, then washed and counterstained with DAPI. Detailed information on the antibodies used is provided in Table S2.

### 4.9 In Situ Hybridization (ISH)

For ISH, coronal brain sections (18 µm) were prepared using a cryostat (Leica, France). ISH was performed as previously described [56]. Briefly, digoxigenin (DIG)‐labeled RNA probes were hybridized to brain sections overnight at 65°C. Following hybridization, sections were sequentially washed in 5x SSC buffer for 10 min and twice in 0.2x SSC buffer for 30 min each at 65°C. Sections were then incubated for 1 h at room temperature in blocking buffer containing 1 M Tris‐HCl (pH 7.5), 5 M NaCl, and 4% BSA, followed by overnight incubation at 4°C with anti‐DIG‐alkaline phosphatase (AP) antibody (11093274910, Roche, 1:5000 dilution) in blocking buffer containing 2% BSA. Hybridization signals were visualized using an NBT/BCIP substrate solution (S3771, Promega), and sections were mounted with Permount mounting medium.

### 4.10 Hematoxylin and Eosin (H&E) Staining

Liver, white adipose tissue, and brown adipose tissue samples were fixed in 10% formalin and embedded in paraffin. Paraffin‐embedded tissues were sectioned at 5 µm thickness using a microtome (Leica, France). After deparaffinization in xylene, sections were stained with hematoxylin and eosin according to a standard protocol [57].

### 4.11 Plasmids

6xMyc‐tagged human UTX gene was cloned into a pCS2 vector for mammalian expression (Plasmid #17438, Addgene). 6xMyc‐tagged human UTX K867R and UTY genes were cloned into a pCS2 vector for mmalian expression. Flag‐tagged mosue ATF4 gene was cloned into a pcDNA3 vector for mammalian expression. HA‐tagged human UBE3A and UBE3A C820A were cloned into a pcDNA3 vector for mammalian expression. For generation of the *Pdia3*‐ATF4 reporter construct, a genomic region of the mouse *Pdia3* locus containing a putative ATF4‐binding site was amplified by PCR using the Table S1 primers. The amplified fragment was cloned into the TK‐Luc firefly luciferase reporter vector using the SalI and BamHI restriction sites. The resulting *Pdia3*‐ATF4 reporter construct was used to assess transcriptional activity of the ATF4‐responsive region of *Pdia3*. For knockdown of *Ube3a* in mHypoE‐N41 cells, short hairpin RNA (shRNA) constructs targeting mouse *Ube3a* were generated using the EFU6‐300 vector, which contains GFP to monitor transfected cells. The complementary oligonucleotides used to generate the *Ube3a*‐trageting shRNA were provided in Table S1. The oligonucleotides were annealed and cloned into the EFU6‐300 vector.

### 4.12 Cell Culture and Luciferase Assay

All cells were maintained at 37°C in a humidified chamber with 5% CO_2_. HEK293T cells (HB‐8065, ATCC), a human embryonic kidney cell line, and mHypoE‐N41 cells (CLU121, Biocompare), a mouse embryonic hypothalamic cell line, were cultured in high‐glucose DMEM (L0103, Biowest) supplemented with 10% heat‐inactivated fetal bovine serum (FBS) and 1% penicillin/streptomycin. For luciferase reporter assays, HEK293T cells were seeded in 48‐well plates and cultured for 24 h before transient transfection using SuperFect reagent (301305, Qiagen). Luciferase activity was measured 48 h after transfection. A β‐galactosidase expression plasmid was co‐transfected as an internal control to normalize transfection efficiency. Luciferase assays were independently repeated at least twice, and data are presented as relative luciferase units (mean ± s.e.m).

### 4.13 Generation of *Ube3a*‐Knockout (KO) Cell Line

A CRISPR/Cas9‐mediated lentiviral approach was used to generate a *Ube3a*‐KO mHypoE‐N41 cell line. Guide RNAs targeting mouse *Ube3a* were designed using the Synthego CRISPR design tool, and selected guide RNA sequences were cloned into the lentiCRISPRv2 vector (#52961; Addgene, MA) using Esp3I restriction sites. Lentiviral particles were produced in HEK293T‐LentiX cells by co‐transfection of the lentiCRISPRv2‐based construct with the packaging plasmids psPAX2 and pMD2.G. mHypoE‐N41 cells were transduced with the resulting lentivirus and selected with puromycin. Single cells were manually picked and expanded to establish individual clones. Candidate clones were screened for loss of UBE3A protein expression by immunoblotting. The guide RNA sequences used for *Ube3a* gene editing are provided in Table S1.

### 4.14 Western Blotting and Co‐Immunoprecipitation (CoIP)

mHypoE‐N41 cells were transfected with expression vectors encoding 6×Myc‐tagged UTX WT, UTX K867R, or UTY, or HA‐tagged UBE3A, as indicated. For the MG132 treatment assay, cells transfected with the HA‐UBE3A expression vector were treated with MG132 (10 µM; 474790, Sigma) for 8 h at 72 h after transfection. Transfections were performed using Lipofectamine 3000 (Invitrogen). Transfected mHypoE‐N41 cells or mouse hypothalamic tissues were lysed in tissue lysis buffer containing 150 mM NaCl, 1 mM EDTA, 20 mM Tris‐HCl (pH 8.0), 0.5% NP‐40, 2 mM PMSF, 10% glycerol, and Na_3_VO_4_ followed by incubation on ice. Lysates were centrifuged at 13 000 × g for 3 min at 4°C, and the cleared supernatants were collected for immediate use or stored at −80°C. Protein concentrations were determined using the Bradford assay.

Protein samples were mixed with 1x SDS sample buffer, boiled for 5 min at 100°C, separated by SDS‐PAGE using appropriate acrylamide gel concentrations, and transferred onto nitrocellulose membranes (10600001, GE Healthcare Life Science). Membranes were blocked with 10% skim milk (232100, BD Difco) for 1 h and incubated overnight at 4°C with primary antibodies against UBE3A, UTX, GAPDH, H3K27me3, H3K4me1, H3K4me2, H3K4me3, H3, JMJD3, RBbP5, PTIP, Myc, HA, and β‐tubulin. Detailed antibody information is provided in Table S2. Membranes were subsequently incubated with appropriate HRP‐conjugated secondary antibodies, and immunoreactive bands were visualized using ECL substrate (BNROW‐0008, NICSROWEST) and detected using x‐ray film or an ImageQuant 800 system.

For CoIP assays, mHypoE‐N41 cells or mouse hypothalamic tissues were lysed in IP buffer containing 150 mM NaCl, 1 mM EDTA, 20 mM Tris‐HCl (pH 8.0), 0.5% NP‐40, 2 mM PMSF, 10% glycerol, and Na_3_VO_4_. Following centrifugation at 13,000 x g for 3 min at 4°C, cleared lysates were incubated with the indicated antibodies (rabbit IgG, UTX, or UBE3A antibodies) for 2 h at 4°C, followed by overnight incubation with protein A‐agarose beads (15918014, Thermo Fisher Scientific). Immunoprecipitated proteins were eluted by boiling the beads in 1x SDS sample buffer for 5 min at 100°C, separated by SDS‐PAGE, and transferred to membranes. Membranes were then incubated overnight at 4°C with primary antibodies against UBE3A, UTX, ATF4, or Ub, followed by HRP‐conjugated secondary antibodies and ECL detection as described above.

### 4.15 Cycloheximide Chase Assay

mHypoE‐N41 WT and *Ube3a*‐KO cells were seeded in six well plates and cultured to approximately 70–80% confluence. Cells were transiently transfected using the Lipofectamine3000 (Invitrogen) with plasmids encoding 6×Myc‐UTX together with either HA‐UBE3A WT, HA‐UBE3A C820A mutant, or empty vector control. At 72 h after transfection, protein synthesis was inhibited by treatment with cycloheximide (CHX; final concentration, 200 ng/mL). Cells were harvested at 0, 3, 6 h after CHX treatment by washing once with ice‐cold PBS and lysing in cell lysis buffer containing 150 mM NaCl, 1 mM EDTA, 20 mM Tris‐HCl (pH 8.0), 0.5% NP‐40, 2 mM PMSF, 10% glycerol, and Na_3_VO_4_. Lysates were centrifuged at 13 000 × g for 3 min at 4°C, and the cleared supernatants were collected for immediate western blot analysis or stored at −80°C until use.

### 4.16 RNA‐Seq Experiments and Data Analysis

Seven‐week‐old female mice were fed an HFD for 12 weeks. Hypothalamic tissues were dissected, and total RNA was extracted using TRIzol reagent (Invitrogen). RNA integrity was assessed using a Bioanalyzer. RNA‐seq libraries were prepared according to the TruSeq Stranded mRNA Sample Preparation Guide (Part #15031047 Rev. E), and library quality was assessed by Bioanalyzer analysis and real‐time RT‐PCR. Libraries were sequenced on an Illumina NovaSeq6000 platform. Raw sequencing reads in FASTQ format were processed using Partek Flow software (Partek, St. Louis, MO, USA). Low‐quality and short reads were removed, and the remaining reads were aligned to the mouse reference genome (mm10). HISAT2 and STAR were evaluated for read alignment, and HISAT2 was used for the final analysis based on its higher overall alignment rate. Post‐alignment quality control was performed to assess mapping performance. Aligned reads were annotated and quantified using the RefSeq Transcripts release 93 annotation model, and transcript abundance was normalized as fragments per kilobase of transcript per million mapped reads (FPKM).

Differential expression analysis was performed using the Gene‐Specific Analysis (GSA) framework implemented in Partek Flow, with experimental group specified as the categorical factor. The primary comparison relevant to the present study was HFD‐*Ube3a* ^NKO^ versus HFD‐ *Ube3a* ^con^. Multiple testing was controlled using the Benjamini–Hochberg false discovery rate (FDR) step‐up procedure, and FDR‐adjusted *p*‐values < 0.05 were considered statistically significant.

Functional annotation and upstream regulator analyses were performed using Ingenuity Pathway Analysis (IPA) and the Database for Annotation, Visualization, and Integrated Discovery (DAVID) [58]. Raw sequencing and processed expression data have been deposited in the NCBI Gene Expression Omnibus (GEO) under accession number GSE343626.

### 4.17 Chromatin Immunoprecipitation (ChIP)

ChIP assays using mHypoE‐N41 cells or mouse hypothalamic tissues were performed as previously described [59]. For cell‐based experiments, mHypoE‐N41 cells were treated with tunicamycin (4 µg/ml) or vehicle control for 6 h before sample collection. For tissue‐based ChIP assays, hypothalamic tissues were isolated from female NCD‐fed *Ube3a* ^con^ and *Ube3a* ^NKO^ mice at postnatal day 50 (P50). The following antibodies were used for ChIP assays: control IgG, anti‐UTX, anti‐ATF4, and anti‐H3K27me3 antibodies (see Table S2). Primer sequences used for ChIP‐PCR analyses are listed in Table S1.

### 4.18 CUT&Tag Sequencing

Eight‐week‐old female wild‐type mice were administered tunicamycin (2.5 mg/kg) for 6 h. Hypothalamic tissue was then rapidly dissected and homogenized in a sucrose‐based nuclei‐ isolation buffer. Nuclei were purified by centrifugation through a sucrose cushion and used for CUT&Tag library preparation.

CUT&Tag libraries were prepared according to the manufacturer's instructions using the TruePrep Index Kit V2 for Illumina (#TD202, Vazyme) and an anti‐UTX antibody (Millipore). Libraries were sequenced on an Illumina NovaSeq 6000 platform to generate paired‐end 101‐bp reads. Raw sequencing reads were assessed for quality using FastQC. Adapter sequences and low‐quality reads were removed using Trimmomatic. Filtered reads were aligned to the mouse reference genome (mm10) using Bowtie2, and PCR duplicates were removed using Picard. Peaks were called using SEACR (v1.3) in stringent mode with a threshold of 0.01. ENCODE blacklist regions were subsequently removed using BEDTools. Peak annotation was performed using ChIPseeker with RefSeq gene annotations. Coverage analysis and quality‐control assessments, including gene‐body coverage and fraction of reads in peaks (FRiP) analysis, were performed using deepTools.

For comparative analysis and visualization, publicly available CUT&Tag datasets were retrieved from the Gene Expression Omnibus (GEO) database. The ATF4 CUT&Tag dataset generated from mouse primary neurons (GSE263464) was analyzed [60]. Motif enrichment analysis was performed using PscanChIP, and genomic tracks were visualized using the Integrated Genomics Viewer (IGV).

The raw UTX CUT&Tag sequencing data generated from mouse hypothalamic tissue have been deposited in GEO under accession number GSE343655.

### 4.19 Designer Receptors Exclusively Activated by Designer Drugs (DREADD) Experiment

To assess whether altered POMC signaling in mice with AgRP neuron‐specific *Ube3a* deficiency is associated with AgRP neuronal activity, AgRP neurons were chemogenetically inhibited using an inhibitory hM4Di‐DREADD system. The hM4Di coding sequence was fused in‐frame upstream of the *mCherry* sequence and cloned into a Cre‐dependent, double‐floxed inverted open reading frame (DIO) vector driven by the human synapsin (hSyn) promoter, generating AAV‐hSyn‐DIO‐hM4Di‐mCherry (Cat#. 26‐A126). A Cre‐dependent AAV‐hSyn‐DIO‐mCherry vector (Cat#. 26‐A113) was used as a control. Viral constructs were generated, verified, and packaged by the Korea Institute of Science and Technology (KIST). *Ube3a* ^con^ mice received ARC injection of the Cre‐dependent mCherry control virus (AAV‐hSyn‐DIO‐mCherry). *Ube3a* ^AKO^ mice received injections of either the mCherry control virus or the hM4Di‐mCherry virus (AAV‐hSyn‐DIO‐hM4di‐mCherry). Thus, three experimental groups were included.

For stereotaxic injections into the ARC, mice were anesthetized and placed in a stereotaxic apparatus. A single 2 µL injection of virus (8.1^*^10^12^ GC/ml) was delivered into the ARC at the following coordinates relative to bregma: anteroposterior (AP), −1.8 mm; mediolateral (ML), 0 mm; and dorsoventral (DV), −5.8 mm. Mice were allowed to recover for 3 weeks to permit viral expression. All experimental groups subsequently received clozapine‐*N*‐oxide (CNO; 3 mg/kg, i.p. injection) and were perfused 3 h after CNO administration. Brains were collected for analysis of *Pomc* mRNA and POMC protein expression in the ARC as well as AgRP and α‐MSH immunoreactivity in the paraventricular nucleus (PVN). Viral expression and injection sites were confirmed by mCherry fluorescence.

### 4.20 Quantification and Statistical Analysis

Data are presented as mean ± s.e.m. Data transformation or normalization was performed only where indicated in the corresponding figure legends. No data points were excluded as outliers. Sample sizes (n) represent biologically independent animals or independent experiments, as specified in the corresponding figure legends. Statistical analyses were performed using GraphPad Prism (version 11.0.0). Comparisons between two groups were performed using unpaired two‐tailed Student's *t*‐test. Comparisons among multiple groups were performed using one‐way or two‐way analysis of variance (ANOVA), as appropriate, followed by Tukey's multiple‐comparisons test. Time‐course energy expenditure and locomotor activity data were analyzed using two‐way repeated‐measures ANOVA, with genotype as the between‐subject factor and time as the within‐subject factor, including the genotype × time interaction. Geisser–Greenhouse correction was applied to account for potential violations of sphericity. Analysis of covariance (ANCOVA) was used to compare mean absolute energy expenditure (kcal/h) between genotypes after adjustment for lean mass. For this analysis, the mean over the 48‐h recording period was calculated for each mouse without normalization to body weight or lean mass. Lean mass was included as a continuous covariate and genotype as a categorical factor. The genotype × lean mass interaction was first tested to assess differences in regression slopes. When the interaction was not statistically significant, it was omitted from the model, and the lean mass‐adjusted genotype effect was evaluated using a common‐slope ANCOVA model. Differences were considered statistically significant at an alpha level of 0.05. Exact sample sizes, statistical tests, and significance thresholds for individual experiments are provided in the corresponding figure legends.

## Author Contributions

J.L. and S.L. conceived the study, designed the experiments, and wrote the manuscript. J.L., J.Y., Y.A.K., Z.W., and Y.L. performed experiments and data analysis. W.‐J.S. analyzed RNA‐seq data, and J.L. analyzed CUT&Tag sequencing data. J.K.S. interpreted the metabolic‐cage data. S.L. supervised the project, interpreted the data, and revised the manuscript. All authors critically read and approved the final version of the manuscript.

## Funding

This research was supported by the National Research Foundation of Korea (NRF) grant funded by the Korea government (MSIT) (RS‐2024‐00453874) to S. Lee. Korea Mouse Phenotyping Project (2013M3A9D5072550) of the Ministry of Science, ICT and Future Planning through the National Research Foundation to J.K. Seong.

## Conflicts of Interest

The authors declare no conflicts of interest.

## Supporting information




**Supporting File 1**: advs78567‐sup‐0001‐SuppMat.docx.

**Supporting File 2**: advs78567‐sup‐0002‐SuppMat.zip.

## Data Availability

The data that support the findings of this study are available in the supplementary material of this article.

## References

[1] J. P. Thaler , C.‐X. Yi , E. A. Schur , et al., “Obesity is Associated With Hypothalamic Injury in Rodents and Humans,” Journal of Clinical Investigation 122, no. 1 (2012): 153–162, 10.1172/JCI59660. PMC3248304 22201683

[2] J. W. Hill , J. K. Elmquist , and C. F. Elias , “Hypothalamic Pathways Linking Energy Balance and Reproduction,” American Journal of Physiology‐Endocrinology and Metabolism 294, no. 5 (2008): E827–E832, 10.1152/ajpendo.00670.2007. PMC5724360 18285524

[3] K. Timper and J. C. Bruning , “Hypothalamic Circuits Regulating Appetite and Energy Homeostasis: Pathways to Obesity,” Disease Models & Mechanisms 10, no. 6 (2017): 679–689, 10.1242/dmm.026609. PMC5483000 28592656

[4] J. N. Campbell , E. Z. Macosko , H. Fenselau , et al., “A Molecular Census of Arcuate Hypothalamus and Median Eminence Cell Types,” Nature Neuroscience 20, no. 3 (2017): 484–496, 10.1038/nn.4495. PMC5323293 28166221

[5] M. Scheffner , J. M. Huibregtse , R. D. Vierstra , and P. M. Howley , “The Hpv‐16 E6 and E6‐Ap Complex Functions as a Ubiquitin‐Protein Ligase in the Ubiquitination of P53,” Cell 75, no. 3 (1993): 495–505, 10.1016/0092-8674(93)90384-3. 8221889

[6] C. Sailer , F. Offensperger , A. Julier , et al., “Structural Dynamics of the E6ap/Ube3a‐E6‐P53 Enzyme‐Substrate Complex,” Nature Communications 9, no. 1 (2018): 4441, 10.1038/s41467-018-06953-0. PMC6202321 30361475

[7] M. C. Judson , J. O. Sosa‐Pagan , W. A. Del Cid , J. E. Han , and B. D. Philpot , “Allelic Specificity of Ube3a Expression in the Mouse Brain During Postnatal Development,” Journal of Comparative Neurology 522, no. 8 (2014): 1874–1896, 10.1002/cne.23507. PMC3984624 24254964

[8] J. M. LaSalle , L. T. Reiter , and S. J. Chamberlain , “Epigenetic Regulation of Ube3a and Roles in Human Neurodevelopmental Disorders,” Epigenomics 7, no. 7 (2015): 1213–1228, 10.2217/epi.15.70. PMC4709177 26585570

[9] L. Kalsner and S. J. Chamberlain , “Prader‐Willi, Angelman, and 15q11‐Q13 Duplication Syndromes,” Pediatric Clinics of North America 62, no. 3 (2015): 587–606, 10.1016/j.pcl.2015.03.004. PMC4449422 26022164

[10] K. R. Nash , U. K. Jinwal , and K. M. Bhat , “Ube3a: Bridging the Gap Between Neurodevelopment, Neural Function, and Neurodegenerative Woes,” Journal of Alzheimer's Disease 102, no. 1 (2024): 3–10, 10.1177/13872877241283680. 39497315

[11] C. A. Williams , D. J. Driscoll , and A. I. Dagli , “Clinical and Genetic Aspects of Angelman Syndrome,” Genetics in Medicine 12, no. 7 (2010): 385–395, 10.1097/GIM.0b013e3181def138. 20445456

[12] G. Gillessen‐Kaesbach , S. Demuth , H. Thiele , U. Theile , C. Lich , and B. Horsthemke , “A Previously Unrecognised Phenotype Characterised by Obesity, Muscular Hypotonia, and Ability to Speak in Patients With Angelman Syndrome Caused by an Imprinting Defect,” European Journal of Human Genetics 7, no. 6 (1999): 638–644, 10.1038/sj.ejhg.5200362. 10482951

[13] M.‐L. Brennan , M. P. Adam , L. H. Seaver , et al., “Increased Body Mass in Infancy and Early Toddlerhood in Angelman Syndrome Patients With Uniparental Disomy and Imprinting Center Defects,” American Journal of Medical Genetics Part A 167, no. 1 (2015): 142–146, 10.1002/ajmg.a.36831. 25402239

[14] L. G. Mertz , R. Christensen , I. Vogel , J. M. Hertz , and J. R. Ostergaard , “Eating Behavior, Prenatal and Postnatal Growth in Angelman Syndrome,” Research in Developmental Disabilities 35, no. 11 (2014): 2681–2690, 10.1016/j.ridd.2014.07.025. 25064682

[15] A. M. Larson , J. E. Shinnick , E. A. Shaaya , E. A. Thiele , and R. L. Thibert , “Angelman Syndrome in Adulthood,” American Journal of Medical Genetics Part A 167, no. 2 (2015): 331–344, 10.1002/ajmg.a.36864. PMC5534346 25428759

[16] L. Meng , A. J. Ward , S. Chun , C. F. Bennett , A. L. Beaudet , and F. Rigo , “Towards a Therapy for Angelman Syndrome by Targeting a Long Non‐Coding Rna,” Nature 518, no. 7539 (2015): 409–412, 10.1038/nature13975. PMC4351819 25470045

[17] Y. Liu , S. Lou , J. Li , et al., “Epigenetic Editing Alleviates Angelman Syndrome Phenotype in Mice by Unsilencing Paternal Ube3a,” Cell Discovery 10, no. 1 (2024): 97, 10.1038/s41421-024-00727-3. PMC11405779 39285194

[18] J. Kim , B. Lee , D.‐H. Kim , et al., “UBE3A Suppresses Overnutrition‐Induced Expression of the Steatosis Target Genes of MLL4 by Degrading MLL4,” Hepatology 69, no. 3 (2019): 1122–1134, 10.1002/hep.30284. PMC6393921 30230575

[19] S. Park , A. Aintablian , B. Coupe , and S. G. Bouret , “The Endoplasmic Reticulum Stress‐Autophagy Pathway Controls Hypothalamic Development and Energy Balance Regulation in Leptin‐Deficient Neonates,” Nature Communications 11, no. 1 (2020): 1914, 10.1038/s41467-020-15624-y. PMC7171135 32313051

[20] H. Mao , G. H. Kim , L. Pan , and L. Qi , “Regulation of Leptin Signaling and Diet‐Induced Obesity by Sel1l‐Hrd1 Er‐Associated Degradation in Pomc Expressing Neurons,” Nature Communications 15, no. 1 (2024): 8435, 10.1038/s41467-024-52743-2. PMC11439921 39343970

[21] X. Zhang , G. Zhang , H. Zhang , M. Karin , H. Bai , and D. Cai , “Hypothalamic IKKβ/NF‐κB and ER Stress Link Overnutrition to Energy Imbalance and Obesity,” Cell 135, no. 1 (2008): 61–73, 10.1016/j.cell.2008.07.043. PMC2586330 18854155

[22] L. Ozcan , A. S. Ergin , A. Lu , et al., “Endoplasmic Reticulum Stress Plays a Central Role in Development of Leptin Resistance,” Cell Metabolism 9, no. 1 (2009): 35–51, 10.1016/j.cmet.2008.12.004. 19117545

[23] U. Ozcan , Q. Cao , E. Yilmaz , et al., “Endoplasmic Reticulum Stress Links Obesity, Insulin Action, and Type 2 Diabetes,” Science 306, no. 5695 (2004): 457–461, 10.1126/science.1103160. 15486293

[24] J. F. Tanti , F. Ceppo , J. Jager , and F. Berthou , “Implication of Inflammatory Signaling Pathways in Obesity‐Induced Insulin Resistance,” Frontiers in Endocrinology 3 (2012): 181, 10.3389/fendo.2012.00181. PMC3539134 23316186

[25] I. Cakir , N. E. Cyr , E. Yilmaz , et al., “Obesity Induces Hypothalamic Endoplasmic Reticulum Stress and Impairs Proopiomelanocortin (Pomc) Post‐Translational Processing,” Journal of Biological Chemistry 288, no. 24 (2013): 17675–17688, 10.1074/jbc.M113.475343. PMC3682568 23640886

[26] D. A. Gallegos , U. Chan , L. F. Chen , and A. E. West , “Chromatin Regulation of Neuronal Maturation and Plasticity,” Trends in Neurosciences 41, no. 5 (2018): 311–324, 10.1016/j.tins.2018.02.009. PMC5924605 29530320

[27] M. Jakovcevski and S. Akbarian , “Epigenetic Mechanisms in Neurological Disease,” Nature Medicine 18, no. 8 (2012): 1194–1204, 10.1038/nm.2828. PMC3596876 22869198

[28] J. Park , K. Lee , K. Kim , and S. J. Yi , “The Role of Histone Modifications: From Neurodevelopment to Neurodiseases,” Signal Transduction and Targeted Therapy 7, no. 1 (2022): 217, 10.1038/s41392-022-01078-9. PMC9259618 35794091

[29] W. Gao , J. L. Liu , X. Lu , and Q. Yang , “Epigenetic Regulation of Energy Metabolism in Obesity,” Journal of Molecular Cell Biology 13, no. 7 (2021): 480–499, 10.1093/jmcb/mjab043. PMC8530523 34289049

[30] B. E. Collins , C. B. Greer , B. C. Coleman , and J. D. Sweatt , “Histone H3 Lysine K4 Methylation and Its Role in Learning and Memory,” Epigenetics & Chromatin 12, no. 1 (2019): 7, 10.1186/s13072-018-0251-8. PMC6322263 30616667

[31] I. Maze , K. M. Noh , and C. D. Allis , “Histone Regulation in the Cns: Basic Principles of Epigenetic Plasticity,” Neuropsychopharmacology 38, no. 1 (2013): 3–22, 10.1038/npp.2012.124. PMC3521967 22828751

[32] B. Vanderkruk , N. Maeshima , D. J. Pasula , et al., “Methylation of Histone H3 Lysine 4 Is Required for Maintenance of Beta Cell Function in Adult Mice,” Diabetologia 66, no. 6 (2023): 1097–1115, 10.1007/s00125-023-05896-6. PMC10163146 36912927

[33] P. L. Greer , R. Hanayama , B. L. Bloodgood , et al., “The Angelman Syndrome Protein Ube3a Regulates Synapse Development by Ubiquitinating Arc,” Cell 140, no. 5 (2010): 704–716, 10.1016/j.cell.2010.01.026. PMC2843143 20211139

[34] G. L. Sell and S. S. Margolis , “From Ube3a to Angelman Syndrome: A Substrate Perspective,” Frontiers in Neuroscience 9 (2015): 322, 10.3389/fnins.2015.00322. PMC4569740 26441497

[35] S. Kuhnle , B. Mothes , K. Matentzoglu , and M. Scheffner , “Role of the Ubiquitin Ligase E6ap/Ube3a in Controlling Levels of the Synaptic Protein Arc,” Proceedings of the National Academy of Sciences 110, no. 22 (2013): 8888–8893, 10.1073/pnas.1302792110. PMC3670309 23671107

[36] J. Sun , G. Zhu , Y. Liu , et al., “Ube3a Regulates Synaptic Plasticity and Learning and Memory by Controlling Sk2 Channel Endocytosis,” Cell Reports 12, no. 3 (2015): 449–461, 10.1016/j.celrep.2015.06.023. PMC4520703 26166566

[37] K. A. Jones , J. E. Han , J. P. DeBruyne , and B. D. Philpot , “Persistent Neuronal Ube3a Expression in the Suprachiasmatic Nucleus of Angelman Syndrome Model Mice,” Scientific Reports 6, no. 1 (2016): 28238, 10.1038/srep28238. PMC4910164 27306933

[38] K. Ma , K. Yin , L. Ma , et al., “The Hypothalamic Epigenetic Landscape in Dietary Obesity,” Advanced Science 11, no. 9 (2024): 2306379, 10.1002/advs.202306379. PMC10916675 38115764

[39] N. J. Pandya , S. Meier , S. Tyanova , et al., “A Cross‐Species Spatiotemporal Proteomic Analysis Identifies Ube3a‐Dependent Signaling Pathways and Targets,” Molecular Psychiatry 27, no. 5 (2022): 2590–2601, 10.1038/s41380-022-01484-z. PMC9135630 35264729

[40] I. J. Perron , B. T. Keenan , K. Chellappa , et al., “Dietary Challenges Differentially Affect Activity and Sleep/Wake Behavior in Mus Musculus: Isolating Independent Associations With Diet/Energy Balance and Body Weight,” PLoS ONE 13, no. 5 (2018): 0196743, 10.1371/journal.pone.0196743. PMC5945034 29746501

[41] F. Zambelli , G. Pesole , and G. Pavesi , “Pscanchip: Finding Over‐Represented Transcription Factor‐Binding Site Motifs and Their Correlations in Sequences From Chip‐Seq Experiments,” Nucleic Acids Research 41, no. W1 (2013): W535–W543, 10.1093/nar/gkt448. PMC3692095 23748563

[42] Y. Wei , J. Chen , X. Xu , et al., “Restoration of H3k27me3 Modification Epigenetically Silences Cry1 Expression and Sensitizes Leptin Signaling to Reduce Obesity‐Related Properties,” Advanced Science 8, no. 14 (2021): 2004319, 10.1002/advs.202004319. PMC8292908 34306972

[43] J.‐H. Luo , F.‐X. Wang , J.‐W. Zhao , et al., “Pdia3 Defines a Novel Subset of Adipose Macrophages to Exacerbate the Development of Obesity and Metabolic Disorders,” Cell Metabolism 36, no. 10 (2024): 2262–2280.e5, 10.1016/j.cmet.2024.08.009. 39293433

[44] Y. Xiao , Y. Deng , F. Yuan , et al., “ATF4/ATG5 Signaling in Hypothalamic Proopiomelanocortin Neurons Regulates Fat Mass via Affecting Energy Expenditure,” Diabetes 66, no. 5 (2017): 1146–1158, 10.2337/db16-1546. 28213613

[45] J. Deng , F. Yuan , Y. Guo , et al., “Deletion of Atf4 in Agrp Neurons Promotes Fat Loss Mainly via Increasing Energy Expenditure,” Diabetes 66, no. 3 (2017): 640–650, 10.2337/db16-0954. 27993927

[46] G. Heinrich , K. Meece , S. L. Wardlaw , and D. Accili , “Preserved Energy Balance in Mice Lacking Foxo1 in Neurons of Nkx2.1 Lineage Reveals Functional Heterogeneity of Foxo1 Signaling Within the Hypothalamus,” Diabetes 63, no. 5 (2014): 1572–1582, 10.2337/db13-0651. PMC3994959 24487022

[47] Y. Aponte , D. Atasoy , and S. M. Sternson , “Agrp Neurons Are Sufficient to Orchestrate Feeding Behavior Rapidly and Without Training,” Nature Neuroscience 14, no. 3 (2011): 351–355, 10.1038/nn.2739. PMC3049940 21209617

[48] A. R. Rau and S. T. Hentges , “The Relevance of Agrp Neuron‐Derived Gaba Inputs to Pomc Neurons Differs for Spontaneous and Evoked Release,” The Journal of Neuroscience 37, no. 31 (2017): 7362–7372, 10.1523/JNEUROSCI.0647-17.2017. PMC5546108 28667175

[49] L. E. Cabrera Zapata , M. J. Cambiasso , and M. A. Arevalo , “Epigenetic Modifier Kdm6a/Utx Controls the Specification of Hypothalamic Neuronal Subtypes in a Sex‐Dependent Manner,” Frontiers in Cell and Developmental Biology 10 (2022): 937875, 10.3389/fcell.2022.937875. PMC9577230 36268511

[50] I. Gonzalez‐Garcia , C. Contreras , Á. Estévez‐Salguero , et al., “Estradiol Regulates Energy Balance by Ameliorating Hypothalamic Ceramide‐Induced Er Stress,” Cell Reports 25, no. 2 (2018): 413–423.e5, 10.1016/j.celrep.2018.09.038. PMC6198289 30304681

[51] I. Gonzalez‐Garcia , E. Garcia‐Clave , A. Cebrian‐Serrano , et al., “Estradiol Regulates Leptin Sensitivity to Control Feeding via Hypothalamic Cited1,” Cell Metabolism 35, no. 3 (2023): 438–455.e7, 10.1016/j.cmet.2023.02.004. PMC10028007 36889283

[52] C. F. Bruinsma , M. Schonewille , Z. Gao , et al., “Dissociation of Locomotor and Cerebellar Deficits in a Murine Angelman Syndrome Model,” Journal of Clinical Investigation 125, no. 11 (2015): 4305–4315, 10.1172/JCI83541. PMC4639977 26485287

[53] Q. Xu , M. Tam , and S. A. Anderson , “Fate mapping Nkx2.1‐lineage cells in the mouse telencephalon,” Journal of Comparative Neurology 506, no. 1 (2008): 16–29, 10.1002/cne.21529. 17990269

[54] Q. Tong , C. P. Ye , J. E. Jones , J. K. Elmquist , and B. B. Lowell , “Synaptic Release of Gaba by Agrp Neurons is Required for Normal Regulation of Energy Balance,” Nature Neuroscience 11, no. 9 (2008): 998–1000, 10.1038/nn.2167. PMC2662585 19160495

[55] T. D. Muller , M. Klingenspor , and M. H. Tschop , “Publisher Correction: Revisiting Energy Expenditure: How to Correct Mouse Metabolic Rate for Body Mass,” Nature Metabolism 3, no. 10 (2021): 1433, 10.1038/s42255-021-00485-6. 34625742

[56] C. Huisman , Y. A. Kim , S. Jeon , et al., “The Histone H3‐Lysine 4‐Methyltransferase Mll4 Regulates the Development of Growth Hormone‐Releasing Hormone‐Producing Neurons in the Mouse Hypothalamus,” Nature Communications 12, no. 1 (2021): 256, 10.1038/s41467-020-20511-7. PMC7801453 33431871

[57] R. D. Cardiff , C. H. Miller , and R. J. Munn , “Manual Hematoxylin and Eosin Staining of Mouse Tissue Sections,” Cold Spring Harbor Protocols 2014, no. 6 (2014): 655–658, 10.1101/pdb.prot073411. 24890205

[58] S. Anders and W. Huber , “Differential Expression Analysis for Sequence Count Data,” Genome Biology 11, no. 10 (2010): R106, 10.1186/gb-2010-11-10-r106. PMC3218662 20979621

[59] S. Lee , R. Shen , H.‐H. Cho , et al., “Stat3 Promotes Motor Neuron Differentiation by Collaborating With Motor Neuron‐Specific Lim Complex,” Proceedings of the National Academy of Sciences 110, no. 28 (2013): 11445–11450, 10.1073/pnas.1302676110. PMC3710787 23798382

[60] Y. Liu , F. Cui , A. Xu , et al., “Interaction Between the Perk/Atf4 Branch of the Endoplasmic Reticulum Stress and Mitochondrial One‐Carbon Metabolism Regulates Neuronal Survival After Intracerebral Hemorrhage,” International Journal of Biological Sciences 20, no. 11 (2024): 4277–4296, 10.7150/ijbs.93787. PMC11379068 39247810

