# Supplementary material for: A UBE3A‐UTX Proteostasis‐Epigenetic Axis Governs Hypothalamic Energy Homeostasis
Source: Adv Sci (Weinh). 2026 Oct 29:e78567. Online ahead of print. doi: 10.1002/advs.78567 (PMC13720268; doi:10.1002/advs.78567)
Supplement: Supplementary file 1 [file ADVS-9999-e78567-s001.docx]

Supporting Information

**A UBE3A-UTX Proteostasis-Epigenetic Axis Governs Hypothalamic Energy Homeostasis**

*Junekyoung Lee, Jaeyoung Yoo, Young A.Kim, Zhuodu Wei, Yunseo Lee, Woo-Jeong Shon, Je Kyung Seong and Seunghee Lee**

**Supplemental Figures**

**Figure S1. UBE3A expression in AgRP, POMC, and GHRH neurons of male mice.**

**(a-c)** Colocalization of UBE3A immunoreactivity with GFP in *Npy*-hrGFP neurons **(a)** and with tdTomato in *Pomc*-Cre;*tdTomato*/+ neurons **(b)**, and *Ghrh*-Cre;*tdTomato*/+ neurons **(c)** in the ARC of male mice at postnatal day 40 (P40). Quantification of UBE3A-positive cells among NPY-, POMC-, and GHRH- expressing neuronal populations is shown. The number of sections analyzed is indicated in parenthesis **(a-c)**.

**Figure S2.** **Female *Ube3a*^NKO^ mice develop metabolic dysfunction under normal chow diet conditions.**

**(a)** Body weight gain in female *Ube3a*^con^ and *Ube3a*^NKO^ mice fed an NCD for 12 weeks starting at 8 weeks of age. **(b)** Average daily food intake per mouse during NCD feeding. **(c-e)**, Tissue weights of the liver **(c)**, brown adipose tissue (BAT) **(d)**, and white adipose tissue (WAT) **(e)** in NCD-fed female *Ube3a*^con^ and *Ube3a*^NKO^ mice. **(f,g)** Fat mass-to-lean mass ratio (%) **(f)** and lean mass **(g)** in NCD-fed mice. **(h)** Serum leptin levels in NCD-fed female *Ube3a*^con^ and *Ube3a*^NKO^ mice. **(i-k)** Oxygen consumption (VO_2_) **(i)**, carbon dioxide production (VCO_2_) **(j)**, and respiratory exchange ratio (RER) **(k)** in NCD-fed female *Ube3a*^con^ and *Ube3a*^NKO^ mice. **(l)** Time course of body-weight-normalized energy expenditure (kcal/h/kg) over 48 h, measured by indirect calorimetry in NCD-fed female mice. Gray bars indicate the dark phase. **(m)** Relationship between lean mass and mean absolute energy expenditure over the same 48-h recording period. Absolute energy expenditure is expressed as kcal/h, without normalization to body weight or lean mass. Each point represents an individual mouse. ANCOVA was performed with mean absolute energy expenditure as the dependent variable, lean mass as a continuous covariate, and genotype as a categorical factor. The genotype × lean mass interaction was not statistically significant (*P* = 0.0569). In a common-slope ANCOVA model excluding the interaction term, the genotype effect was significant after adjustment for lean mass (*P* < 0.0001). Lines represent separate linear regression fits for each genotype. **(n)** Core body temperature monitored for 12 h under room-temperature conditions. **(o)** Locomotor activity measured over 48 h by indirect calorimetry in metabolic cages. **(p,q)** Insulin tolerance test (ITT) **(p)** and glucose tolerance test (GTT) **(q)** in NCD-fed female *Ube3a*^con^ and *Ube3a*^NKO^ mice. Blood glucose levels were measured at the indicated time points, and the area under the curve (AUC) was quantified. For (**a-e,h,p,q**), *Ube3a*^con^ (n = 5 mice) and *Ube3a*^NKO^ (n = 7 mice). For (**f,g,i-m,o**), *Ube3a*^con^ (n = 5 mice) and *Ube3a*^NKO^ (n = 6 mice). For (**n**), *Ube3a*^con^ (n = 4 mice) and *Ube3a*^NKO^ (n = 7 mice). Each dot represents one mouse. Data are presented as mean ± s.e.m. Statistical significance was determined using two-way ANOVA followed by Tukey’s multiple-comparisons test **(a,n**), unpaired two-tailed Student’s *t*-test **(b-k)**, and two-way repeated-measures ANOVA with genotype and time as factors, including their interaction, with Geisser–Greenhouse correction (**l,o**). For (**p,q**), blood glucose time-course data were analyzed by two-way ANOVA followed by Tukey's multiple-comparisons test, and AUC values were analyzed using unpaired two-tailed Student's *t*-tests. **P* < 0.05, ***P* < 0.01, *****P* < 0.0001; n.s, not significant.

**Figure S3. Male *Ube3a*^NKO^ mice show largely preserved metabolic homeostasis under normal chow diet conditions.**

**(a)** Body weight gain in male *Ube3a*^con^ and *Ube3a*^NKO^ mice fed an NCD for 12 weeks starting at 8 weeks of age. **(b)** Average daily food intake per mouse during NCD feeding. **(c-e)** Tissue weights of the liver **(c)**, WAT **(d)**, and BAT **(e)** in NCD-fed male *Ube3a*^con^ and *Ube3a*^NKO^ mice. **(f,g)** Fat mass-to-lean mass ratio (%) **(f)** and lean mass **(g)** in NCD-fed mice. **(h)** Relationship between lean mass and mean absolute energy expenditure over the same 48-h recording period. Absolute energy expenditure is expressed as kcal/h, without normalization to body weight or lean mass. Each point represents an individual mouse. ANCOVA was performed with mean absolute energy expenditure as the dependent variable, lean mass as a continuous covariate, and genotype as a categorical factor. The genotype × lean mass interaction was not significant (*P* = 0.7525). In a common-slope ANCOVA model excluding the interaction term, the genotype effect was not significant after adjustment for lean mass (*P* = 0.4274). Lines represent separate linear regression fits for each genotype. **(i,j)** VO_2_ **(i)**, VCO_2_ **(j)** in NCD-fed male *Ube3a*^con^ and *Ube3a*^NKO^ mice. **(k,l)** ITT **(k)** and GTT **(l)** in NCD-fed male *Ube3a*^con^ and *Ube3a*^NKO^ mice. Blood glucose levels were measured at the indicated time points, and the AUC was quantified. For (**a,b**), *Ube3a*^con^ (n = 5 mice) and *Ube3a*^NKO^ (n = 6 mice). For (**c-e**), *Ube3a*^con^ (n = 4 mice) and *Ube3a*^NKO^ (n = 6 mice). For (**f-j**), n = 3 mice per group. For (**k,l**), *Ube3a*^con^ (n = 5 mice) and *Ube3a*^NKO^ (n = 4 mice). Each dot represents one mouse. Data are presented as mean ± s.e.m. Statistical significance was determined using two-way ANOVA followed by Tukey’s multiple-comparisons test **(a)** and unpaired two-tailed Student’s *t*-test **(b-g,i,j)**. For (**k,l**), blood glucose time-course data were analyzed by two-way ANOVA followed by Tukey's multiple-comparisons test, and AUC values were analyzed using unpaired two-tailed Student's *t*-tests. n.s, not significant.

**Figure S4. Male *Ube3a*^NKO^ mice show largely preserved metabolic homeostasis under high-fat diet conditions.** (**a)** Body weight gain in male *Ube3a*^con^ and *Ube3a*^NKO^ mice fed an HFD for 12 weeks starting at 8 weeks of age. **(b)** Average daily food intake per mouse during HFD feeding. **(c-e)** Tissue weights of the liver **(c)**, WAT **(d)**, and BAT **(e)** in HFD-fed male *Ube3a*^con^ and *Ube3a*^NKO^ mice. **(f)** Core body temperature monitored for 12 h under room temperature and cold-exposure conditions. **(g,h)** ITT **(g)** and GTT **(h)** in HFD-fed male *Ube3a*^con^ and *Ube3a*^NKO^ mice. Blood glucose levels were measured at the indicated time points, and the AUC was quantified. For (**a,b,g,h**), *Ube3a*^con^ (n = 5 mice) and *Ube3a*^NKO^ (n = 6 mice). For (**c-f**), *Ube3a*^con^ (n = 4 mice) and *Ube3a*^NKO^ (n = 6 mice). Each dot represents one mouse. Data are presented as mean ± s.e.m. Statistical significance was determined using two-way ANOVA followed by Tukey’s multiple-comparisons test **(a,f)** or unpaired two-tailed Student’s *t*-test **(b-e)**. For (**g,h**), blood glucose time-course data were analyzed by two-way ANOVA followed by Tukey's multiple-comparisons test, and AUC values were analyzed using unpaired two-tailed Student's *t*-tests. n.s, not significant.

**Figure S5. AgRP neuron-specific deletion of *Ube3a* in female mice does not significantly alter metabolic parameters under normal chow diet conditions.** **(a)** Body weight gain in female *Ube3a*^con^ and *Ube3a*^AKO^ mice fed an NCD for 12 weeks starting at 8 weeks of age. **(b)** Average daily food intake per mouse during NCD feeding. **(c-e)** Tissue weights of the liver **(c)**, WAT **(d)**, and BAT **(e)** in NCD-fed female *Ube3a*^con^ and *Ube3a*^AKO^ mice. **(f,g)** Fat mass-to-lean mass ratio (%) **(f)** and lean mass **(g)** in NCD-fed mice (*n* = 3 mice per group). **(h)** Relationship between lean mass and mean absolute energy expenditure over the same 48-h recording period. Absolute energy expenditure is expressed as kcal/h, without normalization to body weight or lean mass. Each point represents an individual mouse. ANCOVA was performed with mean absolute energy expenditure as the dependent variable, lean mass as a continuous covariate, and genotype as a categorical factor. The genotype × lean mass interaction was not significant (*P* = 0.3354). In a common-slope ANCOVA model excluding the interaction term, the genotype effect was not significant after adjustment for lean mass (*P* = 0.2062). Lines represent separate linear regression fits for each genotype. **(i,j)** VO_2_ **(i)**, VCO_2_ **(j)** in NCD-fed female *Ube3a*^con^ and *Ube3a*^AKO^ mice. **(k,l)** ITT **(k)** and GTT **(l)** in NCD-fed in NCD-fed female *Ube3a*^con^ and *Ube3a*^AKO^ mice. Blood glucose levels were measured at the indicated time points, and the AUC was quantified. For (**a-e**), n = 5 mice per group. For (**f-j**), n = 3 mice per group. For (**k,l**), *Ube3a*^con^ (n = 5 mice) and *Ube3a*^AKO^ (n = 4-5 mice). Each dot represents one mouse. Data are presented as mean ± s.e.m. Statistical significance was determined using two-way ANOVA followed by Tukey’s multiple-comparisons test **(a)** and unpaired two-tailed Student’s *t*-test **(b-g,i,j)**. For (**k,l**), blood glucose time-course data were analyzed by two-way ANOVA followed by Tukey's multiple-comparisons test, and AUC values were analyzed using unpaired two-tailed Student's *t*-tests. **P* < 0.05; n.s, not significant.

**Figure S6. Male *Ube3a*^AKO^ mice maintain metabolic homeostasis under normal chow diet conditions.** **(a)** Body weight gain in male *Ube3a*^con^ and *Ube3a*^AKO^ mice fed an NCD for 11 weeks starting at 8 weeks of age. **(b)** Average daily food intake per mouse during NCD feeding. **(c-e)** Tissue weights of the liver **(c)**, WAT **(d)**, and BAT **(e)** in NCD-fed male *Ube3a*^con^ and *Ube3a*^AKO^ mice. **(f,g)** Fat mass-to-lean mass ratio (%) **(f)** and lean mass **(g)** in NCD-fed mice. **(h)** Relationship between lean mass and mean absolute energy expenditure over the same 48-h recording period. Absolute energy expenditure is expressed as kcal/h, without normalization to body weight or lean mass. Each point represents an individual mouse. ANCOVA was performed with mean absolute energy expenditure as the dependent variable, lean mass as a continuous covariate, and genotype as a categorical factor. The genotype × lean mass interaction was not significant (*P* = 0.5963). In a common-slope ANCOVA model excluding the interaction term, the genotype effect was not significant after adjustment for lean mass (*P* = 0.4359). Lines represent separate linear regression fits for each genotype. **(i,j)** VO_2_ **(i)**, VCO_2_ **(j)** in NCD-fed male *Ube3a*^con^ and *Ube3a*^AKO^ mice. **(k,l)** ITT **(k)** and GTT **(l)** in NCD-fed male *Ube3a*^con^ and *Ube3a*^AKO^ mice. Blood glucose levels were measured at the indicated time points, and the AUC was quantified. For (**a-l**), n = 3 mice per group. Each dot represents one mouse. Data are presented as mean ± s.e.m. Statistical significance was determined using two-way ANOVA followed by Tukey’s multiple-comparisons test **(a)** and unpaired two-tailed Student’s *t*-test **(b-g,i,j)**. For (**k,l**), blood glucose time-course data were analyzed by two-way ANOVA followed by Tukey's multiple-comparisons test, and AUC values were analyzed using unpaired two-tailed Student's *t*-tests. n.s, not significant.

**Figure S7. Male *Ube3a*^AKO^ mice exhibit limited metabolic responses to high-fat diet feeding.** **(a)** Body weight gain in male *Ube3a*^con^ and *Ube3a*^AKO^ mice fed an HFD for 11 weeks starting at 8 weeks of age. **(b)** Average daily food intake per mouse during HFD feeding. **(c-e)** Tissue weights of the liver **(c)**, WAT **(d)**, and BAT **(e)** in HFD-fed male *Ube3a*^con^ and *Ube3a*^AKO^ mice. **(f,g)** Fat mass-to-lean mass ratio (%) **(f)** and lean mass **(g)** in HFD-fed mice. **(h)** Relationship between lean mass and mean absolute energy expenditure over the same 48-h recording period. Absolute energy expenditure is expressed as kcal/h, without normalization to body weight or lean mass. Each point represents an individual mouse. ANCOVA was performed with mean absolute energy expenditure as the dependent variable, lean mass as a continuous covariate, and genotype as a categorical factor. The genotype × lean mass interaction was not significant (*P* = 0.5902). In a common-slope ANCOVA model excluding the interaction term, the genotype effect was not significant after adjustment for lean mass (*P* = 0.8785). Lines represent separate linear regression fits for each genotype. **(i,j)** VO_2_ **(i)**, VCO_2_ **(j)** in HFD-fed male *Ube3a*^con^ and *Ube3a*^AKO^ mice. **(k,l)** ITT **(k)** and GTT **(l)** in HFD-fed male *Ube3a*^con^ and *Ube3a*^AKO^ mice. Blood glucose levels were measured at the indicated time points, and the AUC was quantified. For (**a-l**), n = 3 mice per group. Each dot represents one mouse. Data are presented as mean ± s.e.m. Statistical significance was determined using two-way ANOVA followed by Tukey’s multiple-comparisons test **(a)** and unpaired two-tailed Student’s *t*-test **(b-g,i,j)**. For (**k,l**), blood glucose time-course data were analyzed by two-way ANOVA followed by Tukey's multiple-comparisons test, and AUC values were analyzed using unpaired two-tailed Student's *t*-tests. **P* < 0.05; n.s, not significant.

**Figure S8. Chemogenetic inhibition of AgRP neurons restores POMC-melanocortin signaling in female *Ube3a*^AKO^ mice.**

**(a)** Schematic representation of the chemogenetic inhibition of AgRP neurons. Twelve-week-old female *Ube3a*^con^ and *Ube3a*^AKO^ mice were stereotaxically injected with AAV-hSyn-DIO-mCherry or AAV-hSyn-DIO-hM4Di-mCherry into the ARC. Representative image showing mCherry expression in the ARC three weeks after viral injection. **(b)** Representative in situ hybridization (ISH) images and quantification of *Pomc* mRNA expression in the ARC of *Ube3a*^con^ mice injected with mCherry control virus, *Ube3a*^AKO^ mice injected with mCherry control virus, and *Ube3a*^AKO^ mice injected with hM4Di-mCherry followed by CNO administration. **(c)** Representative immunofluorescence images and quantification of POMC expression in the ARC. **(d)** Representative immunofluorescence images and quantification of AgRP immunoreactivity in the PVN. **(e)** Representative immunofluorescence images and quantification of α-MSH immunoreactivity in the PVN. CNO (3 mg/kg) was administrated 3 weeks after viral injection, and mice were perfused 3 h after CNO administration. Scale bars, 100 μm. Data are presented as mean ± s.e.m. Statistical significance was determined using one-way ANOVA followed by Tukey’s multiple-comparisons test. **P* < 0.05, ****P* < 0.001, *****P* < 0.0001.

**Figure S9. Sex-specific regulation of the UBE3A–UTX–H3K27me3 axis in the hypothalamus.** **(a)** Relative protein abundance of MLL4 and EZH2 in cortical tissues from control and Angelman syndrome (AS) model mice at P56, normalized to control levels (*n* = 3-6 biologically independent samples per group). Data were obtained from a previously published proteomics dataset. **(b)** Immunoblot analysis of hypothalamic lysates from 2-month-old male *Ube3a*^con^ and *Ube3a*^NKO^ mice fed NCD, using antibodies against UBE3A, UTX, H3K27me3, H3, and GAPDH (*n* = 3 mice per group). Band intensities of UBE3A and UTX were quantified using ImageJ and normalized to GAPDH, and H3K27me3 intensity was normalized to H3. Each dot represents one mouse. Data are presented as mean ± s.e.m. Statistical significance was determined using unpaired two-tailed Student’s *t*-test. ***P* < 0.01; n.s, not significant.

**Figure S10. UBE3A catalytic activity promotes UTX ubiquitination and protein degradation. (a)** Immunoblot analysis of mHypoE-N41 cells co-expressing HA–UBE3A WT with 6×Myc-tagged UTX WT, UTX K867R, or UTY. Immunoblots were probed with antibodies against Myc, HA, and GAPDH. UBE3A WT induced dose-dependent degradation of UTX WT, whereas UTX K867R mutant or UTY protein levels were not reduced by UBE3A expression. For each construct, protein levels in the absence of HA-UBE3A (-) were normalized to 100%. **(b)** Cycloheximide (CHX)-chase analysis of UTX protein stability in mHypoE-N41 WT and *Ube3a*-KO cells expressing 6xMyc-tagged UTX WT together with empty vector, HA-UBE3A WT, or HA-UBE3A C820A mutant. Cells were treated with CHX (200 ng/ml), and UTX protein levels were analyzed at the indicated time points. UTX protein levels at the beginning of the chase (0 h) were normalized to 100% for each condition.

**Figure S11. UBE3A deficiency induces ER stress-associated transcriptional and proteomic changes.** **(a)** Representative ISH images and quantification showing increased expression of ER stress-related genes, including *Pdia3*, *Pdia6*, *Dnajc3*, *Dnm1l*, and *Ubqln1*, in the ARC of HFD-fed female *Ube3a*^NKO^ mice compared with *Ube3a*^con^ mice (*n* = 3 mice per group). Scale bars, 100 μm. Each dot represents one mouse. **(b)** Relative protein abundance of PDIA3, PDIA6, DNAJC3, DNM1L, and UBQLN1 in cortical tissues from control and Angelman syndrome (AS) model mice at P56, normalized to control levels (*n* = 4-6 biologically independent samples per group). Data were obtained from a previously published proteomics dataset. Each dot represents one mouse. Data are presented as mean ± s.e.m. Statistical significance was determined using unpaired two-tailed Student’s *t*-test. **P* < 0.05, ***P* < 0.01.

**Figure S12. UTX and ATF4 CUT&Tag profiles at selected ER stress-associated loci. (a)** Representative UTX CUT&Tag tracks from mouse hypothalamic tissue and ATF4 CUT&Tag tracks from mouse primary neurons at the *Atf4*, *Pdia3*, *Pdia6*, *Dnajc3*, *Dnm1l*, and *Ubqln1* loci. Red boxes highlight selected genomic regions containing peaks in the respective datasets. These datasets were generated from distinct neural preparations and do not establish simultaneous UTX–ATF4 occupancy in the same cells. **(b)** Representative immunohistochemistry (IHC) images and quantification showing increased ATF4 expression in OTP-positive AgRP neurons in NCD-fed female *Ube3a*^NKO^ mice at P40 compared with *Ube3a*^con^ mice (*n* = 7 and 6 mice per group, respectively). Scale bars, 100 μm. Representative images from biologically independent samples are shown. Each dot represents one mouse. Data are presented as mean ± s.e.m. Statistical significance was determined using unpaired two-tailed Student’s *t*-test. *****P* < 0.0001.

| **Supplementary Tables.**  **Table S1. Oligonucleotides used in the study** | | | | |
| --- | --- | --- | --- | --- |
| **Gene symbol** | **Forward** | | **Reverse** | |
| **Mouse genotyping primers** | | | | |
| *Ube3a* flox | AAAATTGGGTATGCGAGCTG | | GGGGTCTAAGGGCCTATGAA | |
| *Nkx2.1-Cre* | GTGTTGCCGCGCCATCTGC | | CACCATTGCCCCTGTTTCACTATC | |
| *AgRP-IRES-Cre* | GCTTCTTCAATGCCTTTTGC (Common) | | AGGAACTGCTTCCTTCACGA (Mutant) | |
|  |  |  | GTGTGTGGTTCCAGCATGAC (WT) | |
|  |  | |  | |
| **Mouse qPCR primers** | | | | |
| *mUcp1* | CACCTTCCCGCTGGACACT | | CCCTAGGACACCTTTATACCTAATGG | |
| *mUcp2* | ACCATTGCACGAGAGGAAGG | | TCTTGACCACATCAACGGGG | |
| *mβ3-AR* | GCTCCGTGGCCTCACGAGAA | | CCCAACGGCCAGTGGCCAGTCAGCG | |
| *mDio2* | AATTATGCCTCGGAGAAGACCG | | GGCAGTTGCCTAGTGAAAGGT | |
| *mPrdm16* | TGAGCCCCAAGGAGTCTATG | | GCGTGGAGAGGAGTGTCTTC | |
| *mCyclophilin A* | GTCTCCTTCGAGCTGTTTGC | | GATGCCAGGACCTGTATGCT | |
|  | | | | |
| ***Pdia3*-ATF4 reporter construct primers** | | | | |
| *mPdia3*-ATF4 | ACGCGTCGACGCACACTATCAGTCTCAGCC | | CGGGATCCTGGTTTGTTTGAGAGGGTGG | |
|  |  | |  | |
| **shRNA primers** | | | | |
| *mUbe3a* | GATCCCCCAATGATGTATGATCTAAATTCAAGAGATTTAGATCATACA TCATTGGGTTTTTA | | AGCTTAAAAACCCAATGATGTATG ATCTAAATCTCTTGAATTTAGATC ATACATCATTGGGG | |
|  |  | |  | |
| **Guide RNAs** |  | |  | |
| *mUbe3a* | CACAGCCCTCAGTTAACTGA | | TCAGTTAACTGAGGGCTGTG | |
|  | | | | |
| **ChIP primers** | | | | |
| *mPdia3*-ATF4 | GCACACTATCAGTCTCAGCC | | GGATTGAGCCCAGAACCTTG | |
| *mPdia6*-ATF4 | GGGAGGAAAGGGTTTGGTTT | | TAGTGATTGAAAGGGCCCAG | |
| *mDnajc3*-ATF4 | CTACCGCTGTTTTCCGCC | | AGCAGGAACGGGAACACC | |
| *mDnm1l*-ATF4 | *ACACTCAACTGCAGGAAACAC* | | *GCATTTTCCCATTCCTGCCA* | |
| *mUbqln1*-ATF4 | GTGTCTGTCTCGTCTCCCTA | | TGCTGAACTCCTTCCCATCT | |
| *mUntr6* | TCAGGCATGAACCACCATAC | | AACATCCACACGTCCAGTGA | |
|  |  | |  | |
|  | |  | |  |
|  | |  | |  |
|  | |  | |  |
|  | |  | |  |
|  | |  | |  |
|  | |  | |  |

| **Table S2. Antibodies used in the study.** | | | |
| --- | --- | --- | --- |
| Antibodies | Source | Application | Cat#, Lot#, RRID |
| UBE3A | BETHYL | IHC | A300-352A, 3, AB_2212948 (RRID) |
| POMC | Phoenix Pharmaceutical | IHC | H-029-30, AB_2307442 (RRID) |
| AgRP | Phoenix Pharmaceutical | IHC | H-003-57, AB_2313909 (RRID) |
| α-MSH | ImmunoStar | IHC | 20074, AB_572218 (RRID) |
| OTP | Homemade (DOI: https://doi.org/https://doi.org/10.1038/ s41467-018-04377-4) | IHC |  |
| H3K27me3 | Millipore | IHC | 07-449, 3845703, AB_310624 (RRID) |
| UTX | Millipore | IHC | ABE1865, 3537564, AB_2893187 (RRID) |
| H3K4me1 | Abcam | IHC | ab8895, GR3426435-2, AB_306847 (RRID) |
| H3K4me2 | Millipore | IHC | 07-030, AB_11213050 (RRID) |
| H3K4me3 | Abcam | IHC | Ab8580, AB_306649 (RRID) |
| ATF4 | Invitrogen | IHC | PA5-27576, AB4631088, AB_2545052 (RRID) |
| UBE3A | BETHYL | WB | A300-352A, 3, AB_2212948 (RRID) |
| UTX | Millipore | WB | ABE1865, 3537564, AB_2893187 (RRID) |
| GAPDH | Proteintech | WB | 60004-4-lg, AB_2107436 (RRID) |
| H3K27me3 | Millipore | WB | 07-449, 3845703, AB_310624 (RRID) |
| H3K4me1 | Abcam | WB | ab8895, GR3426435-2, AB_306847 (RRID) |
| H3K4me2 | Millipore | WB | 07-030, AB_11213050 (RRID) |
| H3K4me3 | Abcam | WB | Ab8580, AB_306649 (RRID) |
| H3 | Abcam | WB | ab176842, GR3218247-4, AB_2715502 (RRID) |
| JMJD3 | Abcam | WB | ab38113, AB_943898 (RRID) |
| RBbP5 | Santa Cruz | WB | sc-271071 |
| PTIP | Santa Cruz | WB | sc-293322 |
| myc | Millipore | WB | 05-419, AB_309725 (RRID) |
| HA | Covance | WB | MMS-101R, AB_291262 (RRID) |
| β-tubulin | Santa Cruz | WB | sc-9104, H2015, AB_2241191 (RRID) |
| ATF4 | Invitrogen | WB | PA5-27576, AB4631088, AB_2545052 (RRID) |
| IgG (rabbit) | Invitrogen | IP, ChIP | 02-6102, VJ310383, AB_2532938 (RRID) |
| Myc | Millipore | IP | 05-419, AB_309725 (RRID) |
| UTX | Millipore | IP, ChIP, CUT&Tag | ABE1865, 3537564, AB_2893187 (RRID) |
| UBE3A | BETHYL | IP | A300-352A, 3, AB_2212948 (RRID) |
| ATF4 | Invitrogen | ChIP | PA5-27576, AB4631088, AB_2545052 (RRID) |
| H3K27me3 | Millipore | ChIP | 07-449, 3845703, AB_310624 (RRID) |
| DIG-AP | Roche | ISH | 65752121, 11093274910 |
| Alexa fluor 488 rabbit | Invitrogen | IHC (secondary antibody) | A11034, 2861864 |
| Alexa fluor 594 guinea pig | Invitrogen | IHC (secondary antibody) | A11076, 2920653 |
| Goat anti-Rabbit IgG (H+L), HRP | Invitrogen | WB (secondary antibody) | 31460 |
| Goat anti-Mouse IgG, HRP | Thermo Fisher Scientific | WB (secondary antibody) | 31430 |
