# Supplementary material for: A UBE3A‐UTX Proteostasis‐Epigenetic Axis Governs Hypothalamic Energy Homeostasis
Source: Adv Sci (Weinh). 2026 Oct 29:e78567. Online ahead of print. doi: 10.1002/advs.78567 (PMC13720268; doi:10.1002/advs.78567)
Supplement: Supplementary file 2 [file ADVS-9999-e78567-s002.zip › UBE3A Source Data/UBE3A Source data_microscope images.pdf]

Source Data Figure 1a.

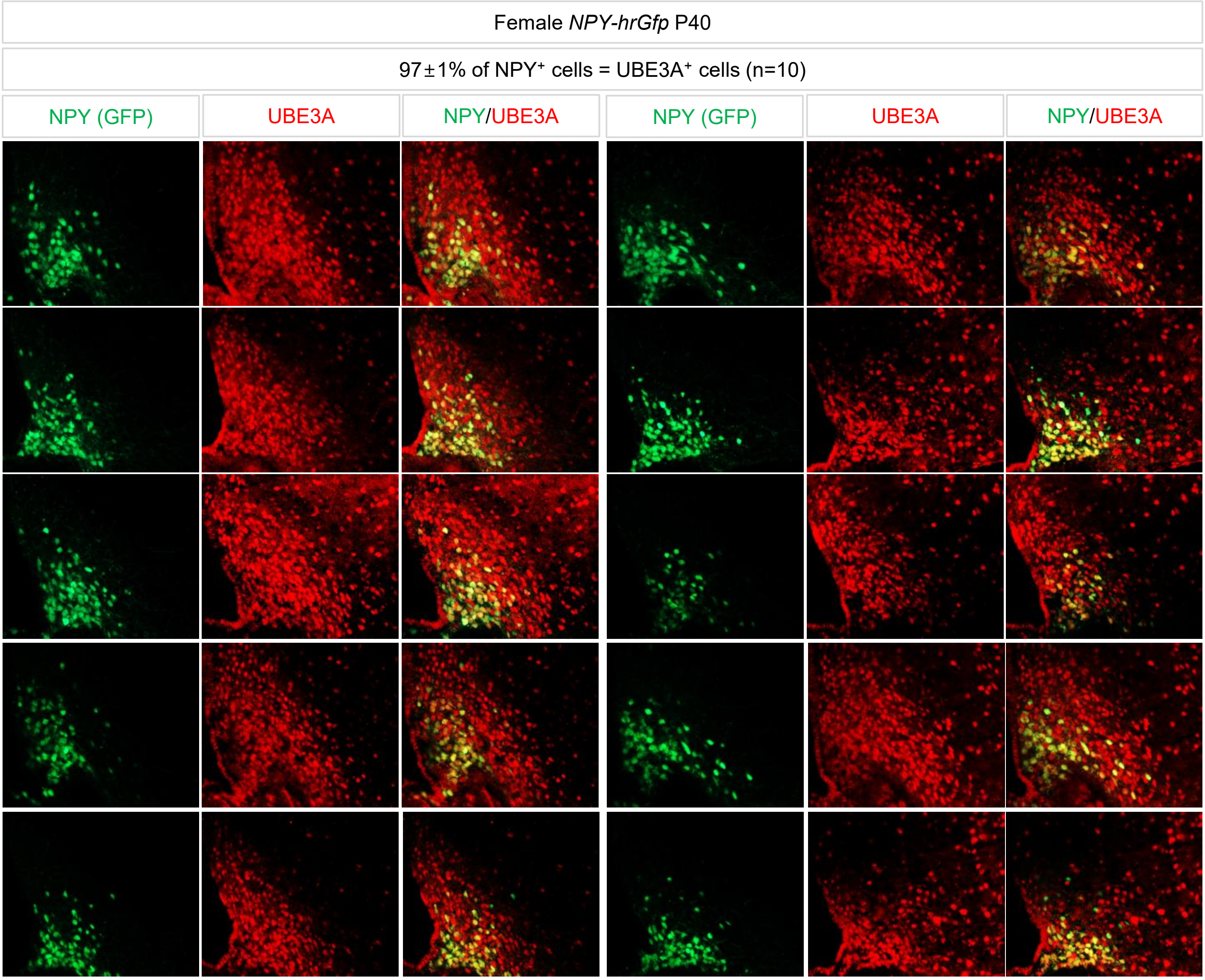

Source Data Figure 1b.

Female *Pomc-Cre;tdTomato/+* (P40)

78±2% of POMC$^+$ cells = UBE3A$^+$ cells (n=8)

UBE3A	POMC	UBE3A POMC	UBE3A	POMC	UBE3A POMC

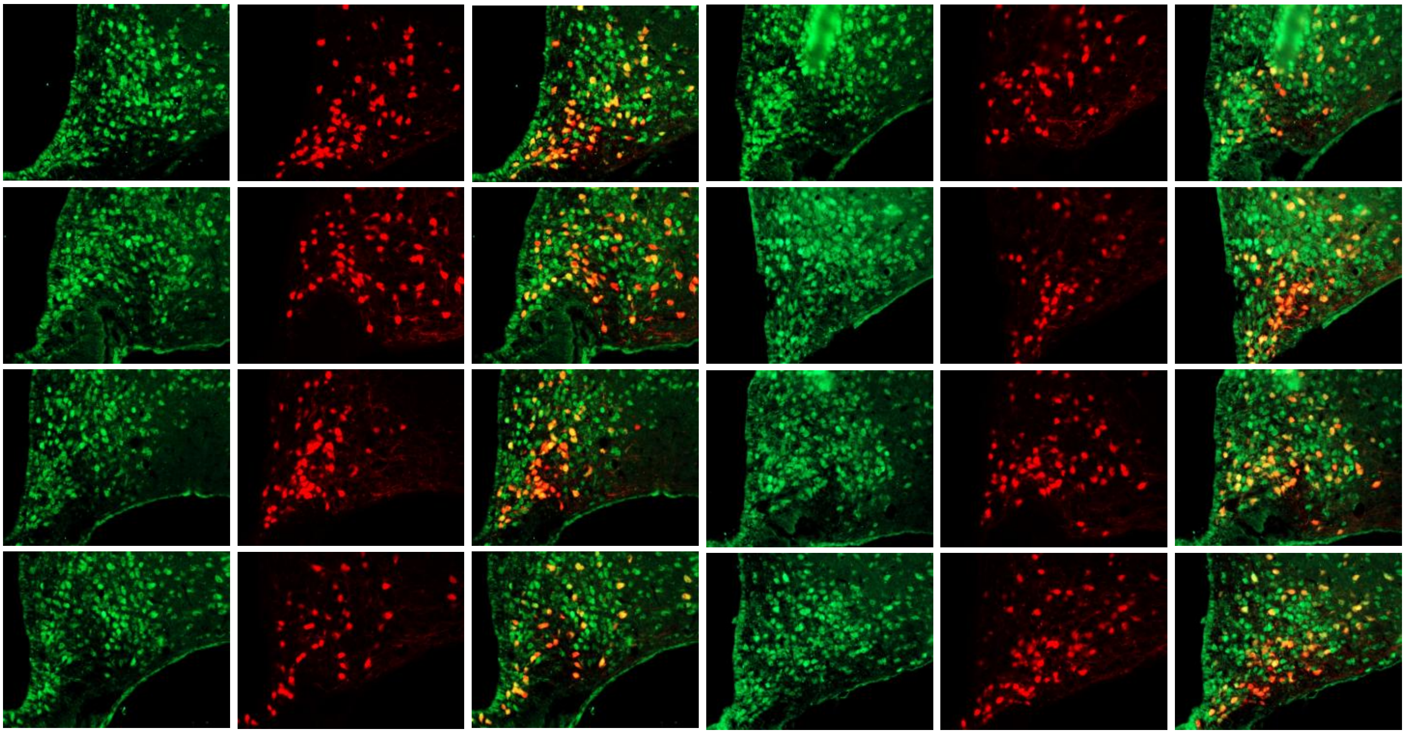

Source Data Figure 1c.

Female *Ghrh-Cre;tdTomato/+* (P40)					
75±3% of GHRH$^+$ cells = UBE3A$^+$ cells (n=10)					
UBE3A	GHRH	UBE3A GHRH	UBE3A	GHRH	UBE3A GHRH

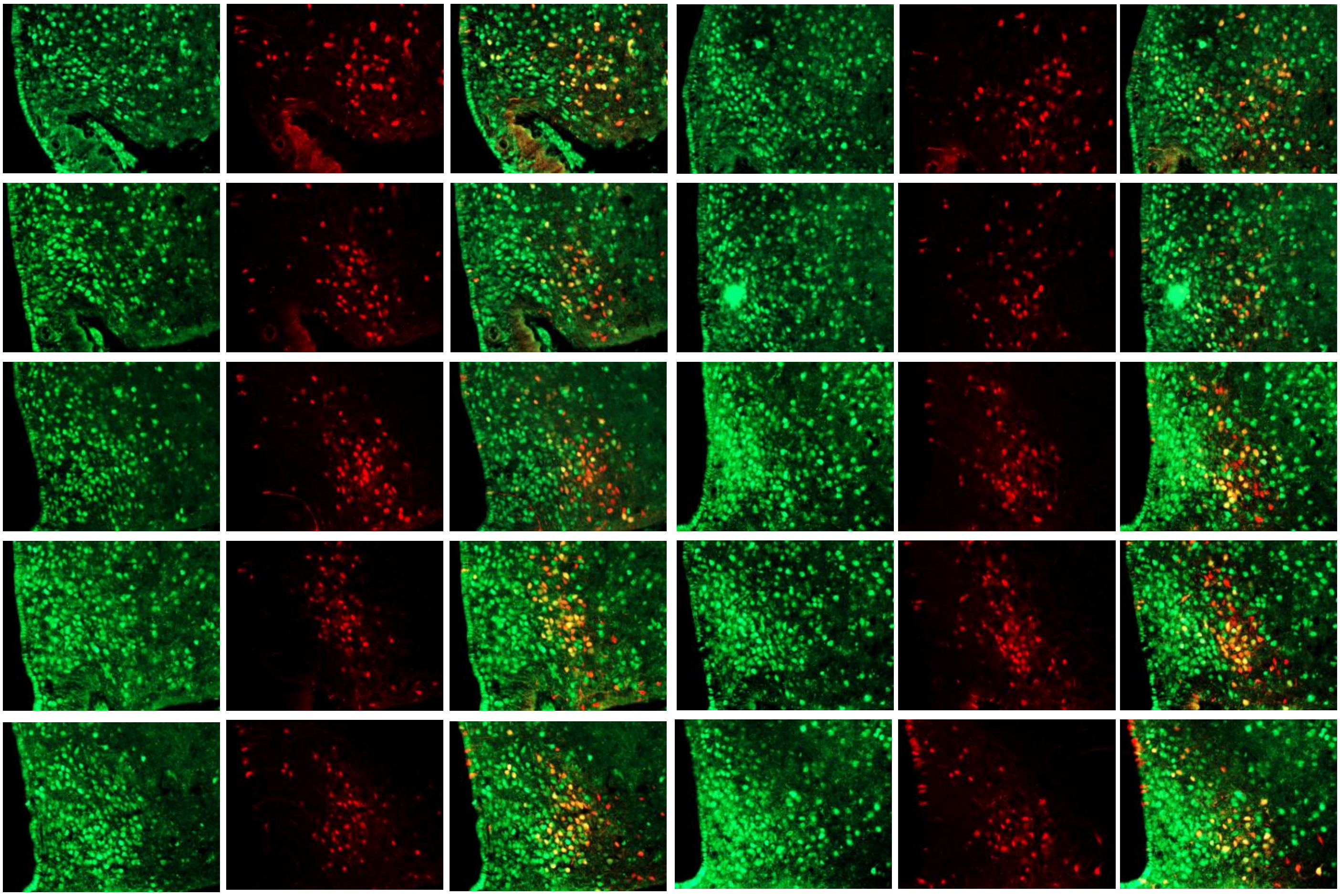

Source Data Figure 1e.

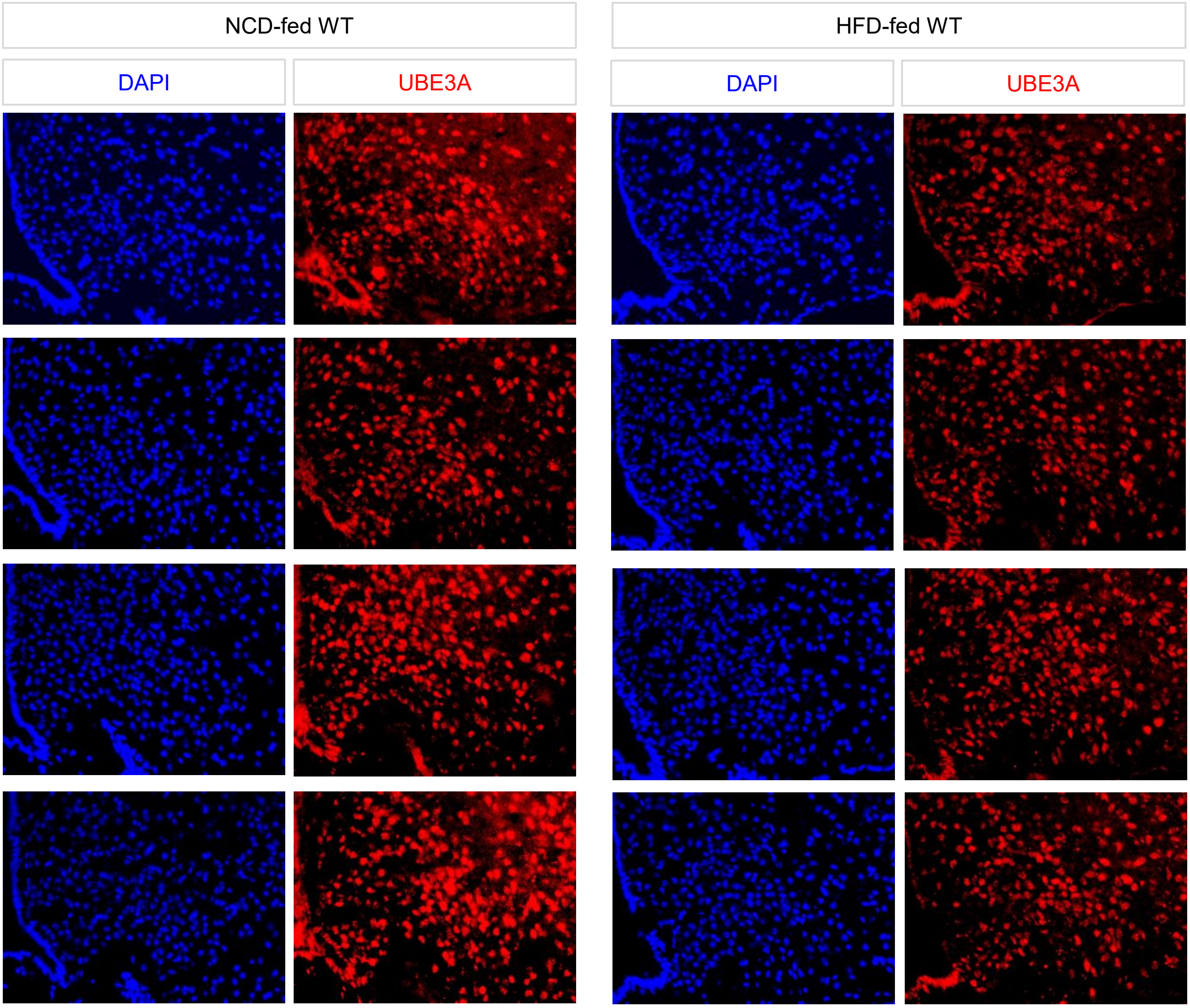

A representative image from each mouse is included.

Source Data Figure 1j.

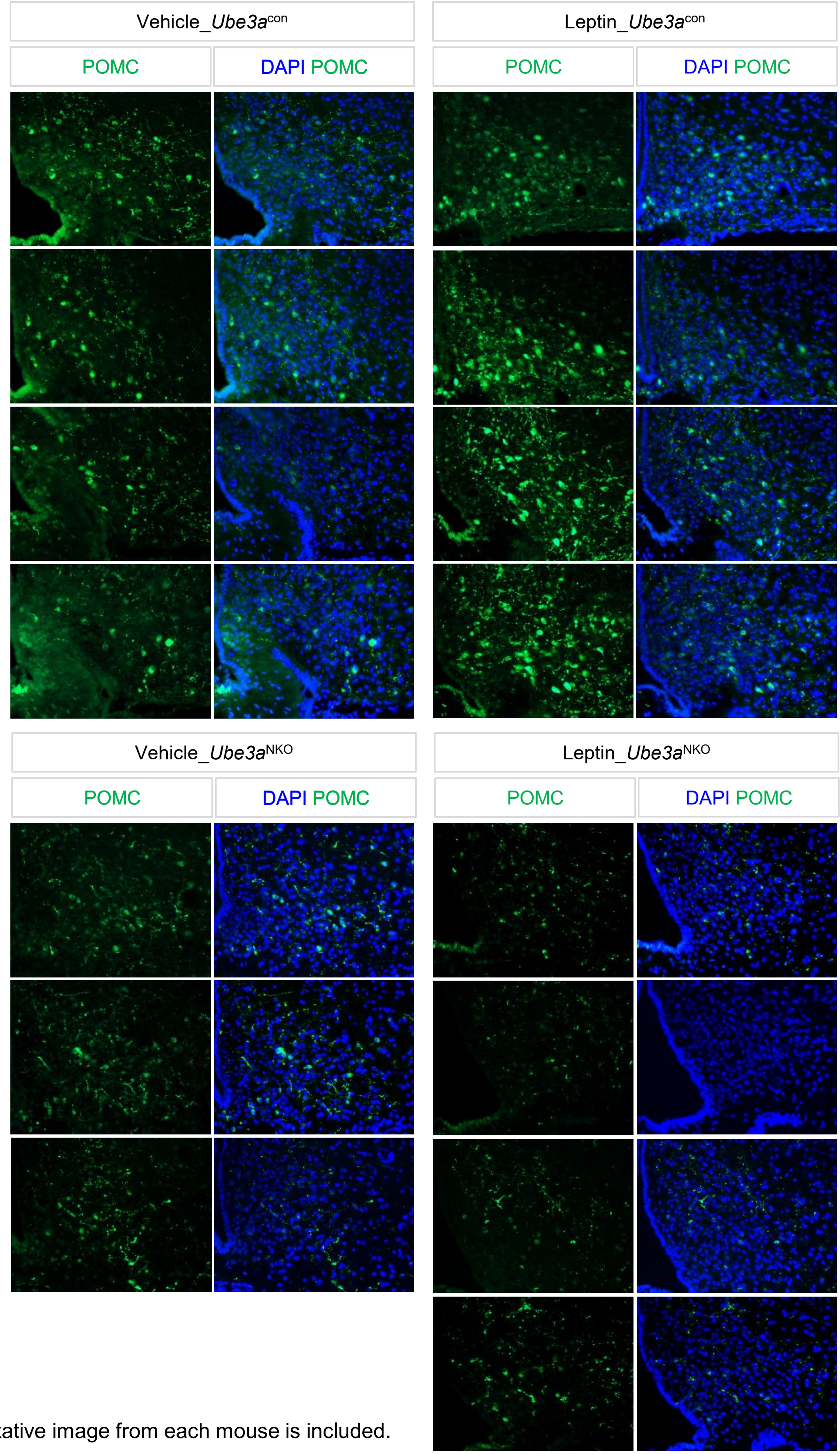

A representative image from each mouse is included.

Source Data Figure 2i.

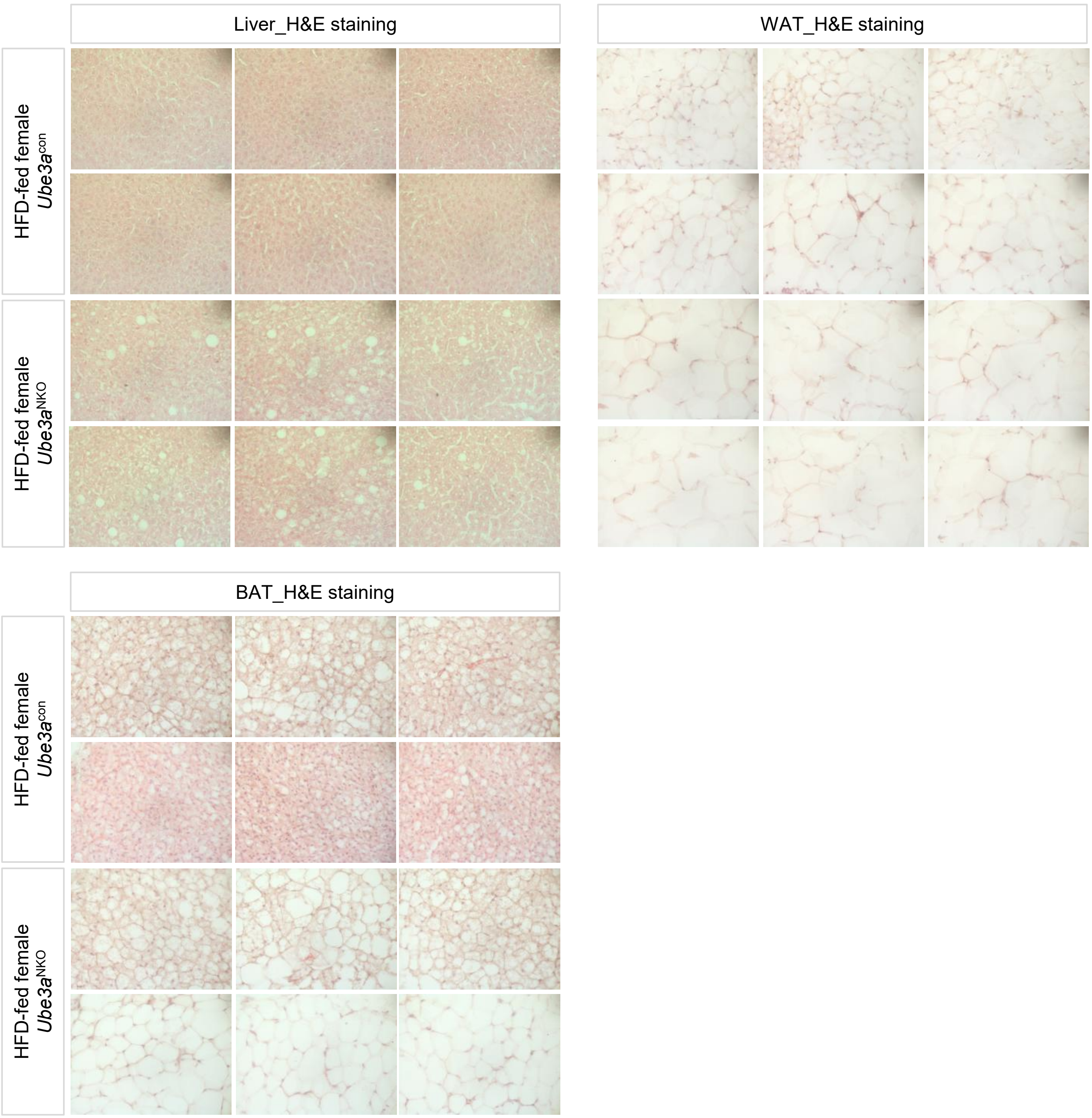

A representative image from each mouse is included.

Source Data Figure 2q.

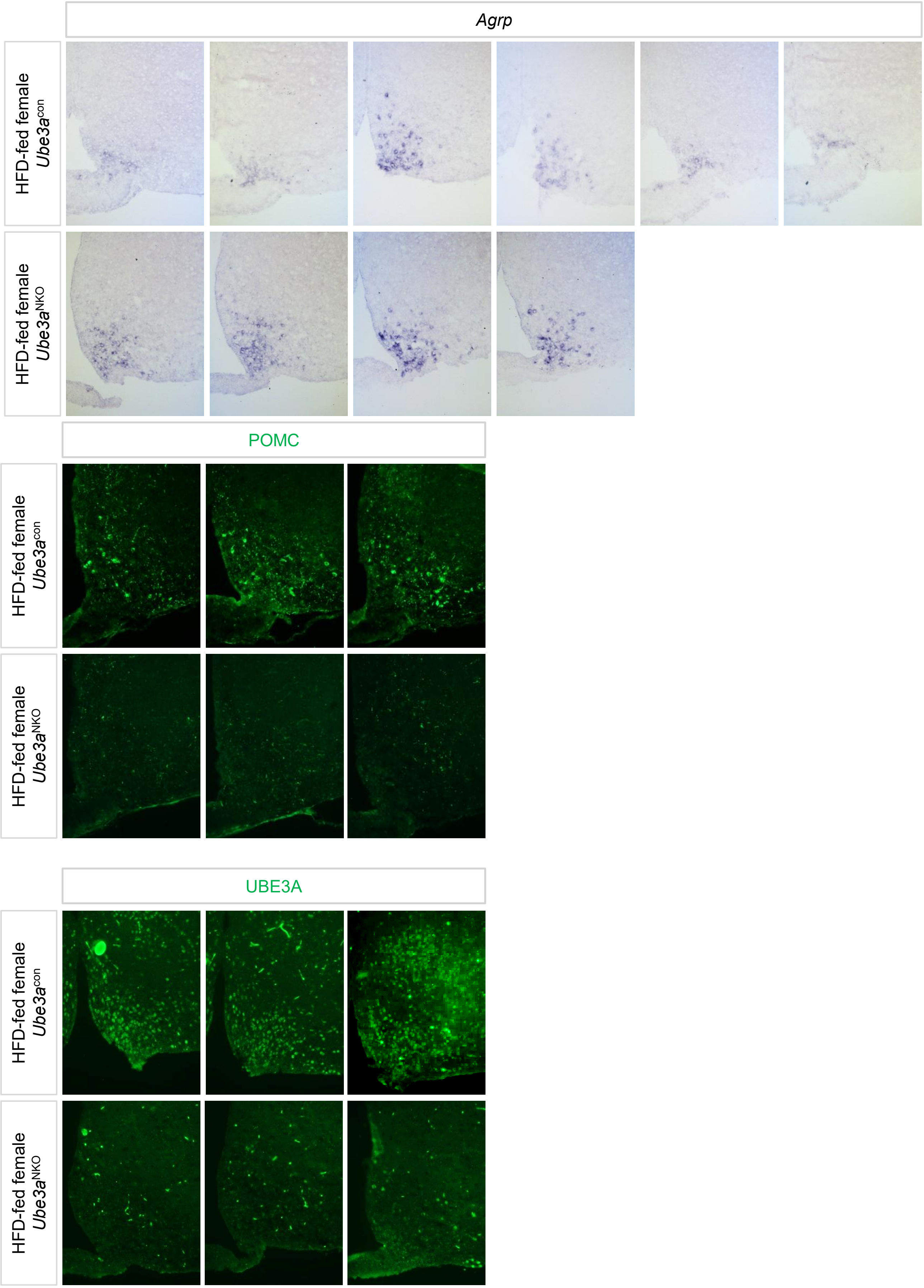

A representative image from each mouse is included.

Source Data Figure 3h.

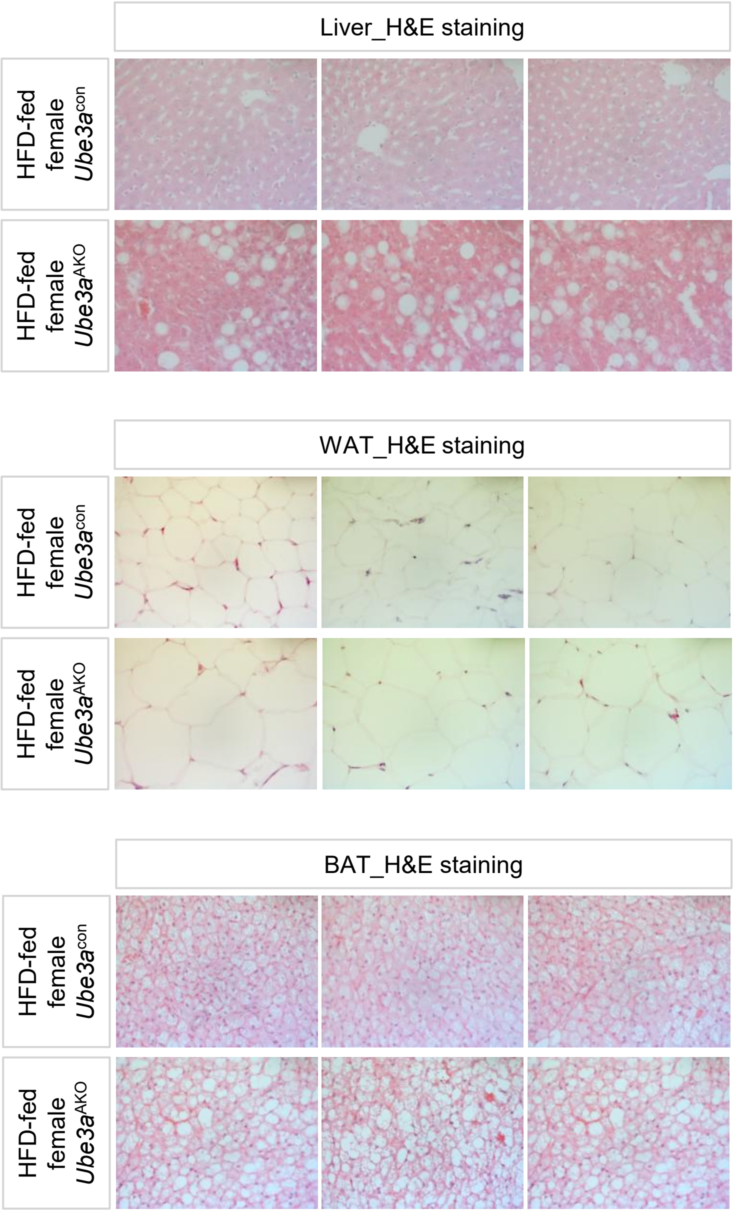

A representative image from each mouse is included.

Source Data Figure 3s.

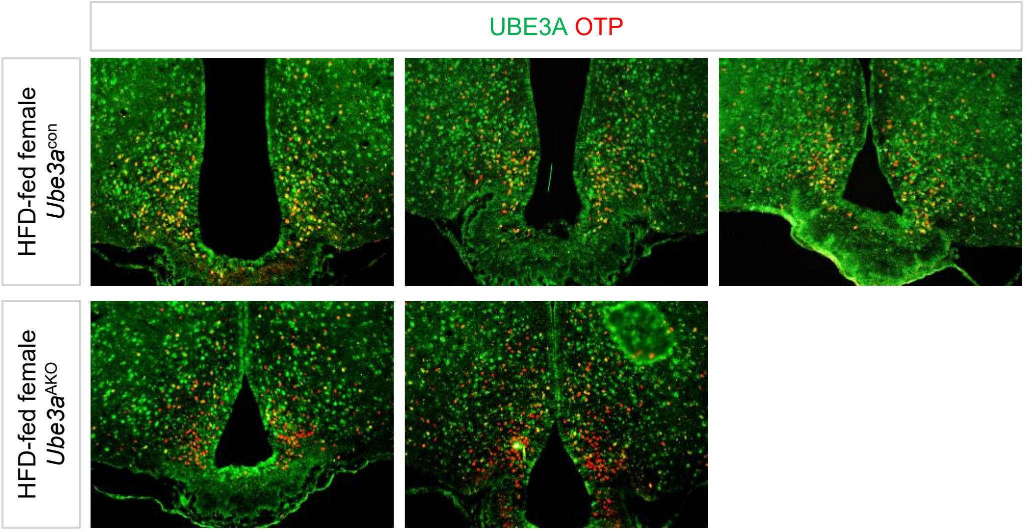

A representative image from each mouse is included.

Source Data Figure 3t.

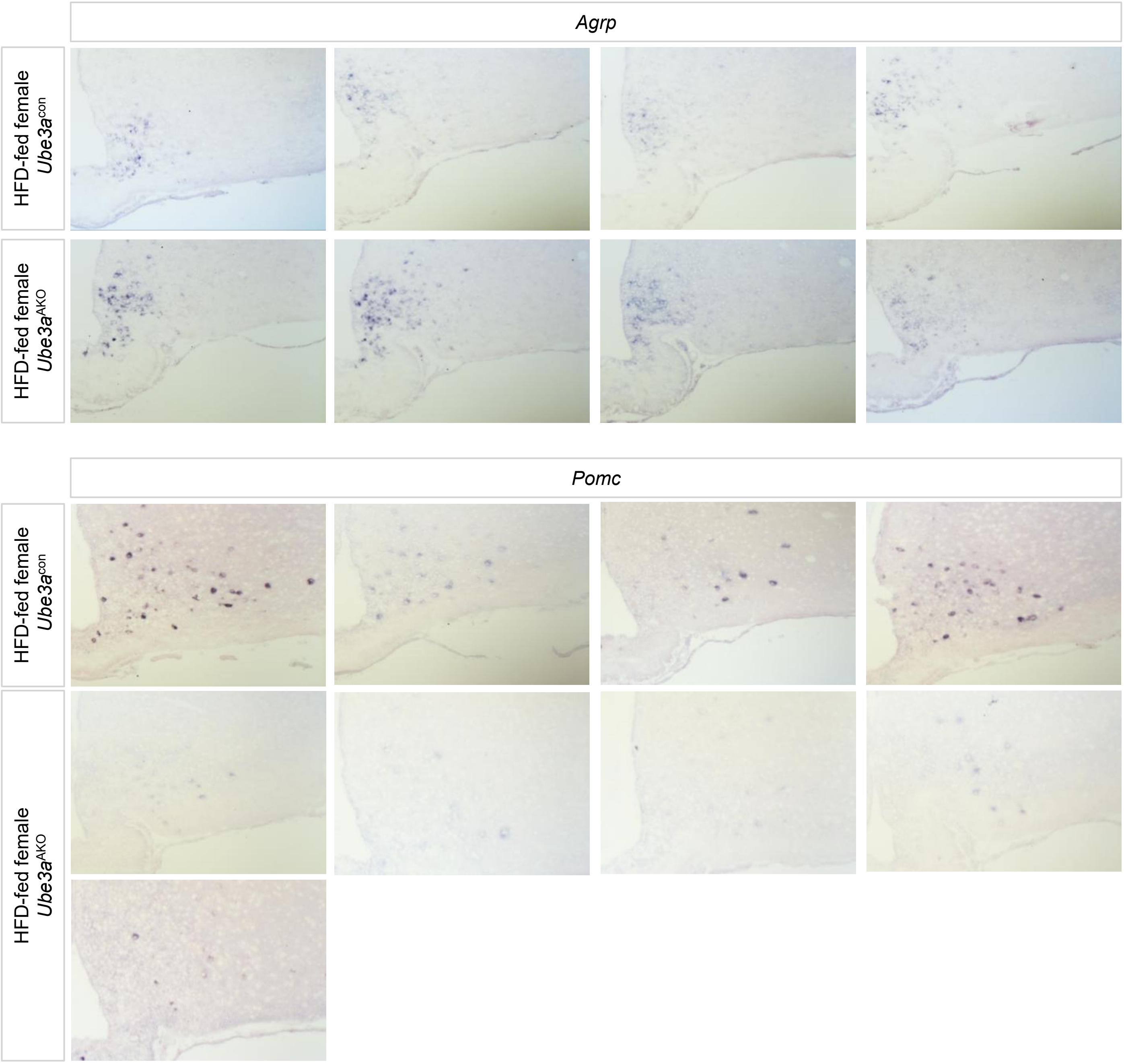

A representative image from each mouse is included.

Source Data Figure 4b.

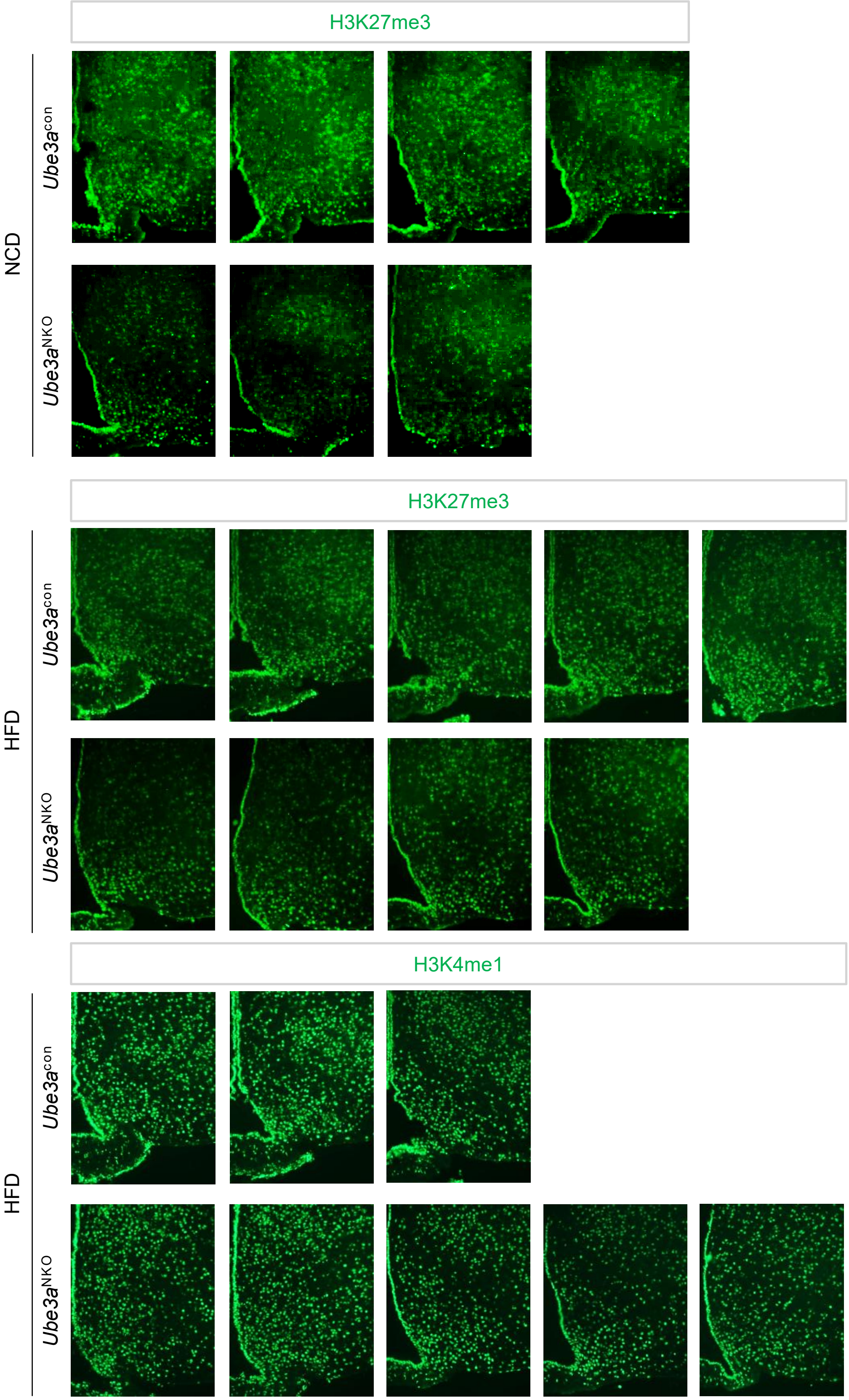

A representative image from each mouse is included.

Source Data Figure 4b.

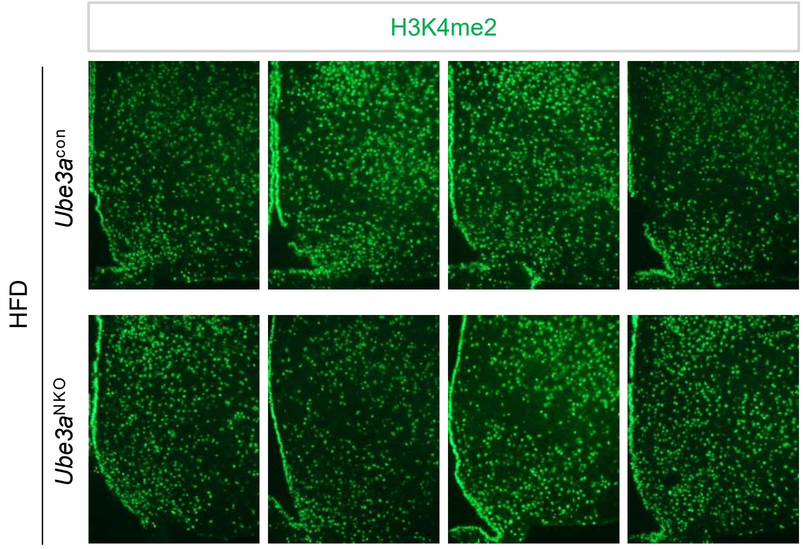

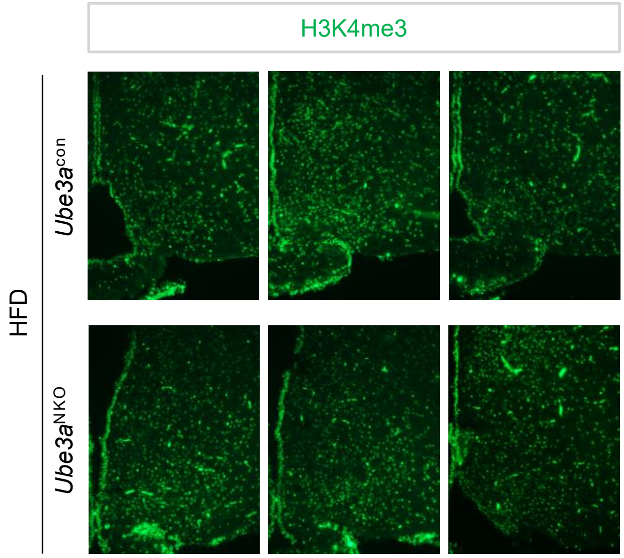

A representative image from each mouse is included.

Source Data Figure 4d.

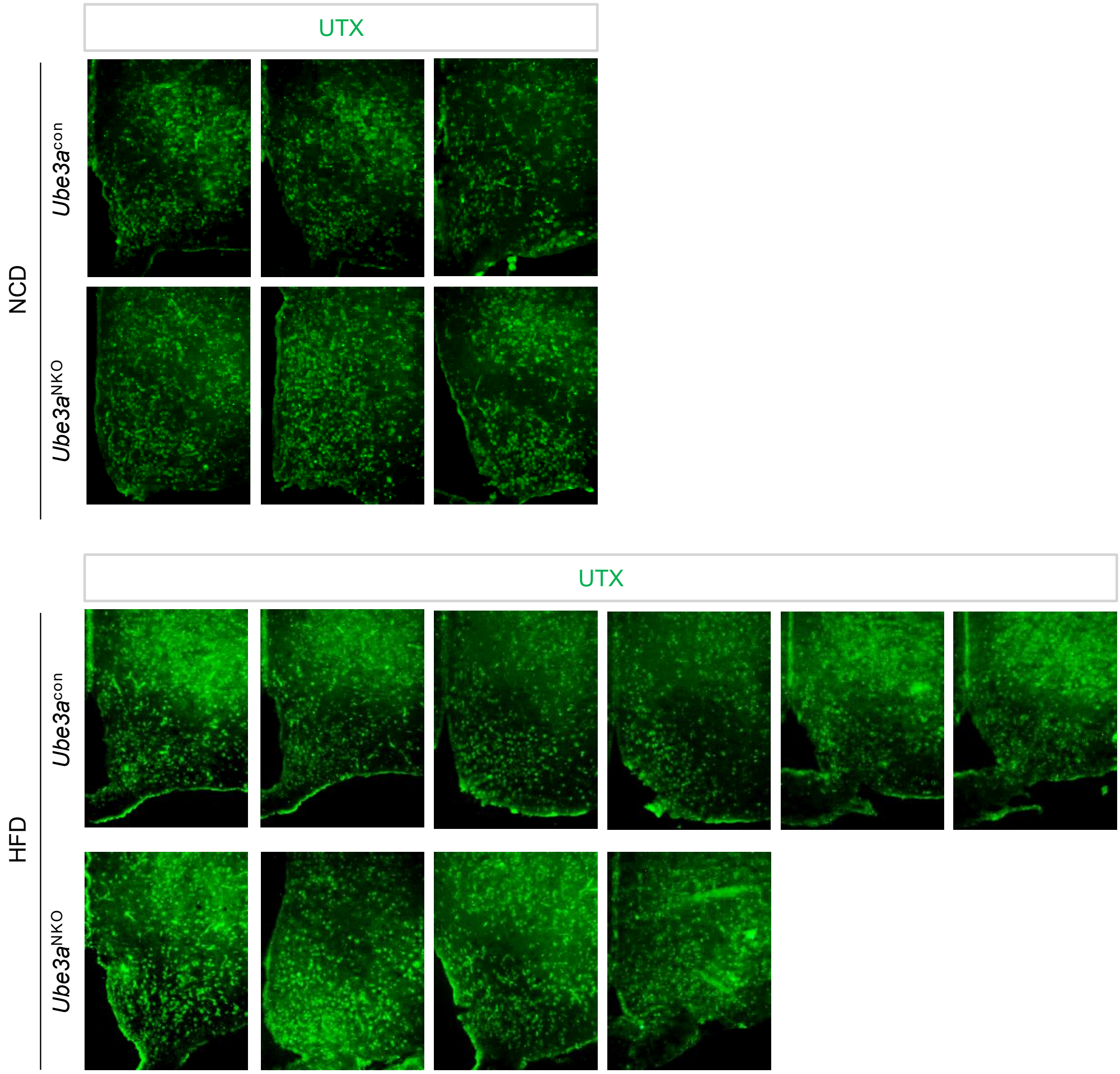

A representative image from each mouse is included.

Source Data Figure 5j.

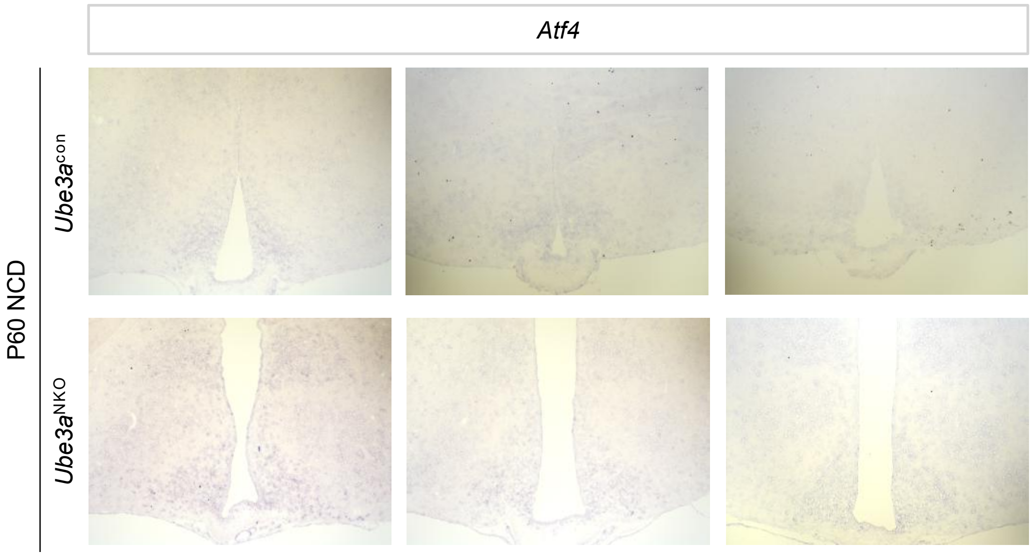

A representative image from each mouse is included.

Source Data Figure 7f.

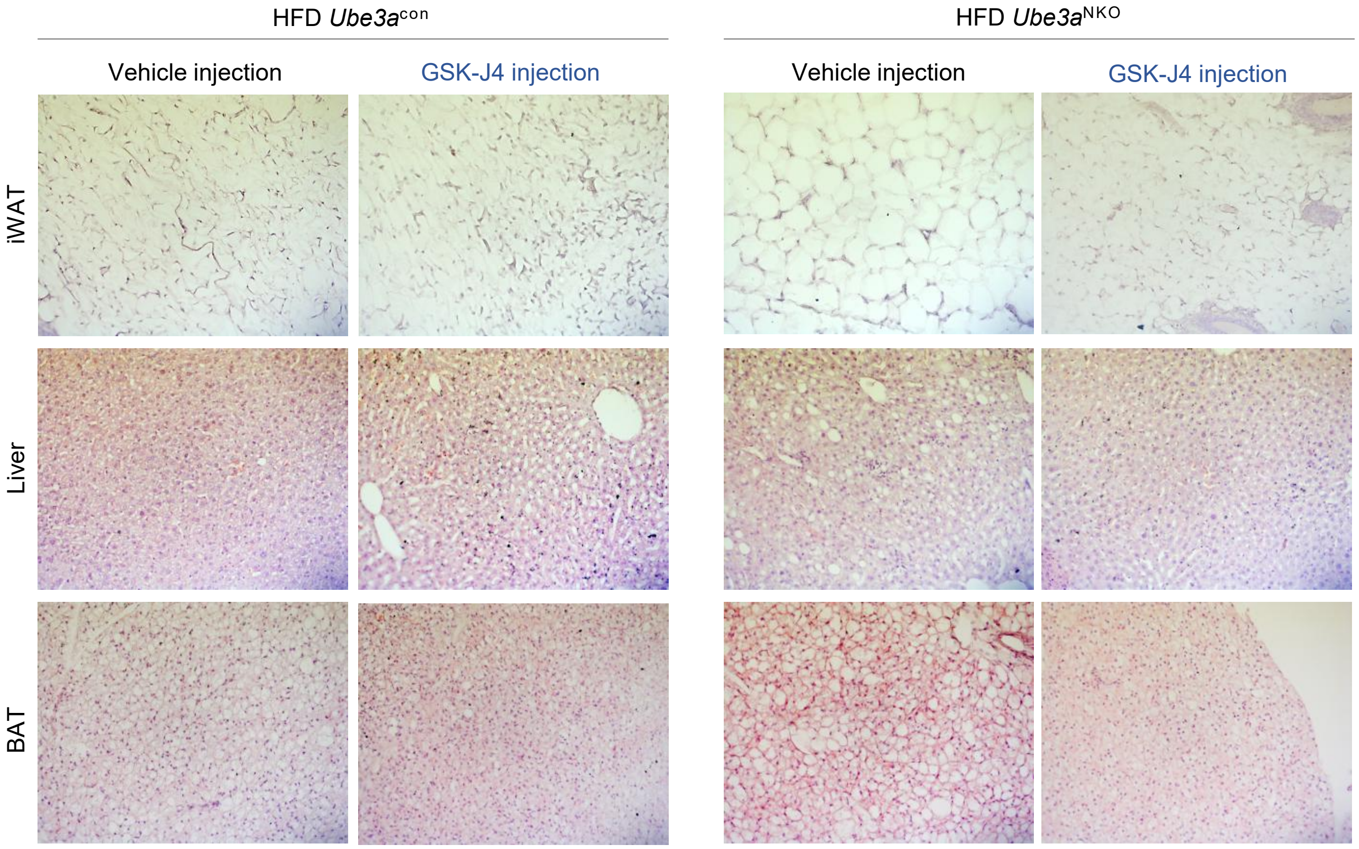

A representative image from each mouse is included.

Source Data Figure 7g.

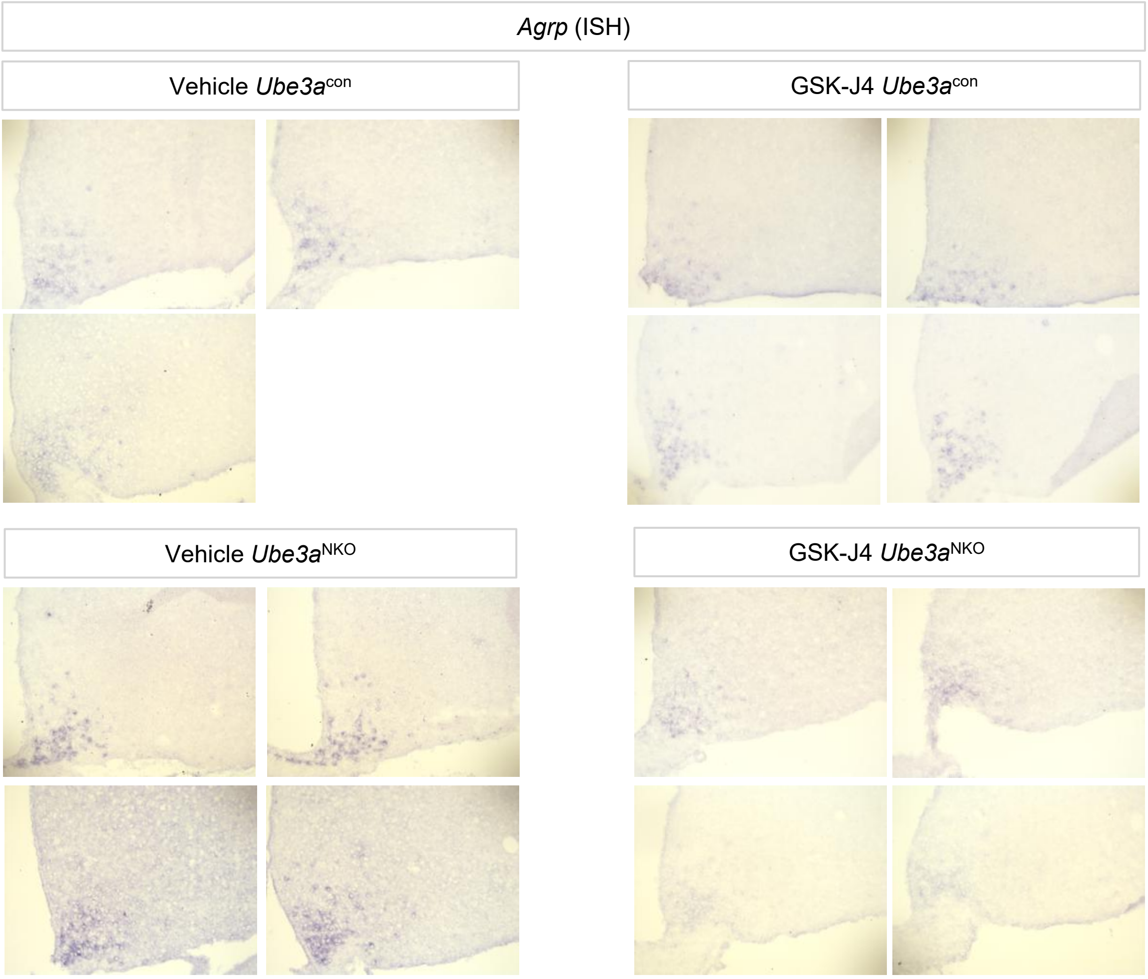

A representative image from each mouse is included.

Source Data Figure 7g.

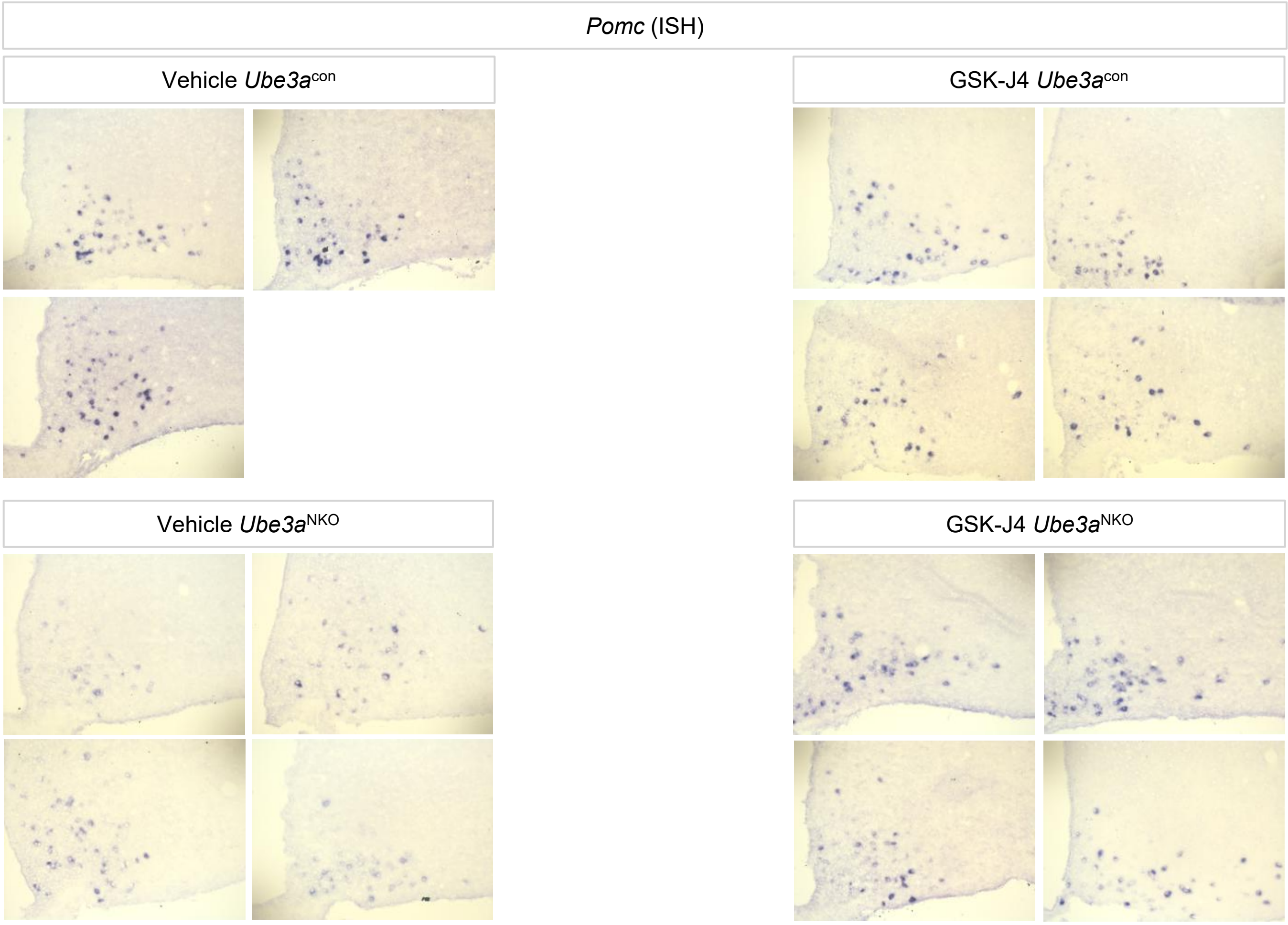

A representative image from each mouse is included.

Source Data Figure 7h.

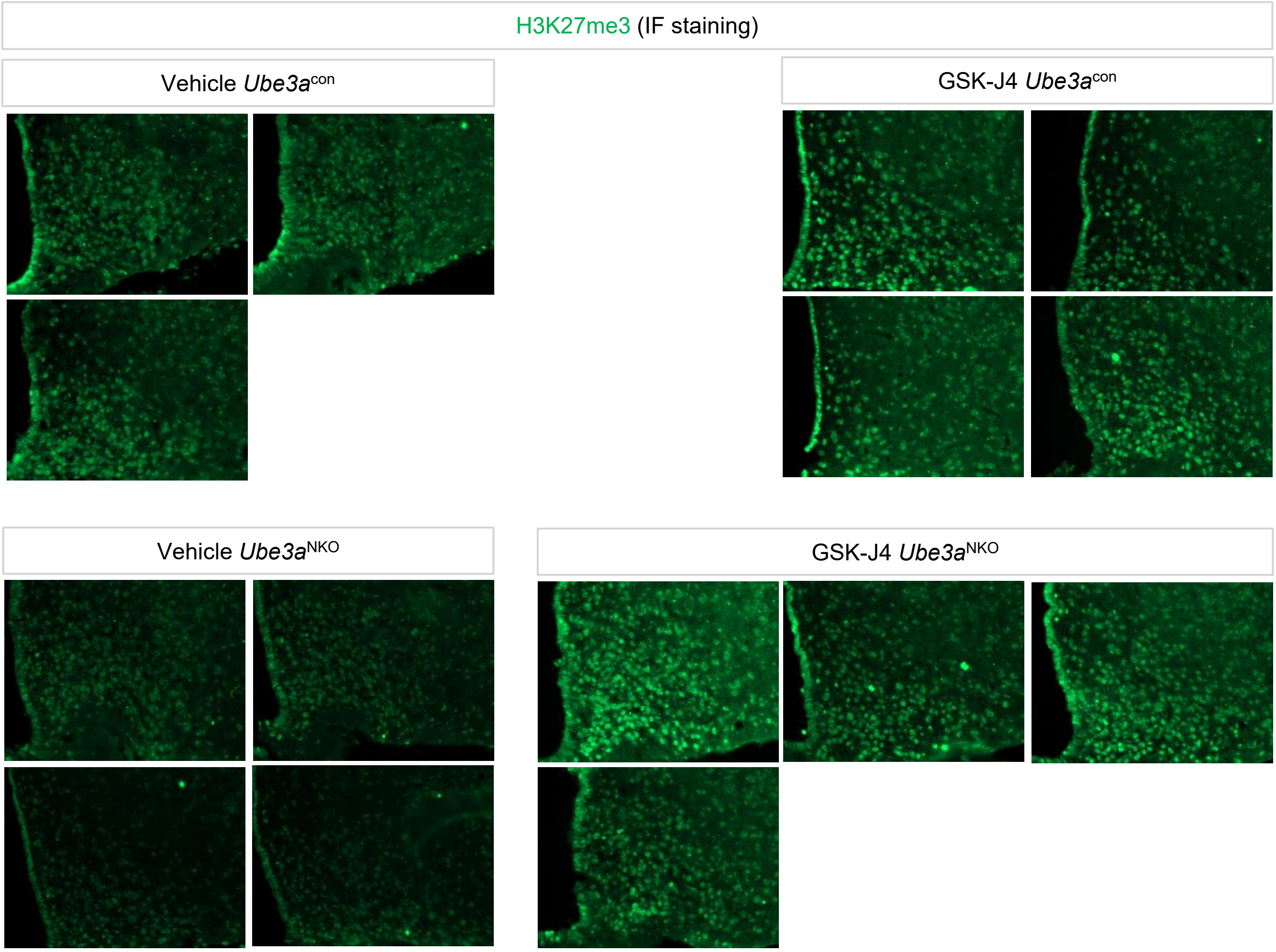

A representative image from each mouse is included.

Source Data Figure 7h.

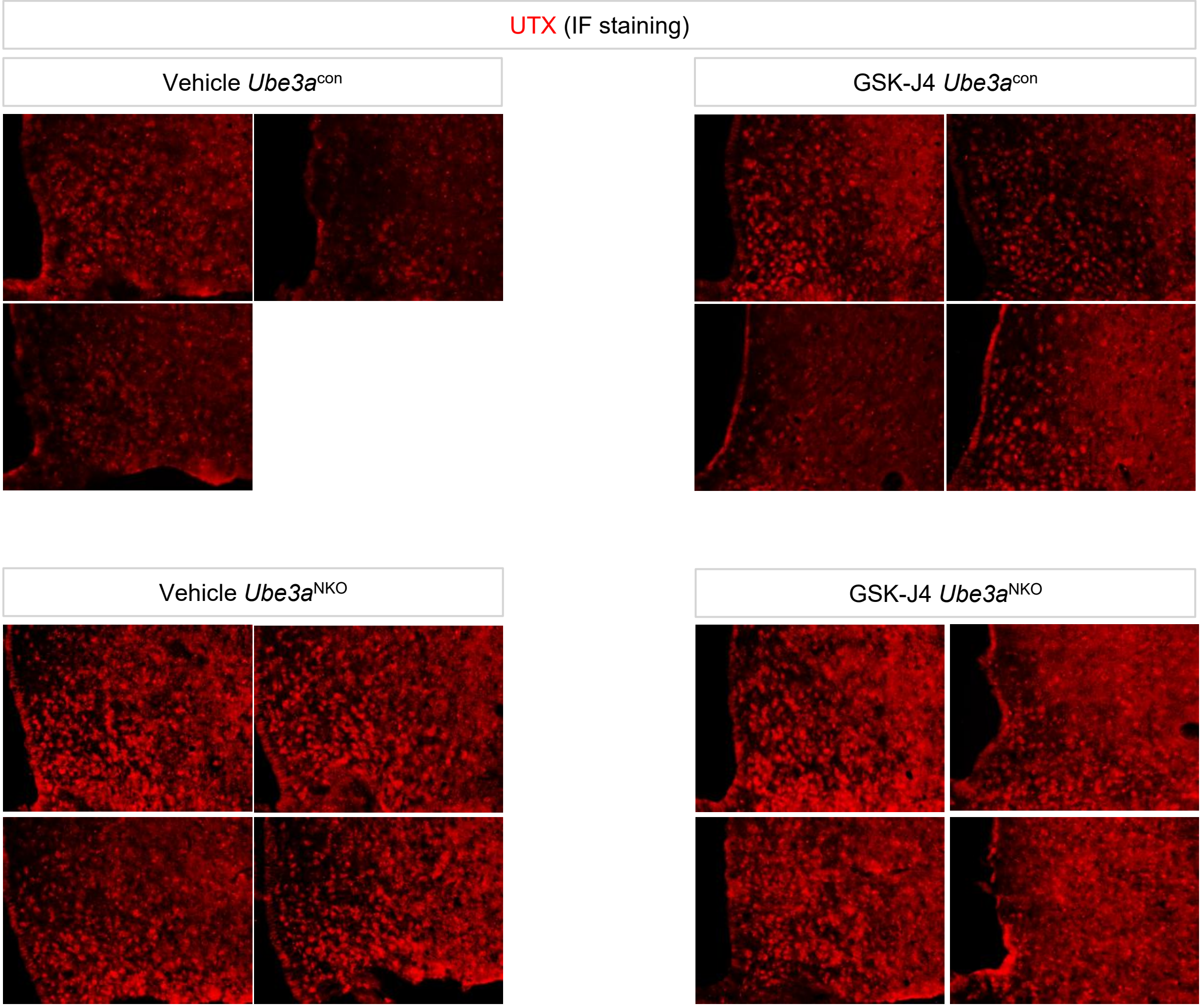

A representative image from each mouse is included.

Source Data Figure 7i.

Pdia3 (ISH)

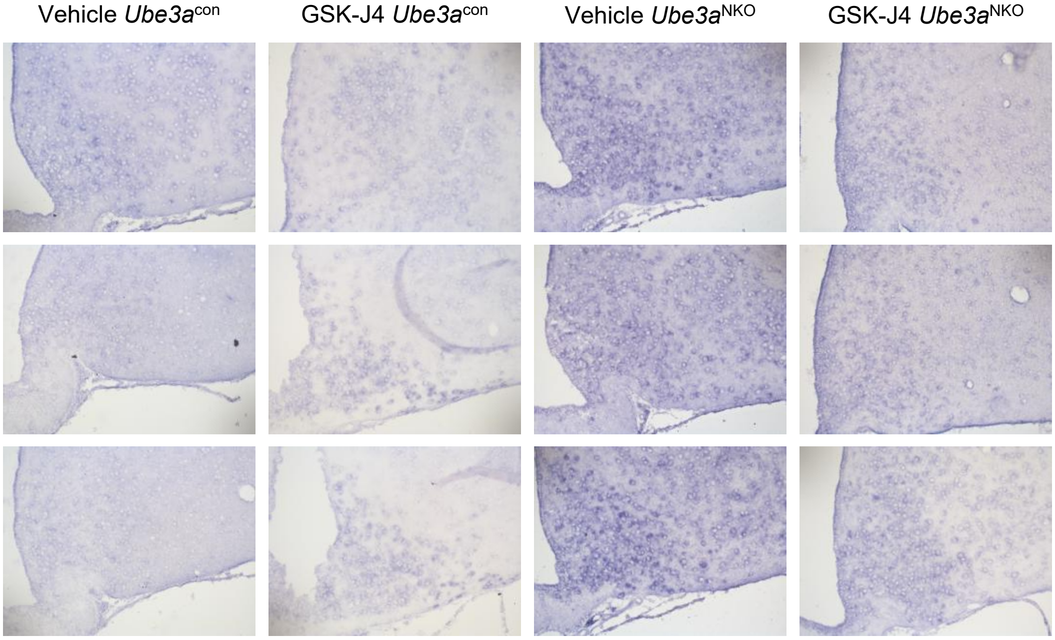

A representative image from each mouse is included.

Source Data Figure 7i.

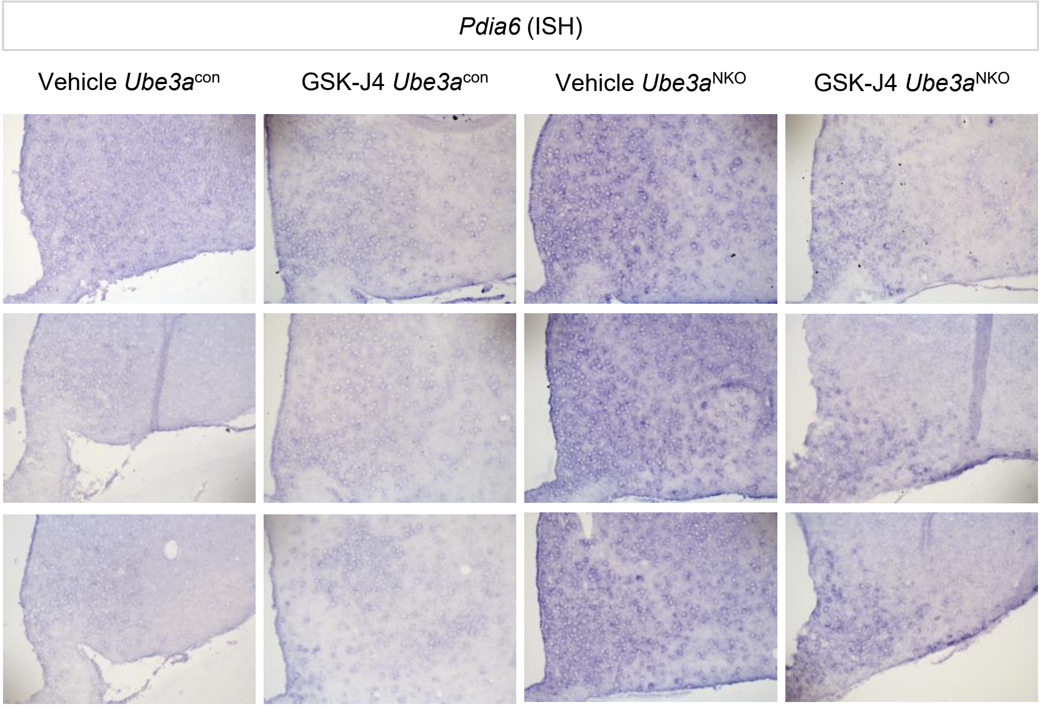

A representative image from each mouse is included.

Source Data Figure S1a.

Male *NPY-hrGfp* P40

91±1% of NPY$^+$ cells = UBE3A$^+$ cells (n=10)

GFP UBE3A

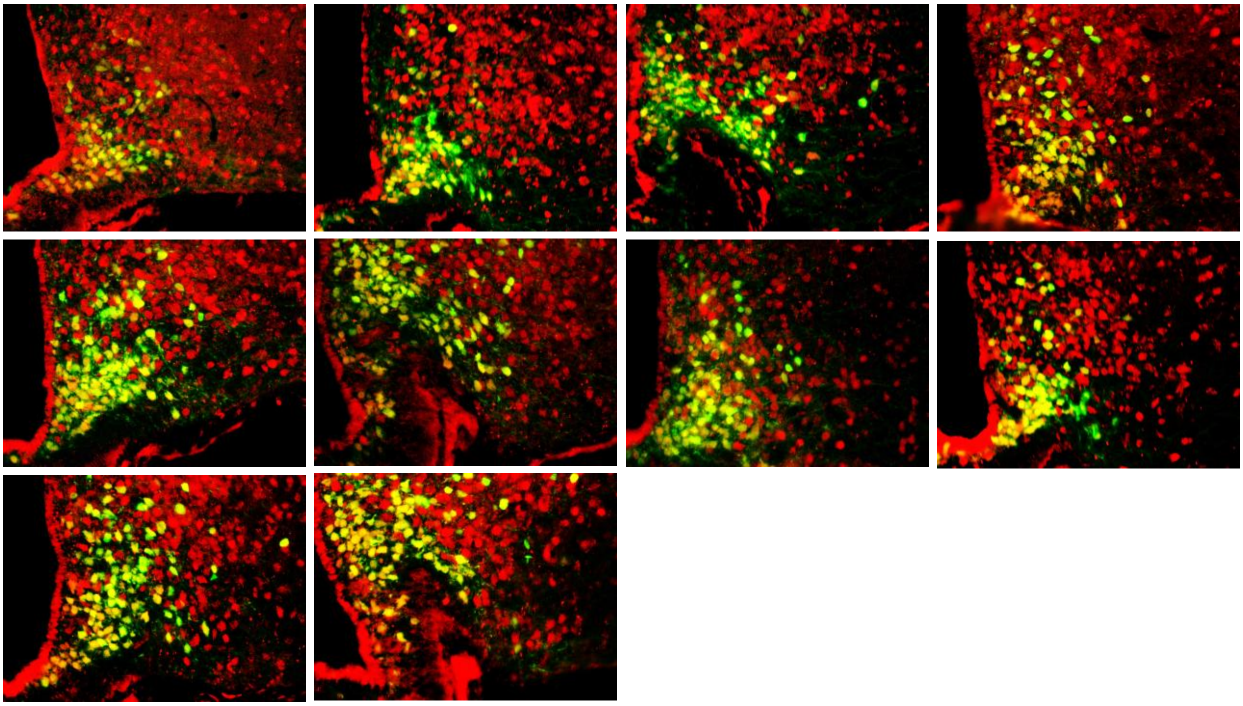

A representative image from each mouse is included.

Source Data Figure S1b.

Male *Pomc-Cre;tdTomato*/+ (P40)

91±2% of POMC⁺ cells = UBE3A⁺ cells (n=12)

UBE3A tdTomato

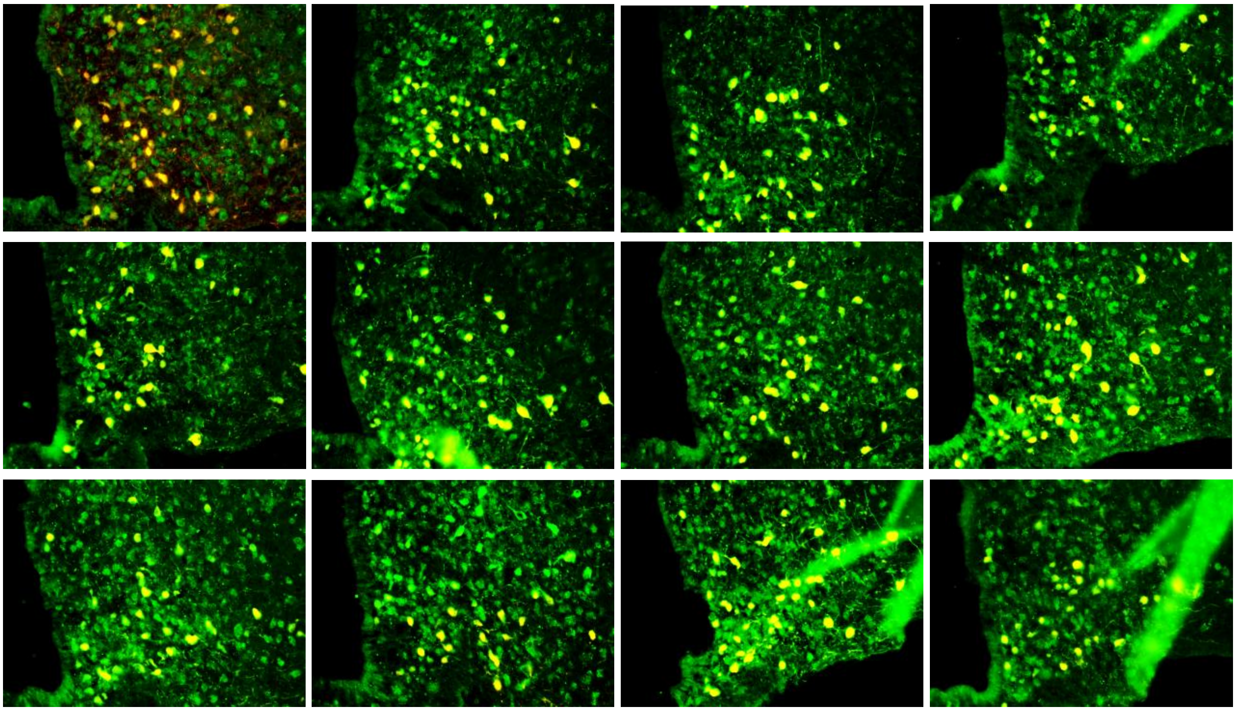

A representative image from each mouse is included.

Source Data Figure S1c.

Male *Ghrh-Cre;tdTomato*/+ (P40)

88±3% of GHRH^{+} cells = UBE3A^{+} cells (n=16)

UBE3A tdTomato

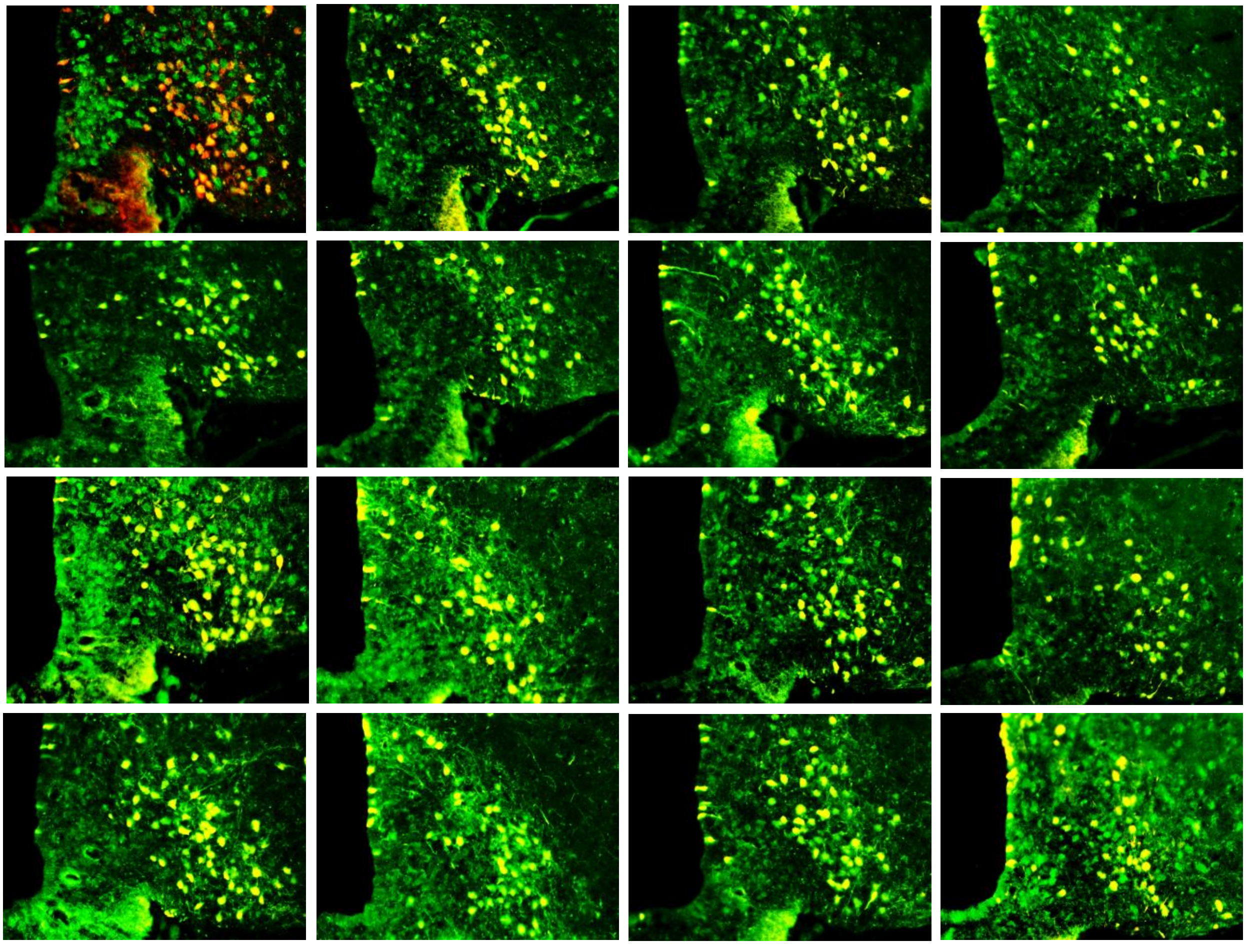

A representative image from each mouse is included.

Source Data Figure S8b.

***Pomc* in ARC region**

***Ube3a* con cherry CNO** | ***Ube3a*-AKO cherry CNO** | ***Ube3a*-AKO hicherry CNO**

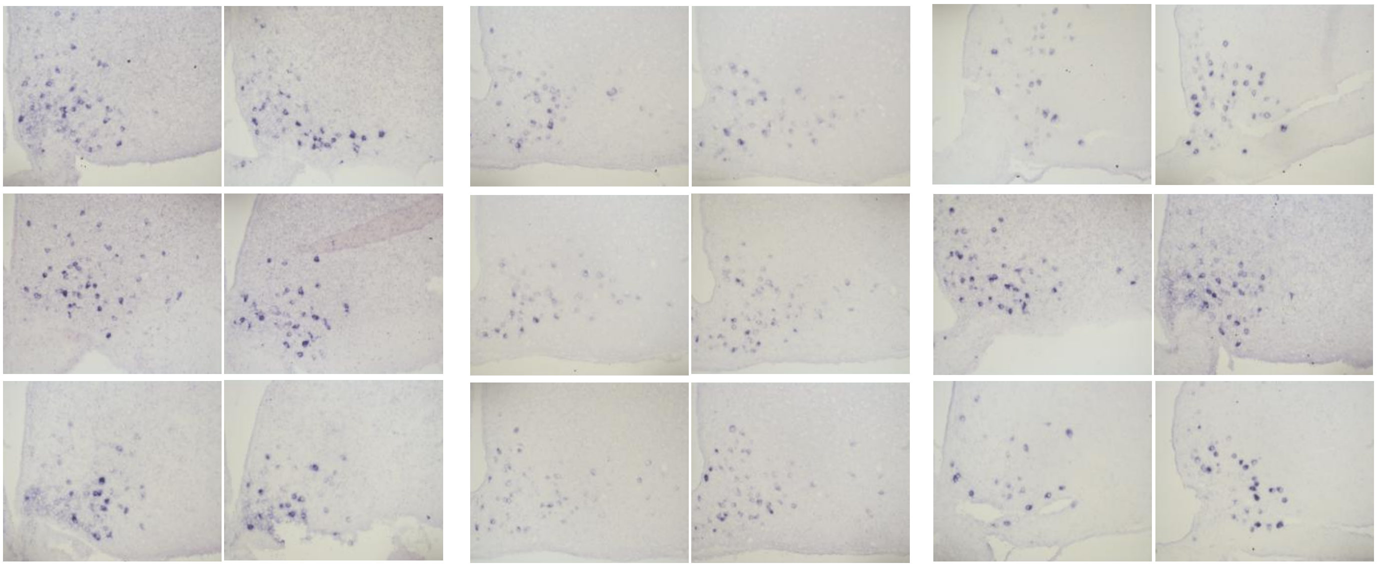

A representative image from each mouse is included.

Source Data Figure S8c.

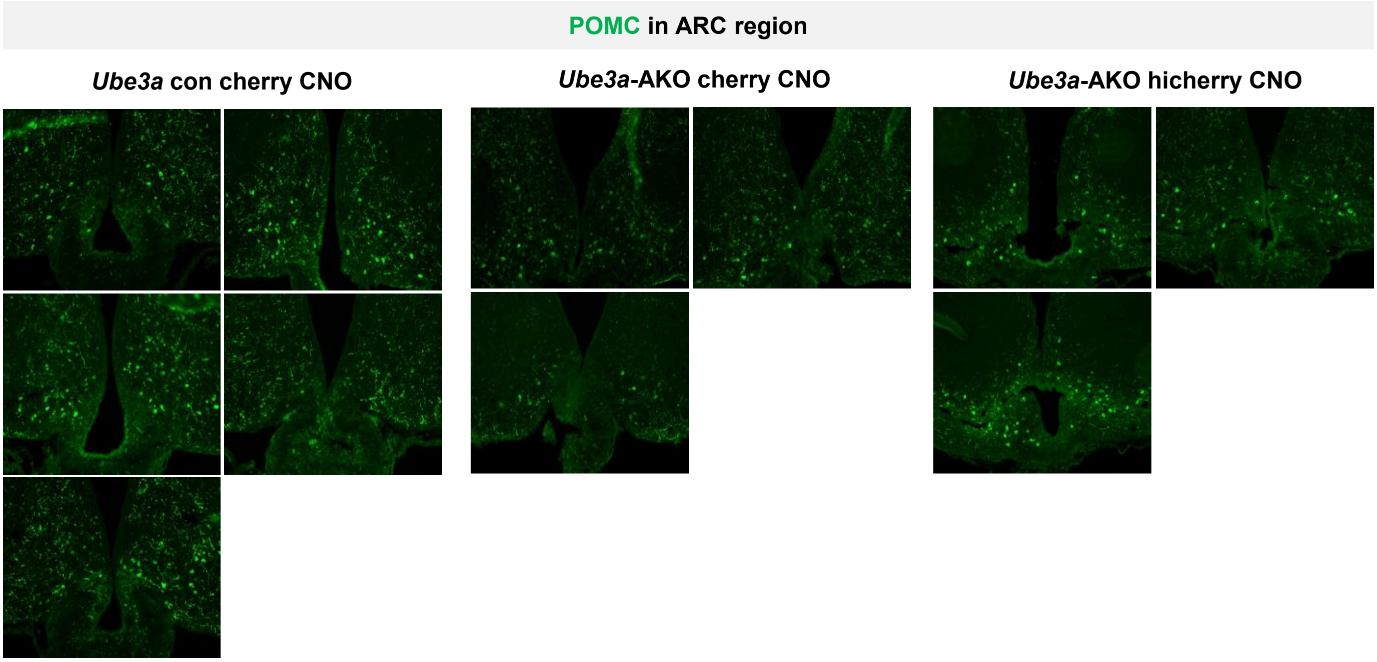

A representative image from each mouse is included.

Source Data Figure S8d.

AgRP in ARC region

***Ube3a* con cherry CNO** | ***Ube3a*-AKO cherry CNO** | ***Ube3a*-AKO hicherry CNO**

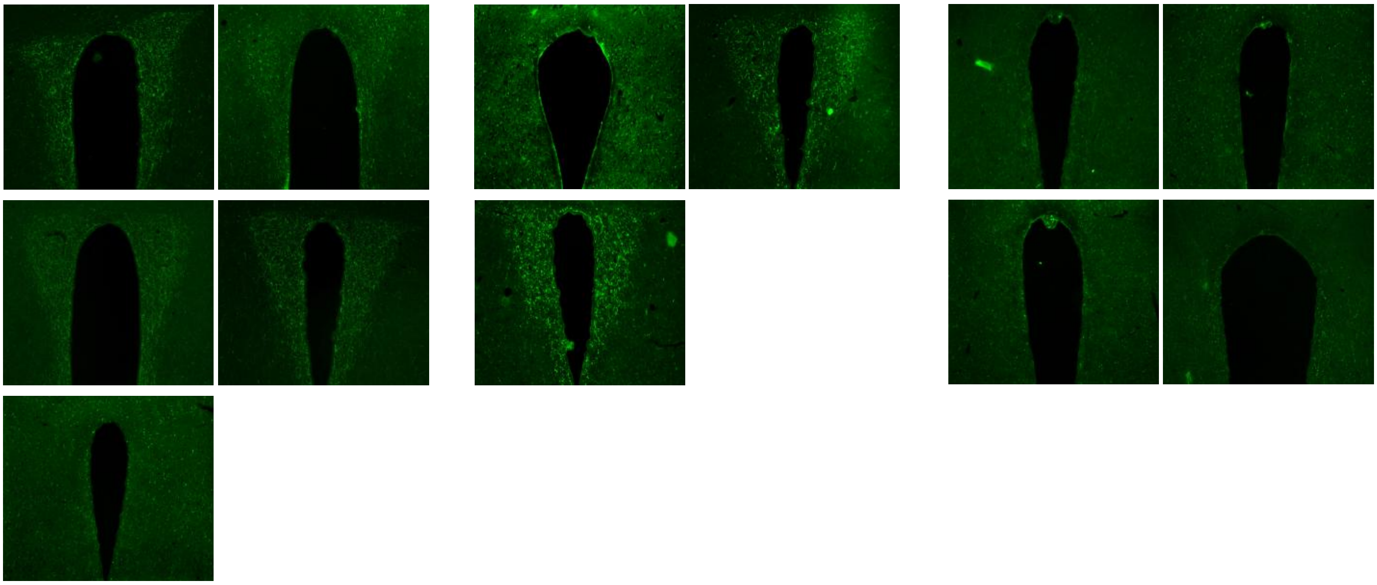

A representative image from each mouse is included.

Source Data Figure S8e.

α-MSH in ARC region

***Ube3a* con cherry CNO** | ***Ube3a*-AKO cherry CNO** | ***Ube3a*-AKO hicherry CNO**

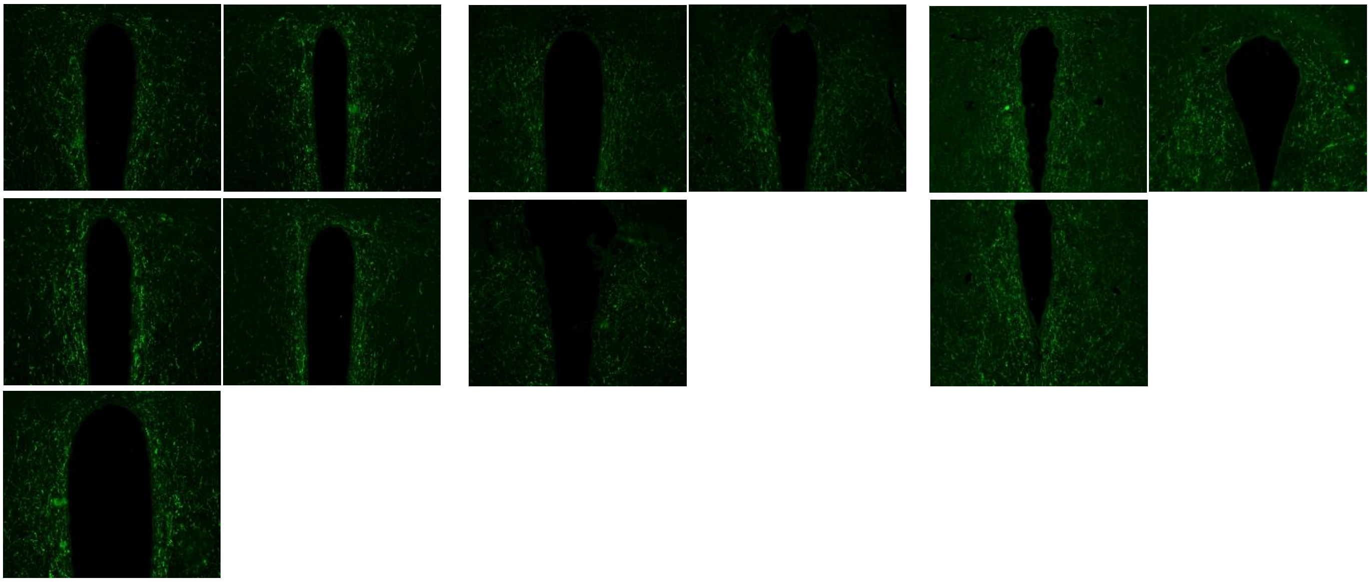

A representative image from each mouse is included.

Source Data Figure S11a.

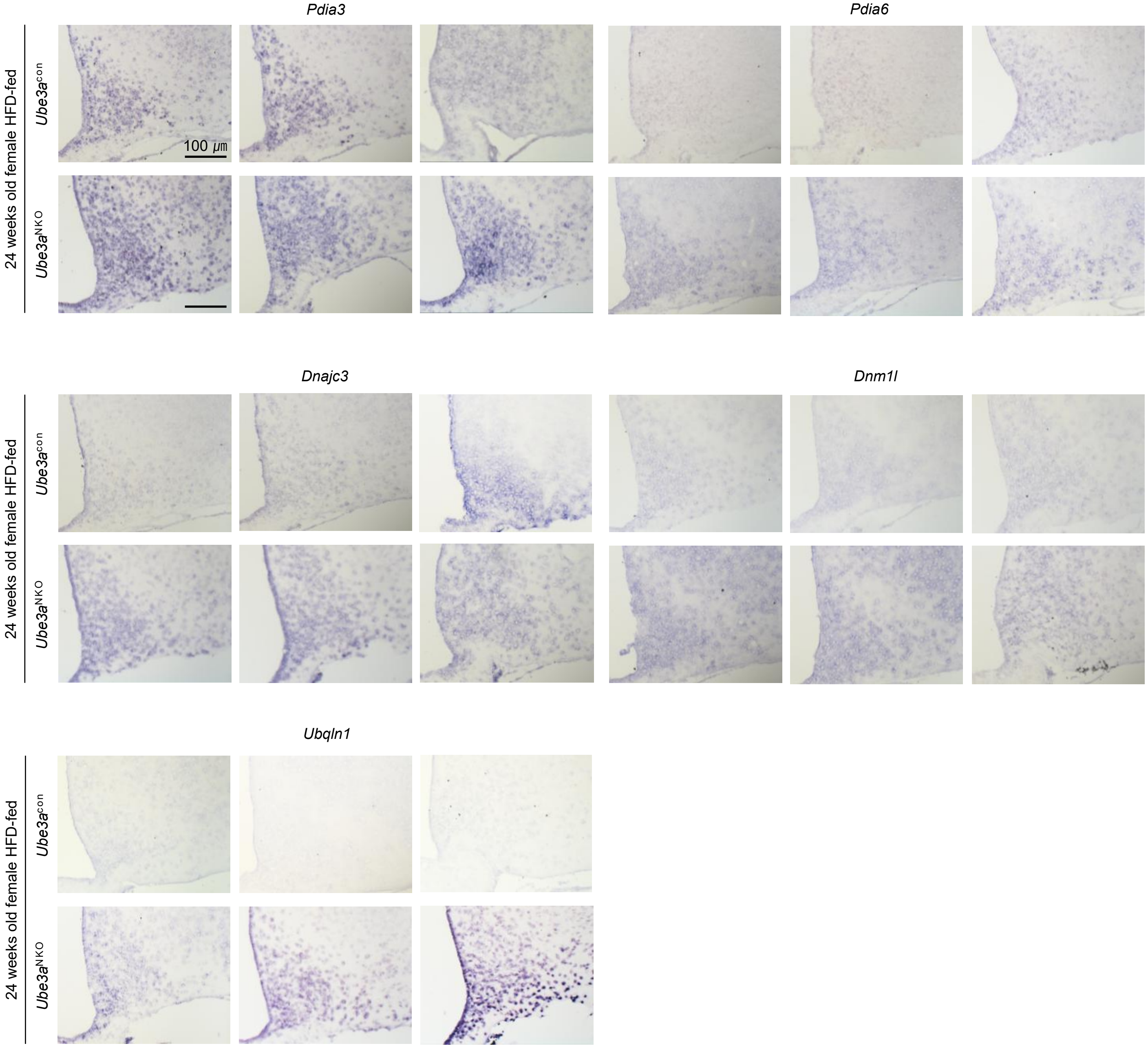

A representative image from each mouse is included.

Source Data Figure S12b.

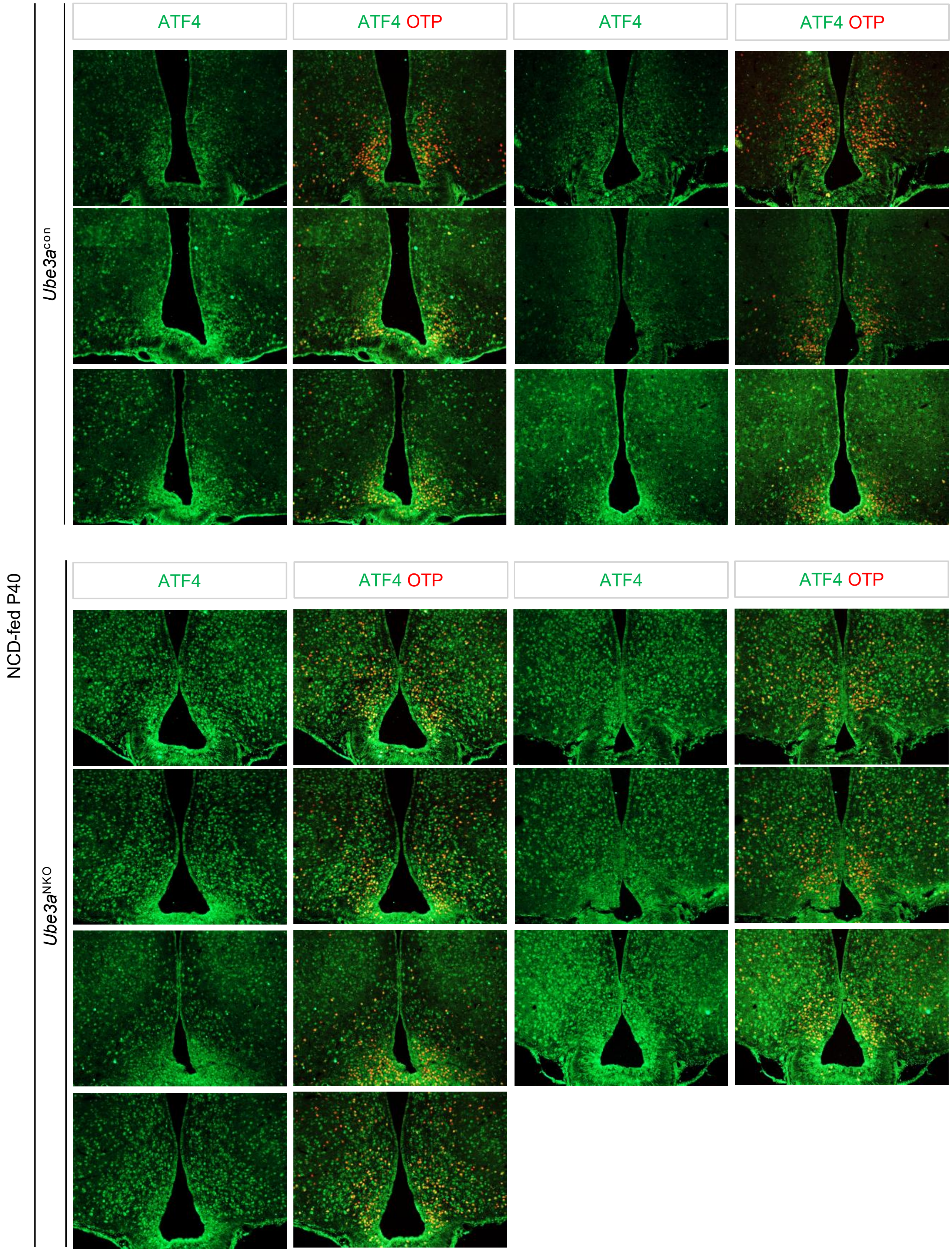

A representative image from each mouse is included.
