# Supplementary material for: A UBE3A‐UTX Proteostasis‐Epigenetic Axis Governs Hypothalamic Energy Homeostasis
Source: Adv Sci (Weinh). 2026 Oct 29:e78567. Online ahead of print. doi: 10.1002/advs.78567 (PMC13720268; doi:10.1002/advs.78567)

Source Data Fig.1d

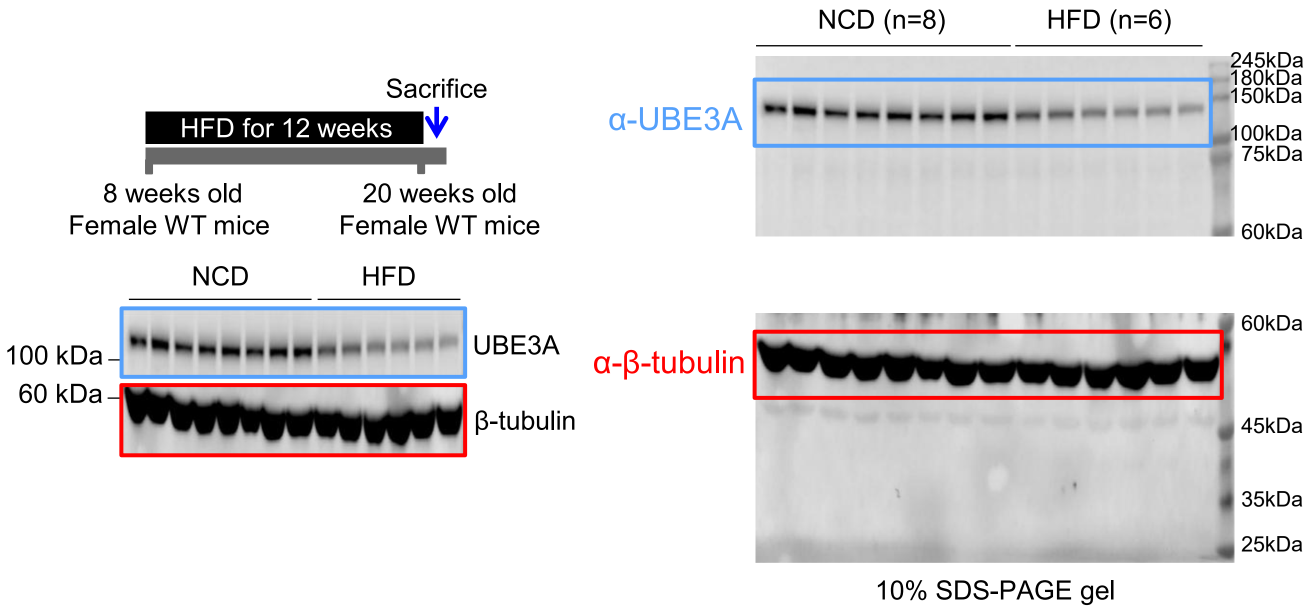

Source Data Fig.1f

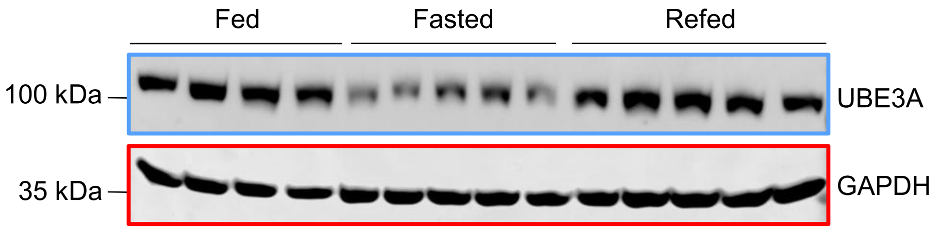

8 weeks old female hypothalamic lysates
Fed: NCD-fed WT (Orient B6) (n=4)
Fasted: Fasted for 24-h WT (Orient B6) (n=5)
Refed: Refed for 6-h WT (Orient B6) (n=5)

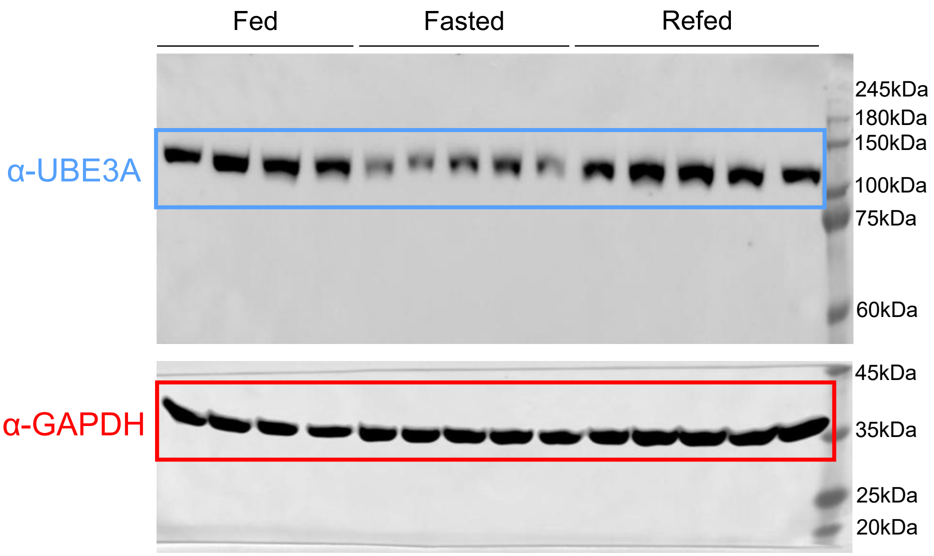

Source Data Fig.4a

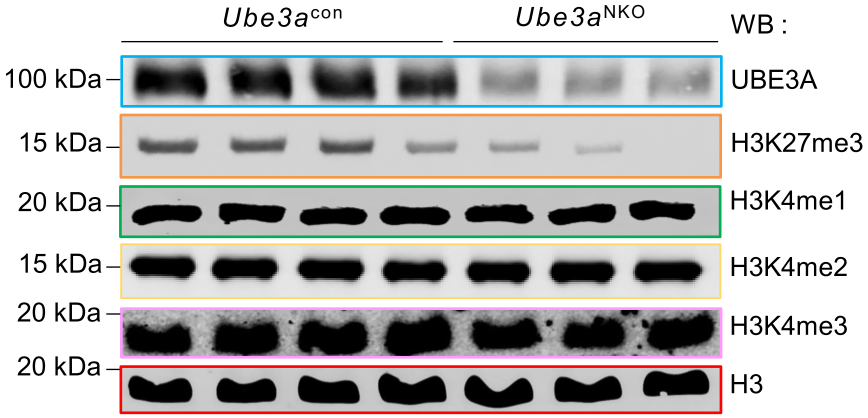

2-month-old female hypothalamic lysates
NCD-fed *Ube3a*^{con} (n=4)
NCD-fed *Ube3a*^{NKO} (n=3)

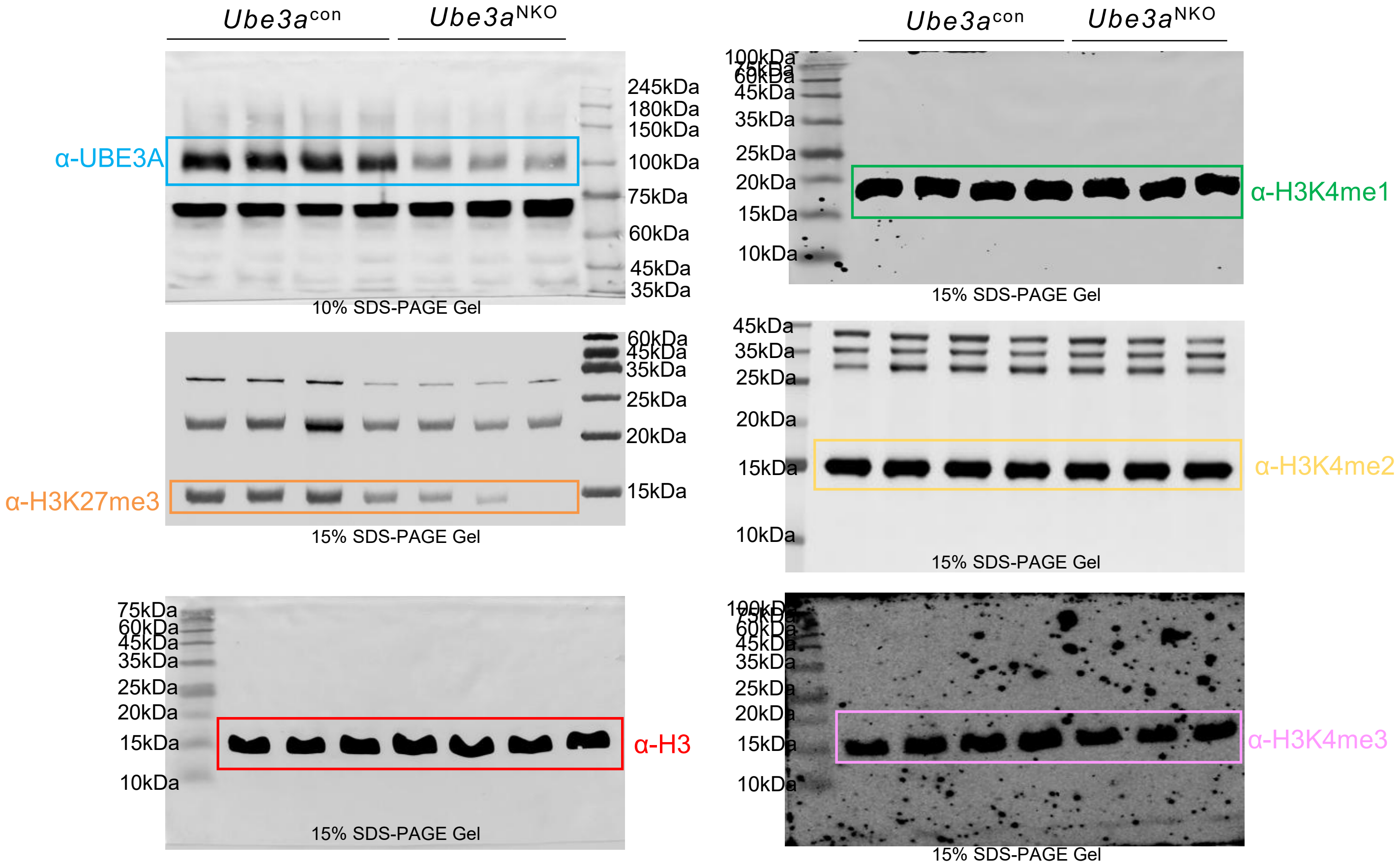

Source Data Fig.4c

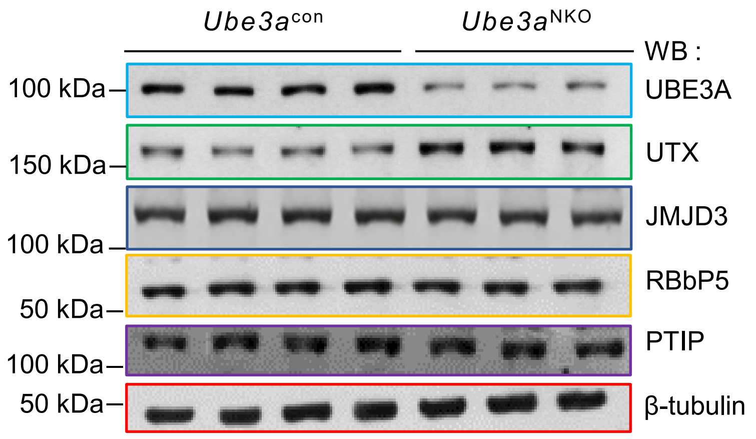

2-month-old female hypothalamic lysates
NCD-fed *Ube3a*^con (WT) (n=4)
NCD-fed *Ube3a*^NKO (cKO) (n=3)

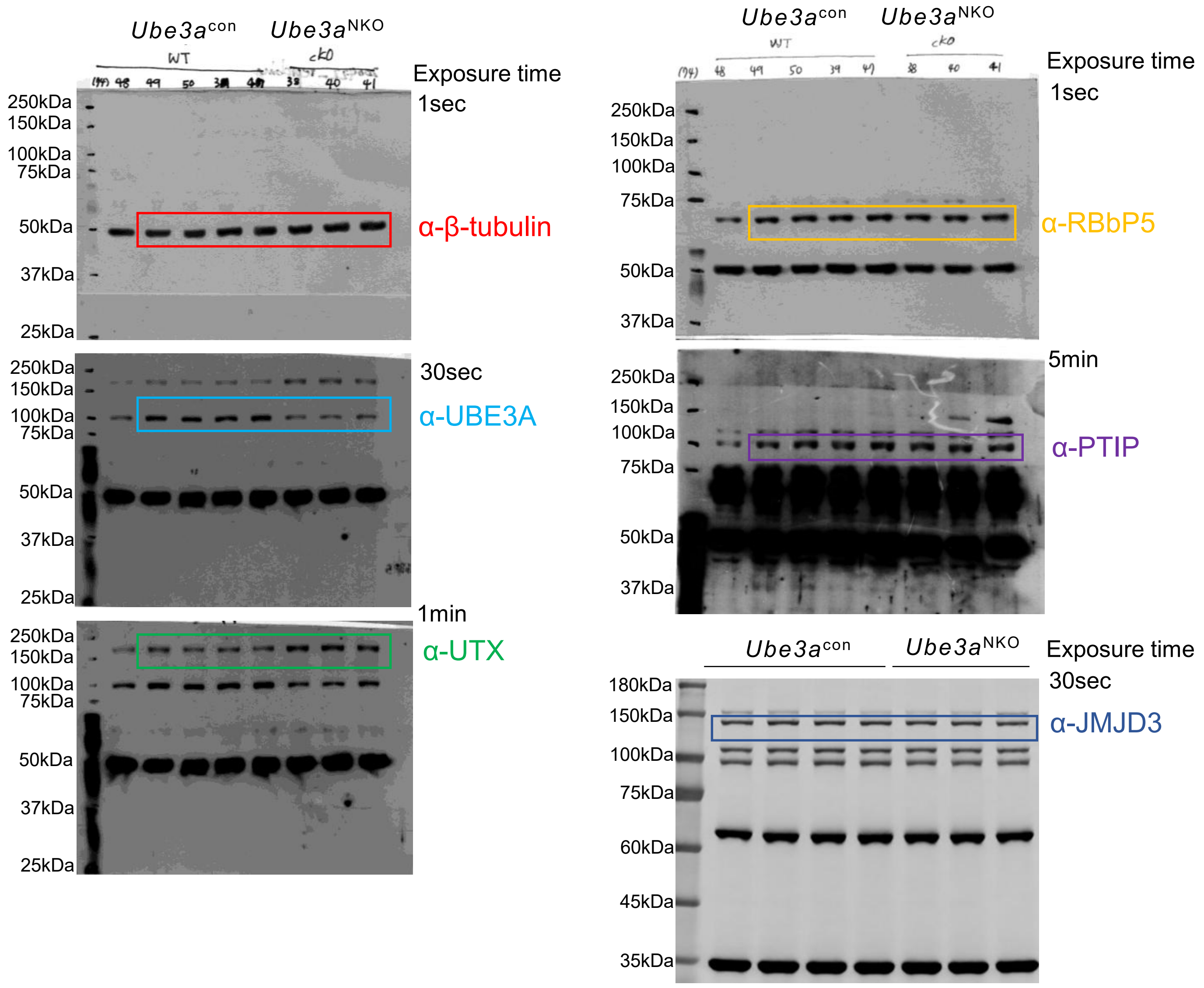

Source Data Fig.4e

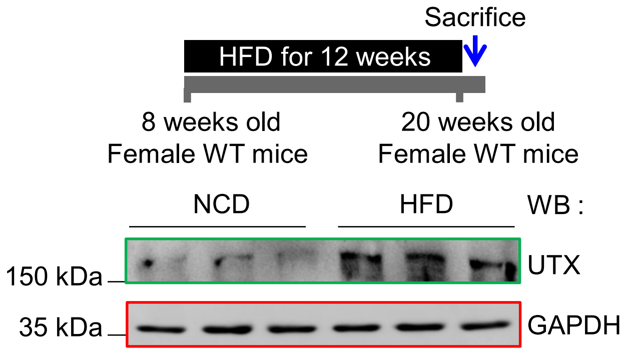

20-week-old female hypothalamic lysates
#1~#3: NCD-fed WT (Orient B6) (n=3)
#1~#3: HFD-fed for 12 weeks WT (Orient B6) (n=3)

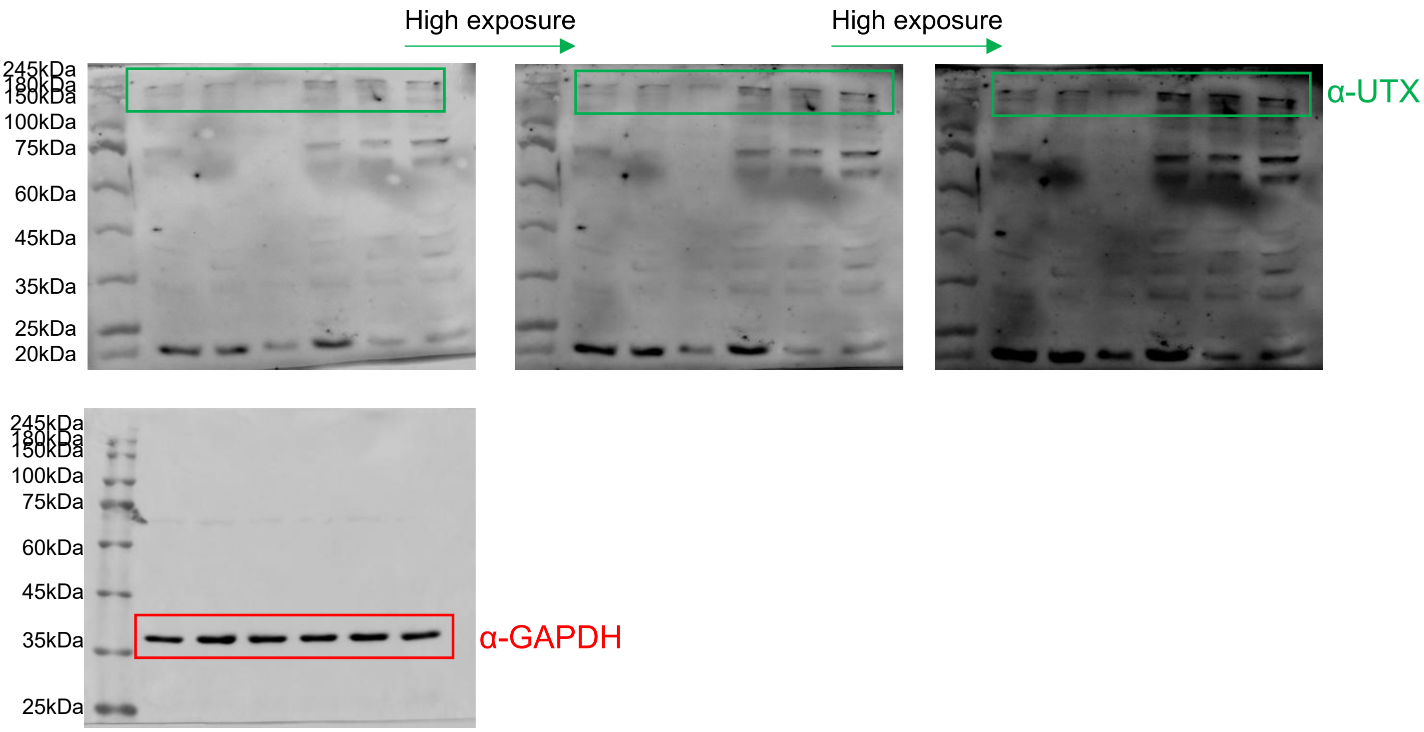

Source Data Fig.4g

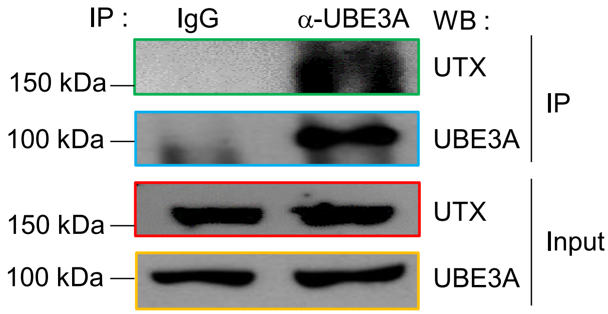

8-month-old female hypothalamic lysates
Antibody for co-immunoprecipitation: α-UBE3A(E6AP) (rb host, 1mg/ml) or IgG (rb host, 1mg/ml)
Primary antibody for western blotting: α-UTX (rb host, 1:2k)
Primary antibody for western blotting: α-UBE3A (rb host, 1:1k)

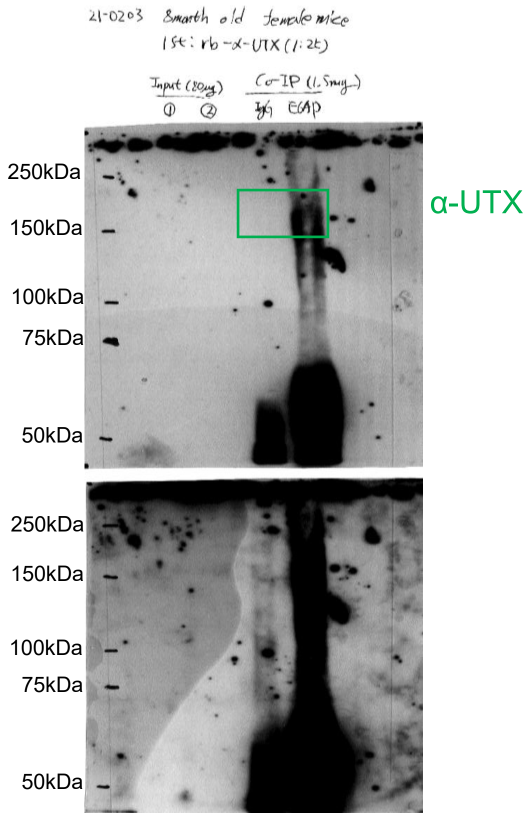

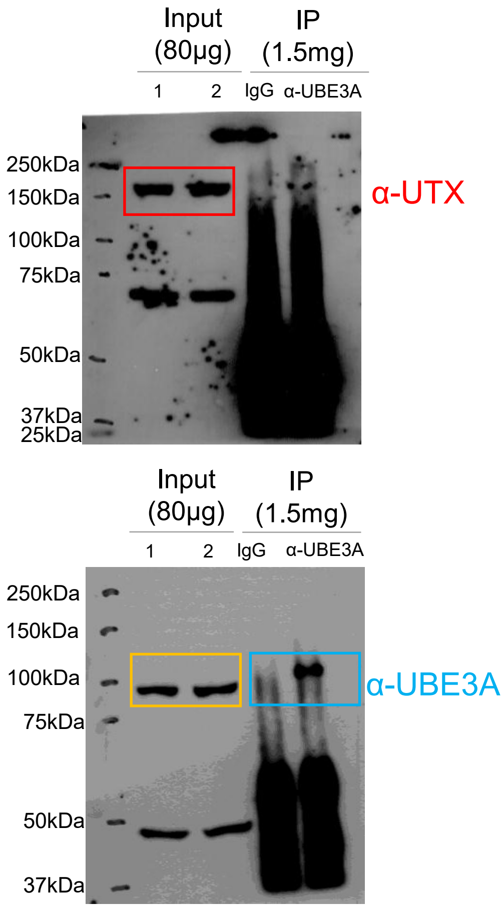

IP: protein lysates (1.5mg)
Input: protein lysates (80μg)

Source Data Fig.4h

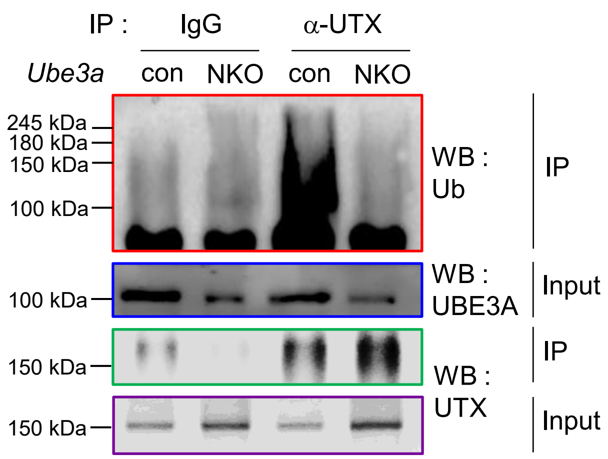

2-month-old NCD-fed female *Ube3a*^{con} and *Ube3a*^{NKO} mice hypothalamic lysates
Antibody for co-immunoprecipitation: α-UTX (rb host, 1mg/ml) or IgG (rb host, 1mg/ml)
Primary antibody for western blotting: α-Ub (rb host, 1:1k), a-UBE3A (rb host, 1:1k), a-UTX (rb host, 1:1k)

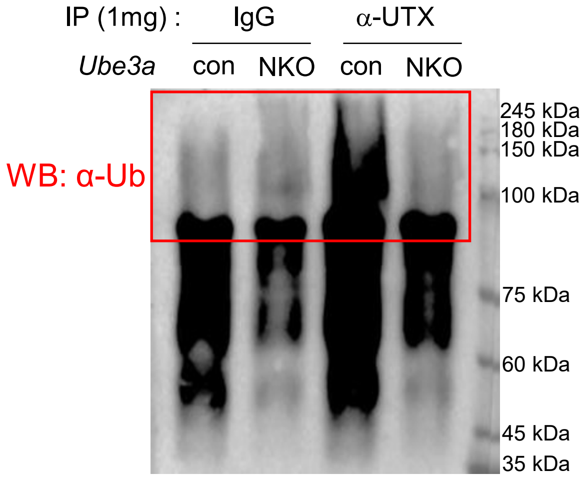

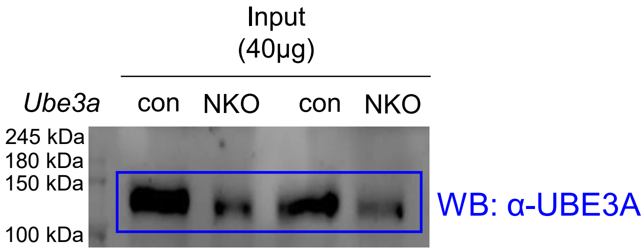

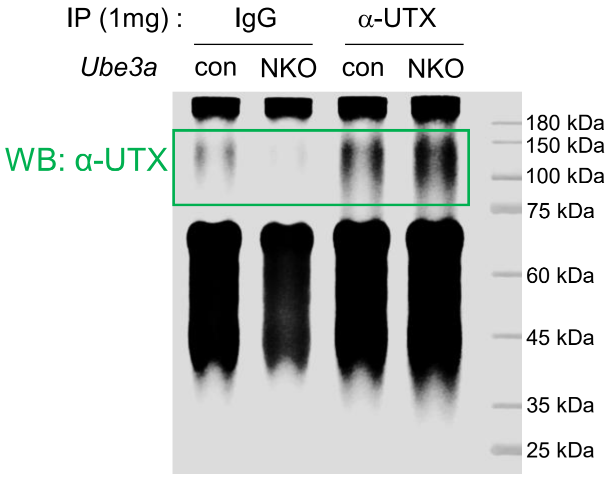

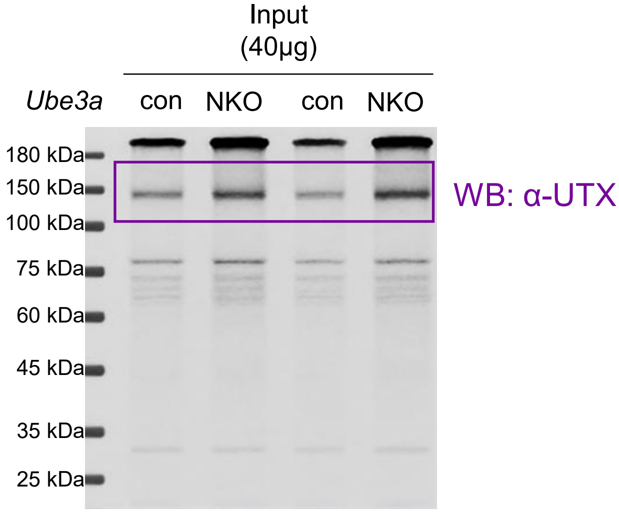

IP: protein lysates (1mg)
Input: protein lysates (40μg)

Source Data Fig.4i

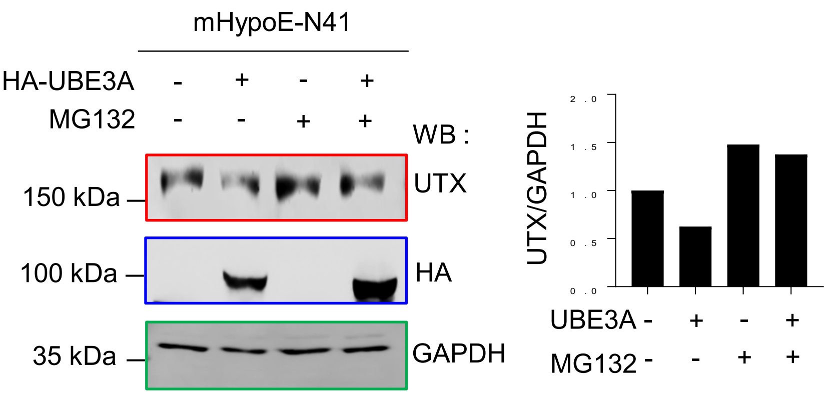

mHypoE-N41 lysates co-expressing HA-UBE3A WT

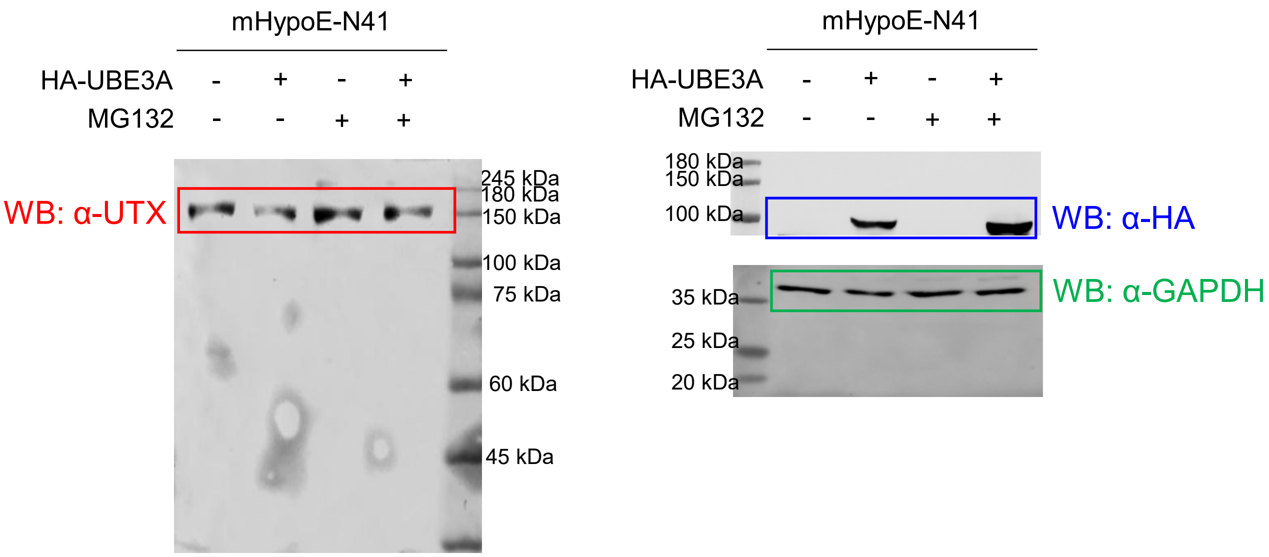

Source Data Fig.5h

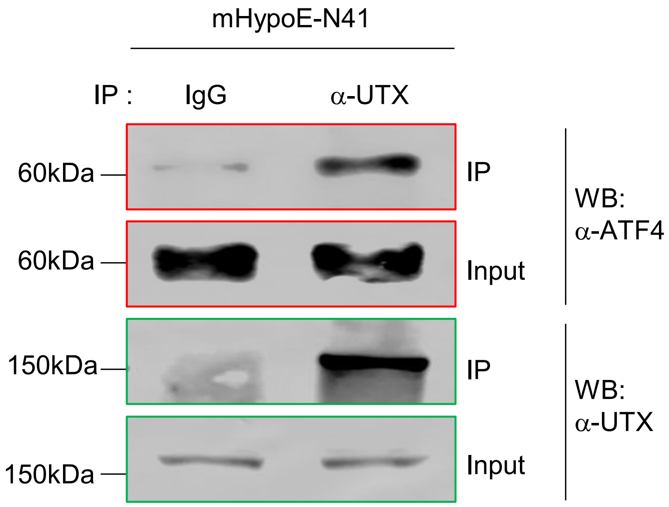

mHypoE-N41 cell lysates
Antibody for co-immunoprecipitation: α-UTX (rb host, 1mg/ml) or IgG (rb host, 1mg/ml)
Primary antibody for western blotting: α-ATF4 (rb host, 1:1k)
Primary antibody for western blotting: α-UTX (rb host, 1:1k)

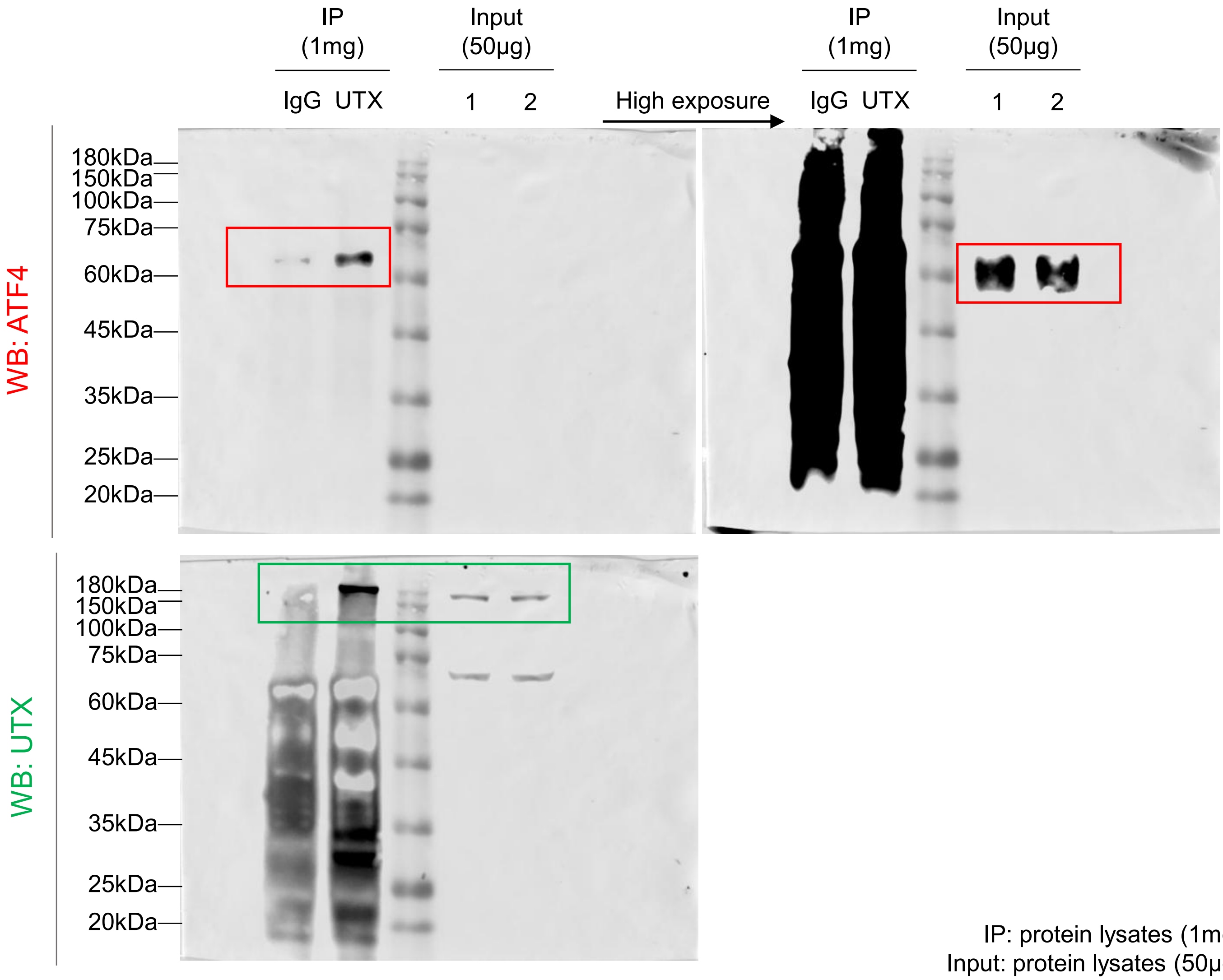

IP: protein lysates (1mg)
Input: protein lysates (50μg)

Source Data Supplementary Fig.9b

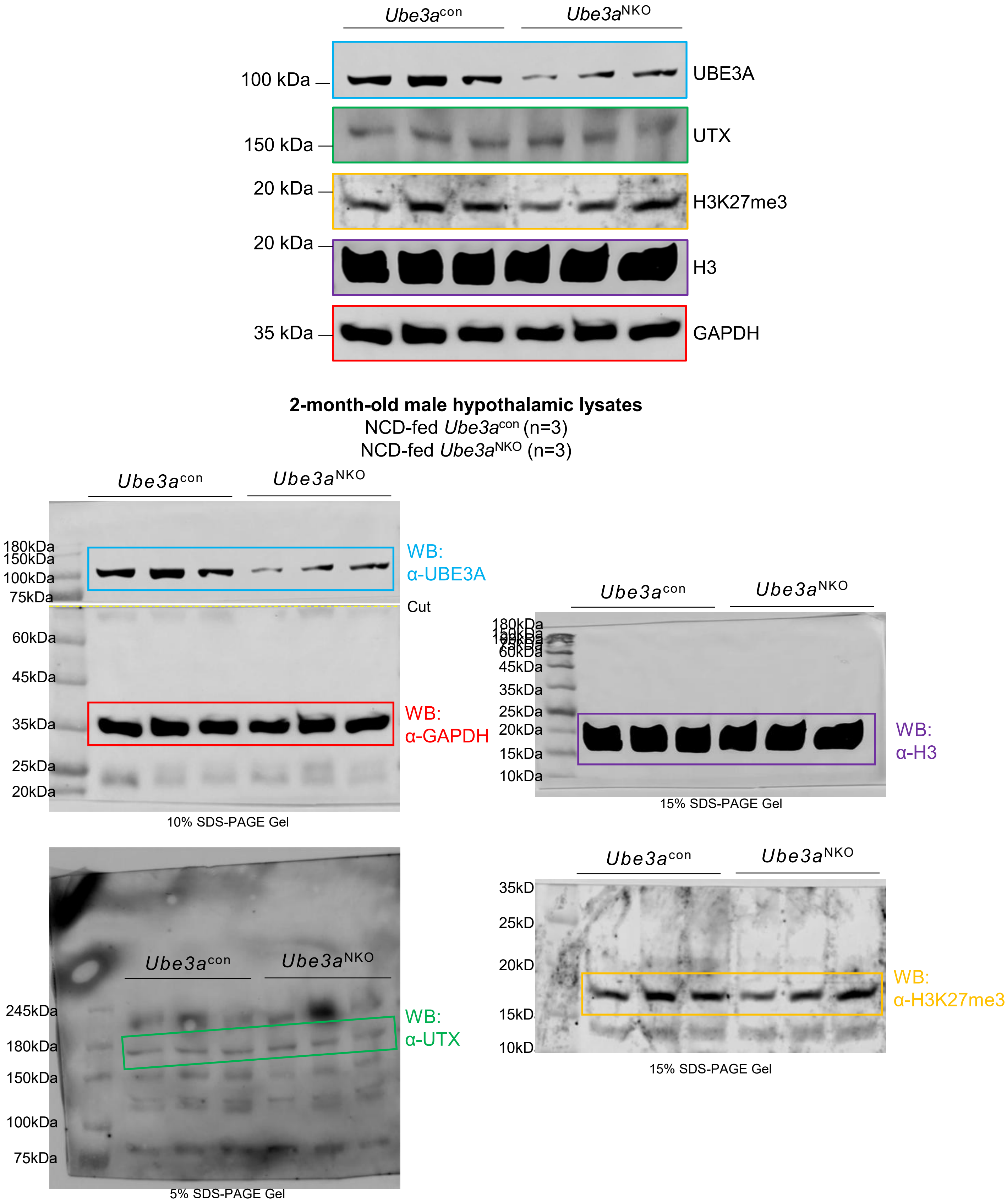

Source Data Supplementary Fig.10a

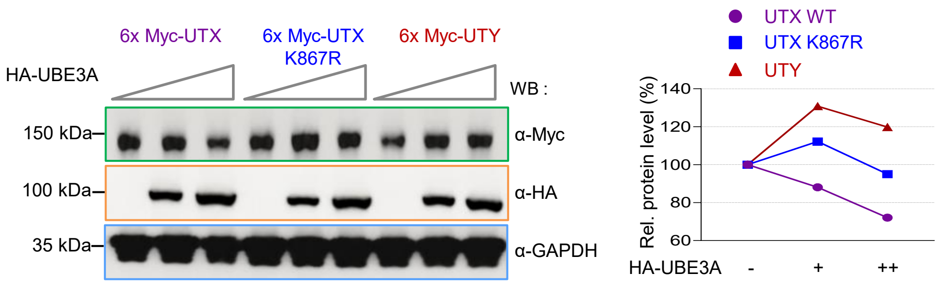

mHypoE-N41 cell lysates co-expressing 6x Myc-UTX, 6x Myc-UTX K867R, 6x Myc-UTY, and HA-UBE3A

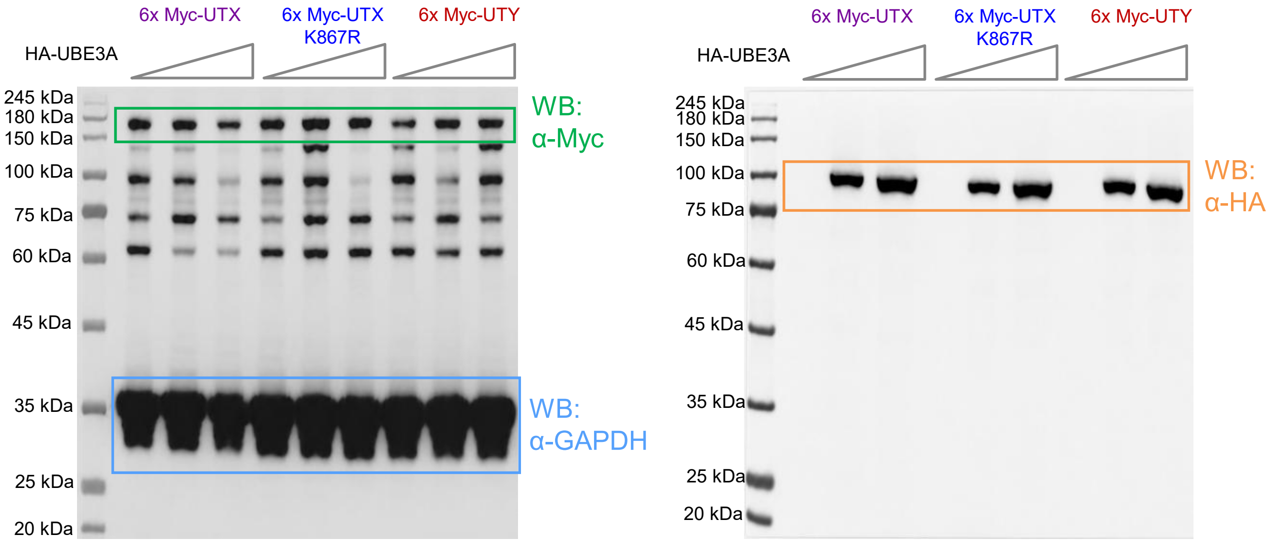

Source Data Supplementary Fig.10b

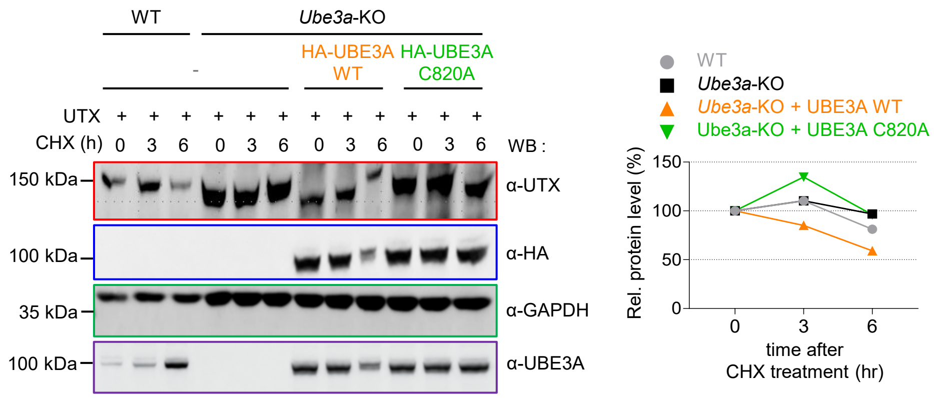

WT or *Ube3a*-KO mHypoE-N41 cell lysates co-expressing 6x Myc-UTX, HA-UBE3A WT, and HA-UBE3A C820A

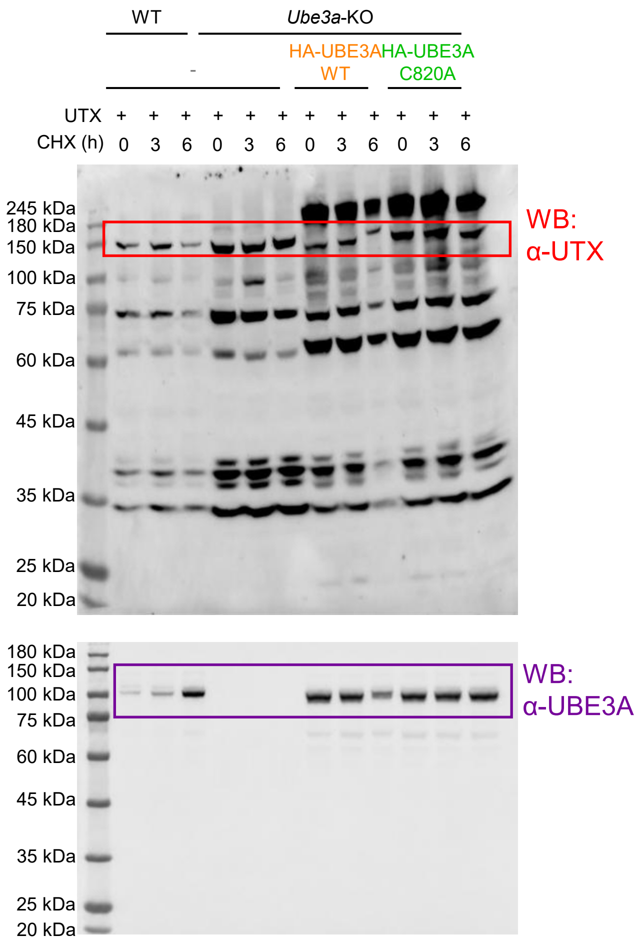

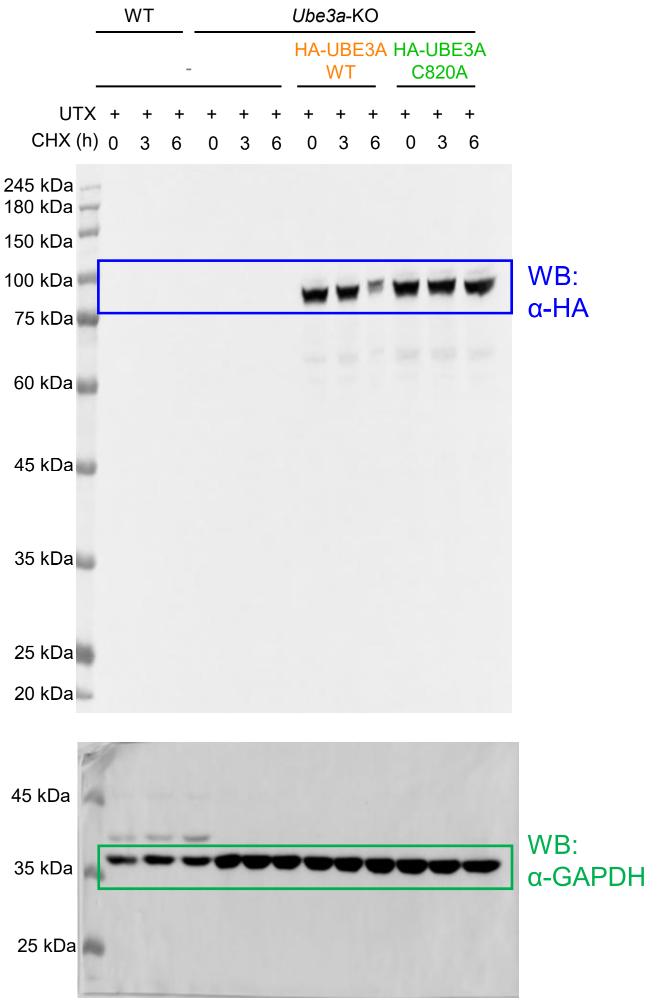

Supplement: Supplementary file 2 [file ADVS-9999-e78567-s002.zip › UBE3A Source Data/UBE3A Source data_unprocessed blots_upload.pdf]
